# TIP-INN: A thermodynamics-informed machine learning framework for the mechanical behavior of rocks

**DOI:** 10.1093/pnasnexus/pgag349

**Published:** 2026-10-09

**Authors:** Kangan Li, Tushar Mittal, Benjamin Holtzman, Seth Saltiel, Raphael Affinito, Loic Daridon

**Affiliations:** Department of Geosciences, The Pennsylvania State University, University Park, PA 16802, USA; Department of Geosciences, The Pennsylvania State University, University Park, PA 16802, USA; Lamont Doherty Earth Observatory, Columbia University, Palisades, NY 10964, USA; Department of Earth, Atmospheric and Planetary Sciences, Massachusetts Institute of Technology, Cambridge, MA 02139, USA; Department of Earth and Atmospheric Sciences, Cornell University, Ithaca, New York 14850, USA; Department of Geosciences, The Pennsylvania State University, University Park, PA 16802, USA; Department of Geomechanics and Scientific Drilling, GFZ Helmholtz Center for Geosciences, Telegrafenberg, Potsdam 14473, Germany; LMGC, Univ. Montpellier, CNRS, Montpellier, 34095 France

**Keywords:** constitutive model discovery, thermodynamics-informed neural networks, convex neural networks, general viscoelasticity, continuum damage mechanics

## Abstract

Modeling the mechanical deformation of geological materials across relevant thermodynamic conditions and dynamic processes remains a fundamental challenge in Earth sciences. Conventional rheological models rely on isolated disciplinary approaches and hard-coded iterative solvers that struggle with strongly coupled, nonlinear constitutive laws. Inverting macroscopic laboratory data to infer subgrid microphysics is also ill-posed, producing structural nonuniqueness and models that may violate thermodynamic laws during transient loading. To address these limitations, we present TIP-INN (Thermodynamics of Irreversible Processes Informed Neural Network), a thermodynamically consistent machine learning framework grounded in the generalized standard materials (GSM) formalism. TIP-INN discovers constitutive laws directly from time-series stress–strain data by learning two strictly convex thermodynamic potentials-the Helmholtz free energy and the dual dissipation potential-architecturally guaranteeing non-negative mechanical dissipation. A continuous, differentiable neural integrator solves internal state variable evolution as an initial-value problem, enabling stable, rapid training across complex rheologies. Benchmarked against existing thermodynamics-informed networks, TIP-INN trains 10 to 30 times faster than variational baselines and reduces extrapolation error by up to two orders of magnitude. We resolve the nonuniqueness of this inverse problem through two strategies: (i) diverse loading protocols (e.g. cyclic and stress-relaxation paths) that map the full geometry of the potential state space and (ii) a multimodal loss assimilating auxiliary seismo-acoustic data. TIP-INN thus rigorously bridges continuum damage mechanics and laboratory seismology. Finally, to ensure physical interpretability, the learned potentials are distilled via sparse identification of nonlinear potentials into closed-form expressions for efficient implementation in geodynamic codes.

Significance statement Predictive modeling of Earth systems-from earthquakes to geothermal reservoirs-is limited by empirical rock mechanics laws that fail to capture complex, transient deformation. We introduce a machine learning framework (TIP-INN [Thermodynamics of Irreversible Processes Informed Neural Network]) that embeds thermodynamic laws directly into its architecture, guaranteeing mechanical consistency by construction rather than by soft penalty. A novel aspect of this framework is that it can bridge continuum mechanics and observational seismology by assimilating data from the seismo-acoustic wavefield reflecting the evolution of internal rock structure to constrain unobservable internal rock damage. By combining thermodynamically diverse loading protocols with seismo-acoustic assimilation, TIP-INN provides a computationally efficient pathway for discovering unified constitutive laws governing the dynamic Earth, with direct applicability to tectonic deformation, geothermal energy, and earthquake physics.

## Introduction

The evolution of Earth systems-ranging from the flow of ice streams to mantle convection-fundamentally depends on how geological materials respond to changes in stress (including pressure), temperature, and chemistry. Consequently, the accuracy of large-scale geophysical models strongly relies on constitutive laws that can accurately map deformation to the stress tensor, σ, across diverse planetary conditions. In crustal tectonics, the formation of shear zones by strain localization is driven by feedbacks between microstructural evolution and mechanical strength, spanning brittle to ductile processes (1, 2). Volcanic eruptive dynamics are governed by the nonlinear rheology of magmas transitioning from fluid suspensions to granular solids with increasing crystallinity (3). Similarly, at Earth’s surface, subglacial slip behavior is modeled using a combination of viscous and plastic laws, directly affecting global sea level rise predictions (4). Despite their importance, deriving these constitutive laws is difficult because geophysics currently lacks a unified framework for material behavior. Instead, disciplines rely on isolated approaches for similar processes: glaciologists and geodynamicists generally use different empirical flow laws (5) for the same ensemble of processes. To describe localized, discrete stick-slip failure, seismologists use rate-and-state friction (6–9), while geodynamicists typically default to plasticity (a time-averaged, spatial-homogenized description of many discrete failures inside a representative volume) (1, 10), because of their different scientific aims. Beyond fundamental Earth science, bridging this disciplinary gap is also necessary for applied subsurface engineering applications related to energy extraction and storage. Focus areas such as Superhot Enhanced Geothermal Systems and geologic carbon storage require predictive models that operate well outside the steady-state conditions typical of many laboratory datasets (11, 12).

Generalized constitutive models for geologic solids must be capable of spanning diverse thermodynamic regimes and the entire mechanical spectrum of deformation processes and microstructural evolution. Geological materials can exhibit profound mechanical shift across relatively small ranges of conditions, for example, transitioning from localized brittle cracking in the shallow crust to distributed ductile creep at greater depths, or even in response to different forcing rates on the same rock volume. Capturing these complex brittle-ductile transitions requires bridging a large set of length scales and integrating a range of processes. Instead of attempting to model every individual micro-crack or dislocation glide (13), one common theoretical approach is to represent these sub-grid processes using internal state variables (ISVs) averaged over a representative elementary volume (REV). These variables can track some aspect of the microstructure, but also partitioning among different mechanisms. By tracking variables such as scalar damage (*α*) for brittle degradation and the visco-plastic strain tensor ($\varepsilon^{p}$) for ductile flow, distinct micromechanisms can be integrated into a single continuum model. The generalized standard materials (GSM) framework (14, 15) is one such formalism for deforming nonlinear solids, utilized here. At the macroscale (km), solving the conservation laws (e.g. mass–momentum–energy conservation) requires constitutive closures that couple the evolution of these ISVs to macroscopic stress and strain. Unlike homogeneous engineered materials, crustal rocks are intrinsically multimineralic and multiscale, making the derivation of these constitutive closures nontrivial and often nonunique.

Deriving these closures presents two main physical challenges. First, conventional approaches often rely on “patchwork” multiphysics models that couple disparate empirical laws and use ad hoc or simplified criteria to switch between elastic, plastic, and viscous regimes. Because these models lack a unified energy budget, they can inadvertently violate the second law of thermodynamics especially during transient loading, potentially predicting physically impossible states like negative mechanical dissipation (16, 17). Second, a fundamental scale gap exists between macroscopic observations and subgrid defect dynamics. Because standard laboratory measurements of macroscopic stress and strain cannot uniquely constrain internal microphysics, researchers typically preselect rigid phenomenological models-such as specific combinations of viscoelastic spring-and-dashpot elements (e.g. Maxwell, Kelvin–Voigt, or Burgers) to fit data. This rigidity introduces a strong structural bias. While one can perfectly match an experimental curve by arbitrarily adding mechanical elements, this over-parameterization makes the resulting rheology highly dependent on the initial model choice. It obscures the true, often sparser physics driving the deformation and yields empirical fits that fail to extrapolate reliably beyond the laboratory (18). Addressing this challenge requires a data-driven constitutive model discovery method that is mathematically flexible yet strictly obeys thermodynamic laws, capable of capturing complex damage mechanics without presupposing a specific physical structure.

Machine learning (ML) offers a potential solution to these bottlenecks, but many data-driven methods struggle with physical consistency. Within the broader framework of physics-informed neural networks (PINNs) (19), we face a fundamental methodological trade-off between soft-penalty and architectural constraint enforcement (20). Soft-penalty methods, which append physical constraints to the loss function, are universally applicable but require empirical weight tuning, suffer from gradient-magnitude pathologies (21), and only satisfy constraints approximately at training points. When applied to geological media, such models frequently predict physically impossible states during extrapolation, such as spontaneous crack healing or negative dissipation (see the rate-state friction case in Section S16) (22). Conversely, architectural enforcement embeds constraints directly into the network’s architecture, allowing for the exact, global satisfaction of physical laws. While thermodynamically consistent architectures utilizing input convex neural networks (ICNNs) (23) have successfully modeled engineered materials like metals and elastomers (24–32), they have not yet been adapted for the complex, multimineralic nature of rocks.

To address these methodological gaps, this study introduces the Thermodynamics of Irreversible Processes-Informed Neural Network (TIP-INN). Built on the differentiable programming (∂P) paradigm (33), TIP-INN discovers constitutive laws by learning two strictly convex scalar thermodynamic potentials: the Helmholtz free energy, *Ψ*, and the dual dissipation potential, $\Phi^{*}$. By parameterizing these potentials with strictly convex neural networks (23), TIP-INN guarantees thermodynamic consistency by construction. Automatic differentiation of these potentials yields the thermodynamic driving forces, $X^{d}=-\partial \Psi /\partial \alpha$, governing the evolution of the internal state variables while ensuring positive intrinsic dissipation (D≥0) across all predictions. This architectural enforcement of thermodynamic constraints—as opposed to soft loss-penalty approaches—is critical for stability and the reliability of extrapolation beyond laboratory training datasets.

Furthermore, classical constitutive updates for complex materials rely on iterative numerical solvers (such as Newton–Raphson) that require the derivation of algorithmic tangents, are hard-coded to specific formulations, and frequently fail to converge under complex transient loading. To overcome this, TIP-INN treats the constitutive update as an initial-value ordinary differential equation (ODE) solved via a fully differentiable neural integrator (34, 35). This end-to-end continuous gradient propagation circumvents the computational overhead and convergence failures of hard-coded solvers, enabling faster, more stable training over complex deformation histories as long as we do not consider structural state transitions and stiffness of the mechanical evolution equations. Because the integrator is fully differentiable, the network intrinsically learns path-dependence, hysteresis, and fading memory-features that are critical for geological deformation (36) but mathematically invisible to standard feed-forward architectures for learning stress–strain data pairs.

Crucially, to resolve the macroscopic scale gap, we can integrate multi-modal seismo-acoustic data-such as acoustic emissions (AE), seismic and ultrasonic wave velocities ($V_{p}$, $V_{s}$), and waveform features-directly into the TIP-INN model’s objective function (37, 38). While macroscopic stress alone leaves the evolution of internal variables unconstrained, seismo-acoustic data provides a direct, observable proxy for sub-grid defect dynamics and the internal damage state. Motivated by rapid advances in observational geophysics-including distributed acoustic sensing (DAS) and microearthquake monitoring-this multimodal assimilation bridges the gap between lab-scale mechanics and field-scale observables. Combined with high-variance thermodynamic training paths (e.g. cyclic loading, stress-relaxation), this strategy effectively regularizes the ill-posed inversion of the unobservable ISVs.

Finally, despite their flexibility, high-dimensional neural networks are inherently difficult to interpret and often function as opaque “black boxes.” To recover the underlying analytical functions (and hence infer the physics), we introduce a sparse identification of nonlinear potentials (SINP) approach, adapted from the SINDy framework (39). SINP projects the trained neural potentials onto a constrained, strictly convex library of analytical functions, distilling the network into closed-form equations. Although inspired by SINDy, SINP is not a direct application of it: rather than fitting an unconstrained regression to the system dynamics, SINP operates on the thermodynamic potentials and embeds convexity and non-negativity constraints in the identification itself, so that the distilled analytical law is thermodynamically admissible by construction. Because this projection preserves thermodynamic admissibility, it yields explicit mathematical expressions for the nonlinear coupling between macroscopic deformation and the evolution of internal variables. By recovering the exact functional forms of the free energy and dissipation potentials, we can directly analyze the specific damage mechanics and rheological transitions the network learned from the data. Moreover, these distilled analytical laws can be used in existing computational geodynamics codes (40).

In this study, we apply the TIP-INN framework to synthetic models (with known underlying potentials) and complex, multimodal laboratory datasets to address four specific scientific questions regarding constitutive discovery in rock mechanics:

1. Does the end-to-end differentiable neural ODE framework provide a scalable, fast, and stable approach for modeling geologically relevant behaviors?
2. Given the structural nonuniqueness of the constitutive model state space, what diversity of experimental loading paths (monotonic, cyclic, stress-relaxation) is required to capture the true geometry of the thermodynamic potentials?
3. Can the integration of ancillary seismo-acoustic data effectively regularize the ill-posed inversion of unobservable internal state variables in real, heterogeneous rocks?
4. Can high-dimensional neural potentials be distilled into closed-form, mathematically interpretable analytical equations using a thermodynamically constrained sparse regression (SINP)?

TIP-INN currently targets materials with infinitesimal strain and convex Helmholtz free energy *Ψ*. While this restriction is well-suited to modeling distributed damage, viscoelasticity, and viscoplasticity in geomaterials, it deliberately excludes structural phase transitions and certain spatially complex forms of damage. Capturing such nonconvex phenomena requires relaxed-envelope or nonlocal formulations. The extension of this data-driven framework to nonconvex energy landscapes via polyconvex envelopes or bounded-variation regularized formulations remains a natural and important future direction, depending on the requirements from data-model mismatches.

The remainder of this article is organized as follows. Results section evaluates the accuracy and robustness of the proposed computational framework by validating it against analytical models and demonstrating its capability to discover rheological models from experimental data. Discussion section discusses the implications of this framework for upscaling to crustal-scale geophysical models. We conclude our work in Conclusion section. The theoretical background and detailed neural architecture are provided in Materials and methods section.

## Overview of TIP-INN

The GSM formalism constitutes a robust framework for constitutive modeling of solids, combining elasticity with a broad range of linear and nonlinear dissipative mechanisms. The GSM is built upon fundamental thermodynamic energy potentials (e.g. the Helmholtz free energy, *Ψ*) and the Dissipation potentials *Φ* (formally pseudopotentials), which encode energy dissipation as functions of the *fluxes* of state variables, in a complementary construction to the energy potentials. Importantly, the gradients of these potentials define the conjugate thermodynamic forces governing the material’s microstructural evolution. The free energy determines the reversible internal stresses (the energetic driving forces, $A_{\alpha }$), while the dissipation potential dictates the corresponding dissipative stresses (the resisting viscous or plastic drag forces, $X^{d}$). The strict balance between these internal stresses enforces local thermodynamic equilibrium. The detailed derivations and the physical interpretation of these terms within the context of specific rheological models are provided in Materials and methods section and Supplementary material.

One key advantage of working directly with thermodynamic potentials—rather than empirical stress–strain relationships—is that the material response of generalized solids is governed by time-evolution equations derived strictly from the derivatives of these potentials (see Materials and methods section). Similarly, macroscopic material properties (e.g. elastic modulus, heat capacity, viscosity, etc.) can be self-consistently derived based on the Hessian of the potentials (second derivatives with respect to the state variables for *Ψ* and their forces or fluxes for *Φ*, explained further in Materials and methods section). Thus, even if the model is trained exclusively on macroscopic stress–strain pairs, we can still mathematically represent the coupled underlying processes by learning a complete set of potentials, thereby capturing the nonequilibrium evolution of ISVs as paths through the two potentials.

To operationally use this theory, we introduce a thermodynamically consistent neural network framework, the Thermodynamics of Irreversible Processes Informed Neural Network (TIP-INN), which pairs a neural ODE integrator (25, 35) with two parallel, strictly convex neural networks (see Fig. 1). These networks are tasked with predicting the geometry of the Helmholtz free energy (*Ψ*) and dual dissipation potential ($\Phi^{*}$) surfaces under isothermal, infinitesimal strain conditions. Consequently, the training process becomes significantly faster and more robust across different rheologies using the exact same architecture. Provided the numerical time steps are sufficiently small, both inference and internal state evolution reduce to a single forward pass through the network, entirely circumventing iterative convergence failures. We can use this directly into an ODE solver (like torchdiffeq), rollout the trajectory, compute a loss against the true data, and backpropagate using the adjoint method to adapt the NN weights. Ultimately, the TIP-INN architecture enables the discovery of complex rheological models directly from sparse experimental datasets, such as stress–strain time series and seismo-acoustic data. By effectively assimilating diverse experimental loading paths (monotonic and cyclic), the framework captures the underlying geometry of the thermodynamic potentials, resulting in accurate, thermodynamics-obeying constitutive models.

**Figure 1:**
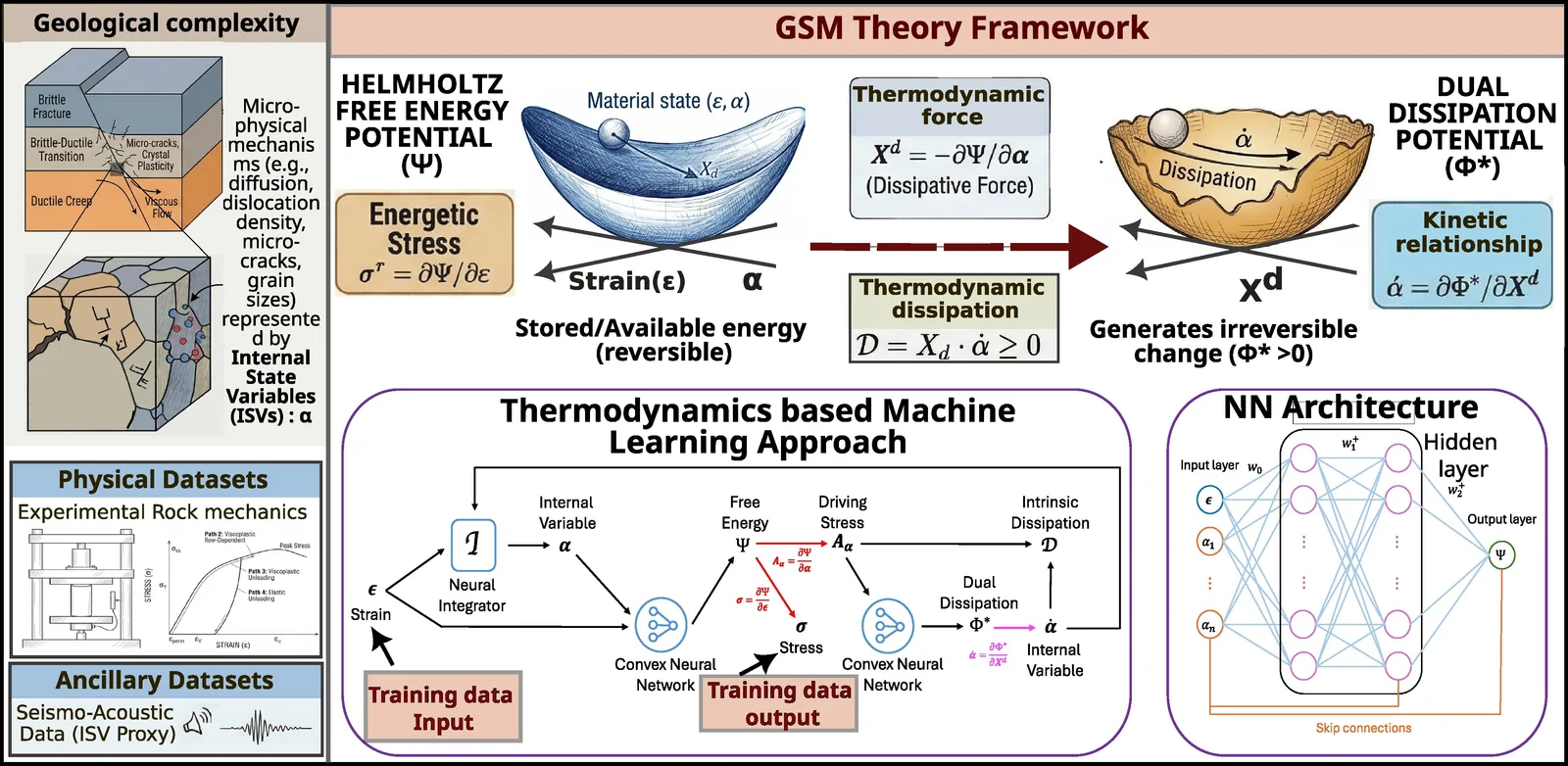
Schematic illustration of the TIP-INN for constitutive modeling. Left: The key challenge with constitutive modeling for geophysical systems that span a range of thermo-mechanical conditions and microscale deformation processes as well as the lab datasets for rock mechanics. Top: Schematic illustration of the primary concept associated with the GSM theory related to using a set of two potentials to represent the evolution of the system. Bottom Left : The overall TIP-INN computational framework builds upon a “Neural Integrator” to solve the evolution equation and “Convex Neural Networks” to predict the Helmholtz free energy, *Ψ*, and dual dissipation, $\Phi^{*}$. Bottom Right: The detailed architecture of a convex neural network used for learning the constitutive model.

## Results

### Accuracy and training data diversity: the Maxwell model

Before applying TIP-INN to complex experimental rock data, we first demonstrate its fundamental capability to discover hidden, but known, thermodynamic structures. We emphasize a core achievement of this framework: even when trained exclusively on macroscopic stress–strain data pairs, TIP-INN successfully reconstructs the true, unobservable geometry of both the Helmholtz free energy (*Ψ*) and the dual dissipation potential ($\Phi^{*}$) surfaces. To rigorously validate this accuracy and robustness, we utilize potentials derived from standard analytical constitutive models—specifically, the Maxwell and Burgers viscoelastic models (see rheological representations in Fig. S1)—which provide a known ground truth for these potentials. As a necessary first step, we utilize a standard Maxwell viscoelastic model (Young’s modulus $E_{v}=2$ GPa and viscosity $\eta_{v}=1$ GPa s) to systematically investigate the diversity of loading conditions strictly required for effective thermodynamic training. We consider two distinct strain-controlled loading modes: cyclic loads and monotonic loads (both with and without oscillations).

First, evaluating monotonic strain path, Fig. 2 demonstrates that training TIP-INN on an oscillatory strains and testing it on a nonoscillatory strains produces more accurate *Ψ* predictions than the reverse. We see similar results when comparing simple step-wise monotonic paths compared to more stochastic paths that sample a larger range of forcing frequencies and history dependence. This asymmetry occurs because the oscillatory load forces the network to explore a broader region of the material’s thermodynamic state space. Second, training with a single cyclic load yields an accurate *Ψ* for other cyclic tests of differing frequencies and magnitudes, but its precision degrades significantly when tested against a monotonically oscillatory load with large viscous strains. Third, training exclusively with a monotonically oscillatory load yields an accurate *Ψ* for cyclic tests but fails to extrapolate accurately to other monotonic tests with higher viscous strains.

**Figure 2:**
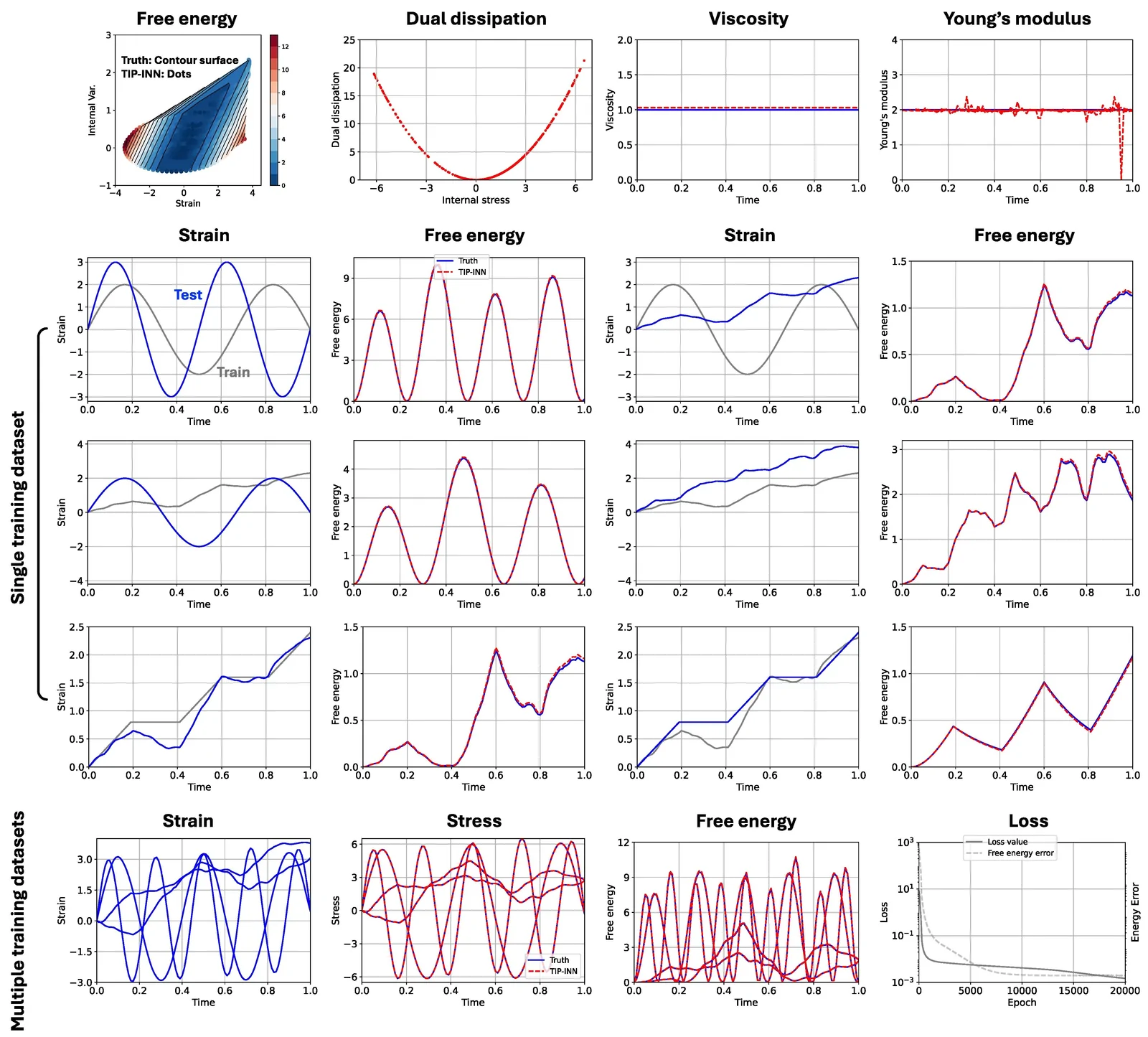
Maxwell viscoelastic model: top row: predicted Helmholtz free energy (*Ψ*) and dual dissipation ($\Phi^{*}$) with convex and positive properties. The smooth contoured surface is the known potential and the dots are the learned values. For the Maxwell model, the free energy is a function of the total strain and viscous strain (internal variable). The predicted viscosities and Young’s moduli derived from *Ψ* and $\Phi^{*}$ are also plotted. Middle row: Predicted *Ψ* for different loading types by training TIP-INN with a single load. Note that the predicted stresses (σ) are accurate in all cases and therefore omitted (see Fig. S2). Bottom row: Predicted σ, *Ψ*, and $\Phi^{*}$ for the testing data by training TIP-INN with multiple cyclic and monotonically oscillatory loads. The loss values and errors on *Ψ* are plotted against No. of epochs as a reference. Additional results, including the strain, predicted stress, and free energy for the training data, as well as a spectral analysis of the strain across both the training and testing datasets, are provided in Figure S3.

These results show how little data are needed to train a TIP-INN, because it is so highly constrained. Also, they highlight the benefits of training on diverse, multimodal loading types to improve model transferability. As shown in Fig. 2, training TIP-INN with a combined ensemble of five cyclic and five monotonically oscillatory loads consistently yields highly accurate stress and free energy predictions across all testing data, outperforming single-load training. The convex and positive properties of *Ψ* and $\Phi^{*}$ remain well-preserved. Most importantly, because TIP-INN learns the true geometry of the thermodynamic potentials rather than merely memorizing empirical stress–strain curves, it correctly predicts the underlying viscosity and Young’s modulus. The minor oscillations in the predicted Young’s modulus are numerical artifacts attributed to errors in computing the second-order derivatives of the free energy. These results carry direct, critical implications for experimental rock mechanics: discovering robust constitutive laws requires diverse experimental loading protocols to adequately sample the internal thermodynamic state space. Additional predicted results for the training data are provided in Fig. S3.

### Structural nonuniqueness and the ill-posed inverse problem: the Burgers model

Having established that diverse loading paths are required to capture the true geometry of thermodynamic potentials, we now investigate how structural nonuniqueness affects constitutive discovery. We test the accuracy of TIP-INN on the more complex Burgers viscoelastic model with prescribed material properties ($E_{v_{1}}=1.0$ GPa, $\eta_{v_{1}}=2.0$ GPa s, $E_{v_{2}}=2.0$ GPa, and $\eta_{v_{2}}=0.5$ GPa s). The theoretical Burgers model contains two ISVs associated with the viscosities $\eta_{v_{1}}$ and $\eta_{v_{2}}$. However, because TIP-INN does not know the exact rheological representation a priori, we test the network by assuming either one or two internal variables in the system, utilizing the robust, diverse training protocols established in the previous section.

Figure 3 shows the predicted stress, Helmholtz free energy, and dual dissipation from TIP-INN across the testing data. The predicted macroscopic stresses are nearly indistinguishable from the true theoretical solutions, regardless of whether one or two ISVs are assumed. However, accurate Helmholtz free energy geometries are obtained *only* when a single ISV is assumed; large errors arise when two ISVs are introduced, particularly for monotonically oscillatory loads with high viscous strains. This discrepancy occurs because the neural network effectively approximates the linear Burgers model (two ISVs) by learning a highly nonlinear Maxwell model (one ISV). This structural compensation is confirmed by the strong nonlinearities observed in the predicted Young’s modulus for the single-ISV case (see Fig. S4). Incorporating a second ISV introduces uncontrollable parameters into the network because the standard loss function (Eq. 17) is bounded only by macroscopic stress, leaving the ISVs unconstrained. Consequently, the network predicts less accurate thermodynamic potentials (*Ψ* and $\Phi^{*}$), and large deviations emerge between the predicted and true material properties. While this reduced performance directly reflects the ill-posedness of inferring multiple rheological parameters and underlying models from stress–strain data alone, it points to the need for more elaborate approaches to sparsity constraints, training strategies, and auxiliary data to augment model separation as discussed later in this article. Additional predicted results for the training data are provided in Fig. S5.

**Figure 3:**
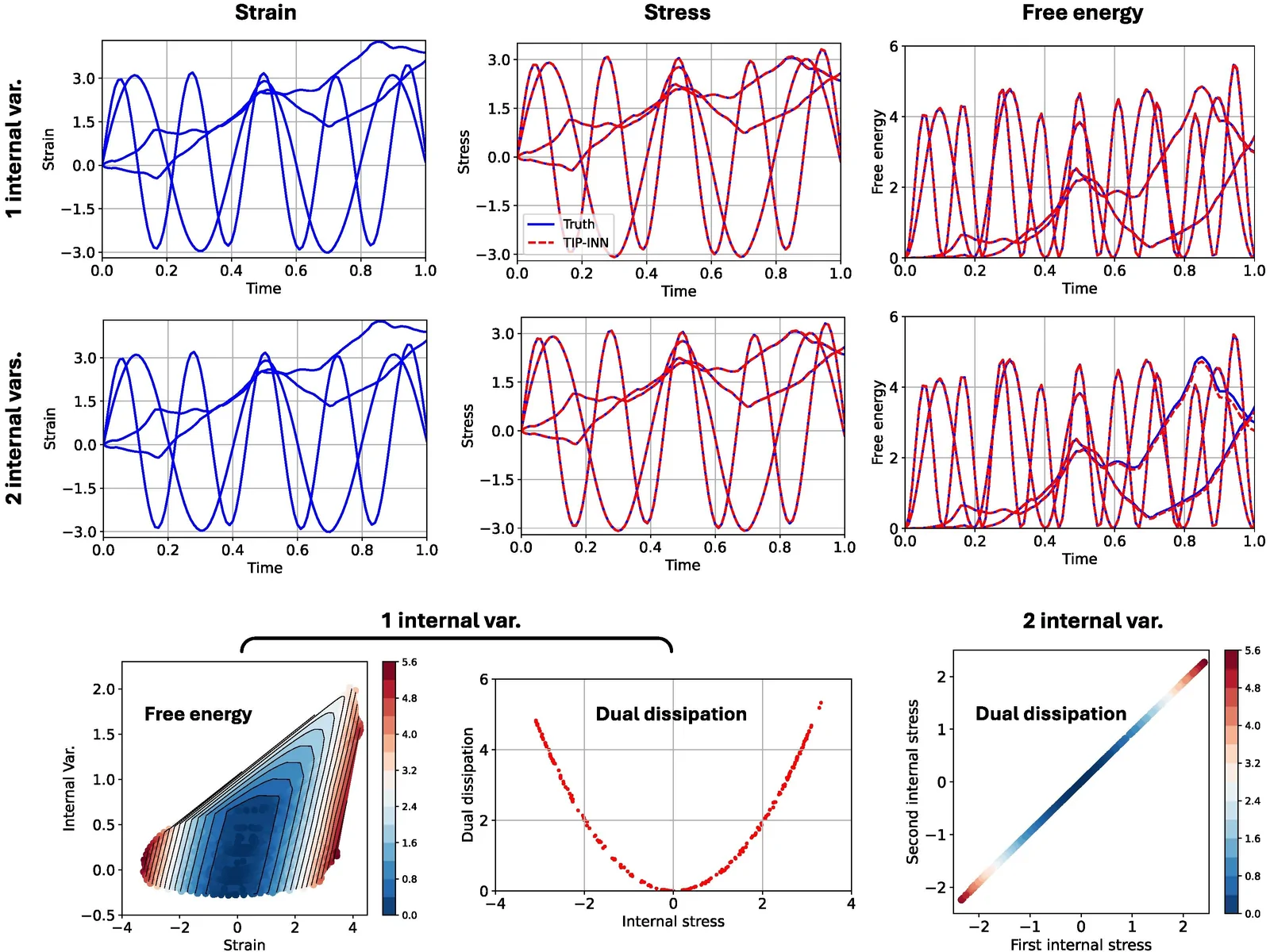
Burgers viscoelastic model: predicted stress (σ), Helmholtz free energy (*Ψ*), and dual dissipation ($\Phi^{*}$) for the testing data of TIP-INN by assuming one or two internal state variables in the system. Both strain-controlled loading types: cyclic and monotonically oscillatory loads, are considered in training and testing. At the bottom row, *Ψ* and $\Phi^{*}$ are plotted with respect to the strain/internal variables and internal stresses (the dissipative driving force, $X^{d}$), respectively. The colored surface and dots are the true and predicted *Ψ*/$\Phi^{*}$, respectively. Additional predicted results for the training data are provided in Fig. S5.

### Discovering rheological models from experimental data

We next apply TIP-INN to discover rheological models directly from experimental data. The dataset is derived from a uniaxial compression test performed on a porous cylindrical rock sample (p5914, 1 inch core, and 2.5 inch long) cored from lava flows exposed along the southeast avalanche scarp of Pacaya volcano (41). The experiment was conducted at the Penn State Rock Mechanics Deformation Apparatus under unconfined uniaxial loading at room temperature. Axial deformation was applied under displacement control along a monotonically increasing loading path. Servo-controlled hydraulic feedback was achieved using direct current displacement transducers (DCDTs) with a resolution of ±0.1μm and a custom-built beryllium-copper load cell with a resolution of ±5 N. Throughout deformation, the sample response was monitored both mechanically and acoustically.

To enable ultrasonic monitoring, the steel loading platens were fitted with in-house-built broadband P-wave lead–zirconate–titanate (PZT) transducers with a central frequency of 500 kHz. These transducers allowed simultaneous recording of acoustic emissions, which capture internal defect dynamics, and active ultrasonic pulsing to monitor changes in wave speed and amplitude during deformation. A thin layer of molasses was applied between the sample ends and the loading platens to ensure consistent acoustic coupling. The acoustic feature-extraction and coda-wave processing pipeline used for the analyses that follow is detailed in Section S7.

Figure 4 illustrates the strain/loading-controlled loading path. As the macroscopic load increases, microfractures initiate, propagate, and coalesce within the sample. This accumulation of internal damage correlates with a degradation in the measured acoustic velocity ($V_{p}$), which we use as an observable proxy for the internal damage state. We explicitly incorporate $V_{p}$ into the TIP-INN loss function (Eq. 17), constraining the evolution of one ISV to match the observed acoustic trajectory. Because rock deformation involves multiple overlapping micromechanisms (e.g. stiffness degradation and fracture propagation), we evaluated TIP-INN by varying the total number of assumed ISVs, following the structural testing approach outlined in Structural nonuniqueness and the ill-posed inverse problem: the Burgers model section. Figure 4 compares the predicted macroscopic stress and the discovered Helmholtz free energy assuming one, two, or three ISVs. With the acoustic velocity constraint active, increasing the number of ISVs from one to three yields progressively more accurate stress predictions. This indicates that multiple ISVs are necessary to capture the strong nonlinearities induced by progressive fracture damage. The model that utilizes three ISVs alongside the acoustic constraint achieves the highest accuracy in the final stages of the experiment, when macroscopic fractures dominate the deformation. However, further increasing the number of ISVs to four, or attempting to constrain an additional ISV using acoustic coda wave data, degrades the accuracy of the predicted macroscopic stress. A detailed analysis of these extended ISV configurations and acoustic constraints is provided in Section S6. This demonstrates the utility of multimodal seismo-acoustic data for regularizing the ill-posed inversion of unobservable ISVs. Furthermore, training TIP-INN with the neural ODE integrator remained computationally stable throughout the highly transient experimental data, bypassing the convergence failures frequently encountered with classical Newton-based algorithms under extreme loading.

**Figure 4:**
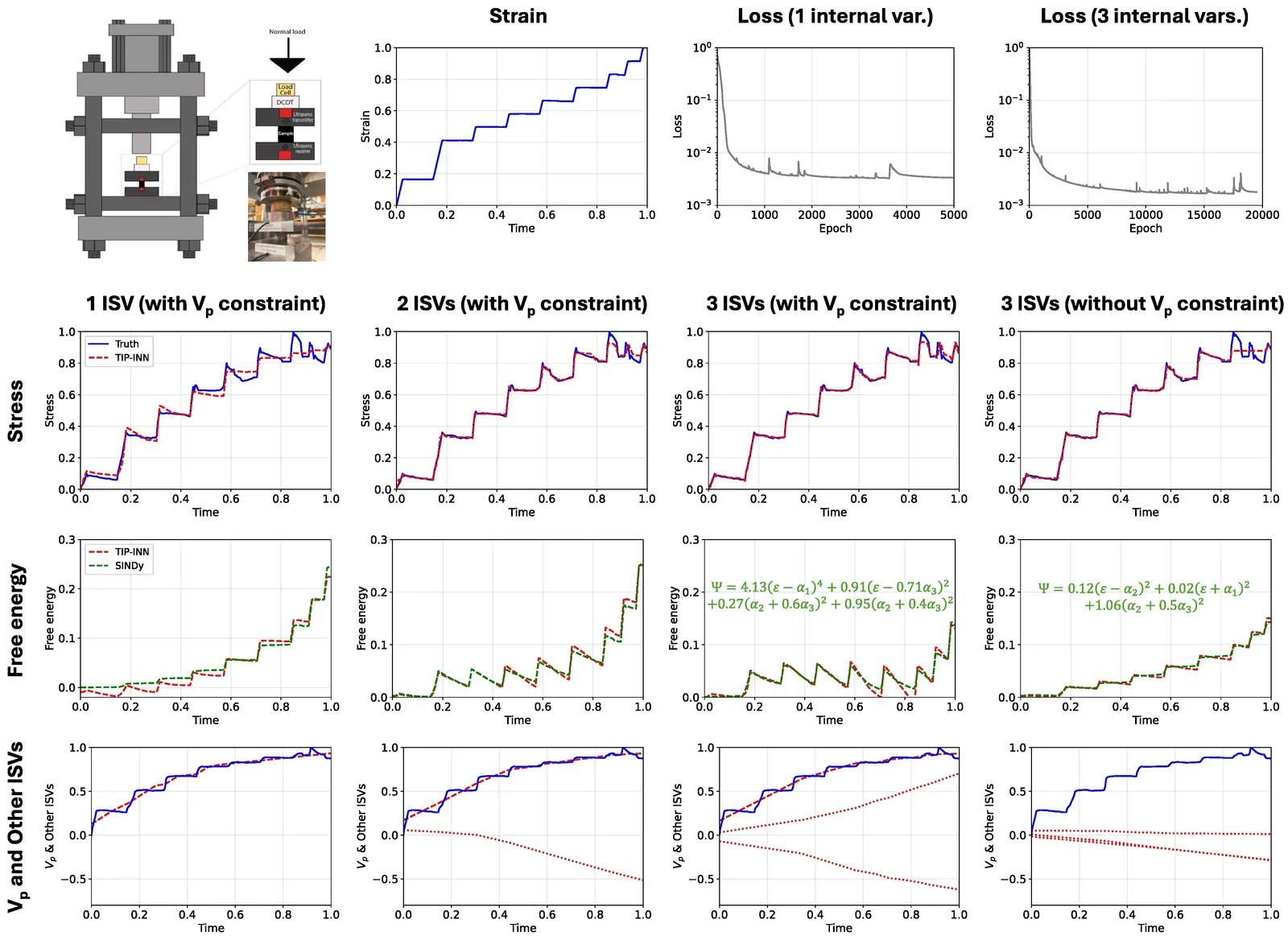
Discovering a rheological model from experimental data: Top row: experimental setup, applied strain-controlled load, and loss values vs. No. of epochs in the training of TIP-INN. Bottom three rows: Predicted stress, Helmholtz free energy, and internal state variables for TIP-INN with different numbers of internal state variables. Calibrations of the predicted Helmholtz free energy with SINP are also plotted for comparison.

### Comparison to a nonlinear viscoelastic baseline

To assess the effect of thermodynamic consistency, we compared TIP-INN against a baseline model, a parametric nonlinear visco-elastic Maxwell model with nonlinear elasticity and viscosity (Section S15). We trained the model using Bayesian MCMC regression on the same experimental dataset using an identical data-driven protocol (applied strain as input, stress as output). As shown in Fig. S16, the baseline model fails to reproduce the experimental stress-strain response during both the initial loading and the fracture-dominated final stage of loading. It exhibits systematic deviations unlike TIP-INN, which is regularized by thermodynamic constraints and acoustic velocity and maintains high predictive accuracy. This divergence underscores that the strictly convex potential structure and seismo-acoustic regularization within TIP-INN are essential mechanisms. They enable the network to generalize across the full loading history and to stably predict macroscopic failure, whereas purely unconstrained empirical fits with phenomenological models struggle to fit the data and are highly susceptible to large extrapolation errors.

### Extracting interpretable physics: SINP

To overcome the black-box nature of the trained neural potentials, we apply a SINP procedure-adapted from the SINDy framework (39)-to distill the learned Helmholtz free energy into closed-form analytical expressions. We construct a constrained, strictly convex basis library Θ(ε,α) comprising polynomial and exponential functions, and solve a non-negative least squares (NNLS) regression to identify the parsimonious combination that approximates the neural potential while preserving convexity. This approach works well for simplified analytical potentials, and can recover an exact sparse analytical form with a few terms even if the library has 100 s of candidate terms.

Figure 4 compares the SINP-calibrated *Ψ* against the neural predictions. SINP captures the general trends of the predicted *Ψ*, though it struggles to reproduce the detailed fine structure for the most complex (three internal variable) case using the current convex analytical basis. This observation is itself physically informative: it indicates that the rheology underlying the experimental data involves nontrivial nonlinear cross-couplings between strain and damage variables that go beyond standard polynomial damage-mechanics potentials. The critical novelty of SINP relative to standard SINDy is that it is applied to the *thermodynamic potentials* rather than to the dynamics directly, thereby preserving the thermodynamic admissibility achieved by TIP-INN. This is more than a change of target variable. Whereas SINDy fits an unconstrained sparse regression against a generic function library, SINP restricts the library to strictly convex, positive candidate functions and enforces the sparse selection through a non-negative least squares solve, so that every retained term contributes a convex, thermodynamically admissible piece of the potential by construction. The convexity and non-negativity constraints are therefore built into the identification itself rather than imposed after the fact, which makes SINP a constrained, structure-preserving procedure rather than a direct application of SINDy. In cases where the SINP-distilled analytical form is sufficiently accurate (see Fig. S15), it provides a closed-form constitutive law that can be directly implemented in standard finite element codes (40)-delivering the computational efficiency of a hard-coded model with the discovery power of machine learning.

## Discussion

In this study, we have presented a novel approach to machine-learning constitutive models for complex solids. By strictly employing convex neural networks, the positive, convex properties of both thermodynamic potentials (*Ψ* and $\Phi^{*}$) are enforced by the architecture. This architectural convexity also carries a genuine topological consequence: every sublevel set {Ψ≤c} is convex and therefore simply connected, so the learned potential admits a single connected set of minimizers with no spurious disconnected basins. This yields more accurate stress predictions and thermodynamic-potential geometries than earlier ML approaches (e.g. Masi and Einav (35)), which relied on nonconvex networks and consequently predicted physically impossible negative free energies for simple Maxwell models. Our explicit introduction of the dual dissipation potential rigorously captures the internal driving forces, yielding a thermodynamic framework that captures the coupling of the GSM between the potentials ($X^{d}=-A_{\alpha }$). Our numerical results also answer a critical question regarding experimental design: diverse training data spanning multiple frequencies and modes (cyclic, monotonic, and stress-relaxation) sample the potential space more broadly than simple forcing paths. Future rock deformation experiments should deliberately incorporate these diverse loading protocols to adequately map the geometry of the underlying potentials and reduce the nonuniqueness of constitutive inversion. The importance of this for rock mechanics is direct. Constitutive laws for rocks have historically been phenomenological forms calibrated to a narrow set of experiments, with no guarantee of thermodynamic admissibility away from the calibration path and little principled basis for extrapolation across the wide range of strain rates and loading histories encountered in the Earth. By enforcing the second law architecturally and by recovering interpretable potentials rather than opaque stress–strain maps, TIP-INN yields constitutive models that stay physically admissible under unseen loading and that can be inspected, distilled, and deployed, which is what makes thermodynamically consistent discovery worth pursuing for geomaterials specifically. In this section, we discuss lessons learned and future directions for the TIP-INN.

### Number of internal variables for constitutive modeling

Our framework clarifies the role of ISVs in data-driven discovery. As demonstrated by the Burgers model (Structural nonuniqueness and the ill-posed inverse problem: the Burgers model section), increasing the number of ISVs without internal constraints degrades the accuracy of the predicted potentials. In stark contrast, capturing the strong nonlinearities of the experimental rock data (Discovering rheological models from experimental data section) strictly required multiple ISVs to adequately represent the true internal driving forces. This discrepancy highlights the necessity of multimodal data assimilation. By using the acoustic velocity ($V_{p}$) as an active constraint on the damage ISV, we provide a physically motivated regularization that anchors the network. This demonstrates that integrating ancillary seismo-acoustic (or other) data may be required to resolve the ill-posed inversion of unobservable microstructural dynamics. We note, however, that mapping ultrasonic dynamic velocities to static deformational ISVs requires careful consideration, since velocity can be sensitive to damage (as a proxy, in the form we use here) or could be computed directly as a constraint based on the elastic modulus derived from the free-energy potential. Nonetheless, even $V_{p}$ alone serves as an effective, physically observable regularizer for the damage trajectory and highlights the potential for using other seismo-acoustic features, especially related to the coda waves, as proxies for internal variables.

The question of *which* acoustic feature should constrain *which* ISV, and how to make that assignment nonarbitrary, is the central methodological issue this multimodal setting raises. We address it directly in the Supplementary material (Section S8, Fig. S9) with a partial-correlation discriminator, built on the Frisch–Waugh–Lovell construction (42, 43), that residualizes each ISV and each candidate acoustic feature on $V_{p}$ and axial strain before correlating, so that a shared loading trend cannot masquerade as a physical link. The analysis of the experimental sample points to a staged physical picture in which the three acoustic observables become diagnostic of three distinct micromechanical processes in turn (Section S9). The direct-wave velocity $V_{p}$ tracks the elastic-stiffness coordinate, the curvature $\partial^{2}\Psi /\partial \epsilon^{2}$ of the stored energy, through progressive crack closure under rising mean stress; this is the earliest and most robust link. The dominant frequency of the transmitted pulse then tracks the free internal coordinate, since it is governed by scattering and attenuation on and near the fast, near-direct path, together with the evolving spatial distribution of damage, rather than by the bulk modulus. The coda δv/v, which multiply samples the whole specimen volume, becomes diagnostic only later, once damage is extensively and heterogeneously distributed rather than concentrated near the fast path (Fig. S10). Consistent with this staged reading, the coda-sensed velocity turns over late in the record while the first arrival keeps stiffening, coinciding with a change in how the model uses its free internal coordinates, which indicates that the learned internal state and the measured acoustic field respond coherently to a single micromechanical transition (Fig. S11). Because the discriminator also shows that this acoustic-to-ISV link is reproducible under interpolation but nonstationary across the loading path, we use the acoustic channel as a soft, feature-specific regularizer rather than a hard constraint. This mapping is best understood as a two-way street, and much of its value lies there: the thermodynamic framework assigns each acoustic observable a concrete physical meaning in terms of a specific micromechanical process, namely elastic stiffening for $V_{p}$, near-path scattering for the dominant frequency, and volume-distributed damage for the coda, while the same observables in turn give a physical identity to internal coordinates that are otherwise unobservable. This correspondence is many-to-one and stage-dependent rather than a clean one-to-one assignment of every acoustic feature to a single ISV, which is precisely why a weak, feature-specific penalty is the appropriate coupling. When we instead impose a hard acoustic equality, for example forcing the coda as a strict constraint, the latent state is over-determined and the macroscopic stress fit degrades (Section S6, Fig. S8), which confirms that soft supervision is a requirement rather than a convenience for observables that each carry several overlapping influences. We accordingly set out a gauge-symmetric weak-supervision architecture for turning this soft coupling into a transferable constraint as future work (Section S10).

### Computational advantages over variational approaches

A key practical advantage of TIP-INN over incremental variational formulations, such as the generalized standard material network (GSMN) of Flaschel et al. (25), is the simultaneous achievement of computational efficiency and superior extrapolation accuracy. GSMN requires solving a local Newton–Raphson minimization problem at every time step during both training and inference. In our benchmarks, this variational approach is 10 to 30× slower per training epoch than TIP-INN’s neural integrator, which reduces each constitutive update to a single differentiable forward pass. This cost difference fundamentally limits the ability to perform the systematic hyperparameter sweeps essential when the constitutive model form is unknown a priori. Furthermore, while GSMN and TIP-INN achieve comparable accuracy on training data, TIP-INN demonstrates significantly greater flexibility and robustness during extrapolation. In viscoelastic (Maxwell) benchmarks, TIP-INN reduces the testing $L_{2}$ errors by approximately two orders of magnitude compared to GSMN, likely because the rigid Newton iterations in variational networks over-constrain the model during the transient loading paths typical of geological deformation. In contrast, alternative formulations such as TANN (31), which completely forgo architectural convexity in favor of soft-penalty thermodynamic enforcement, suffer from severe overfitting. TANN yields stress and free energy prediction errors two to three orders of magnitude larger than TIP-INN. Ultimately, our Neural ODE formulation avoids the convergence failures of local root-finding while preserving strict thermodynamic consistency, resulting in a fast, robust, and highly generalizable constitutive discovery framework. More details on the comparison of different methods for the Maxwell viscoelastic and Perzyna viscoplastic models are provided in the Section S13.

### Interpretability through SINP and general utility

The SINP procedure (Sparse identification of nonlinear potentials section) provides a reliable pathway from black-box neural potentials to interpretable, closed-form analytical constitutive laws. While the current convex basis library easily captures the general geometry of *Ψ*, its difficulty in perfectly reproducing the fine structure of the most complex experimental cases reveals genuine, highly coupled complexity in the underlying rock rheology. Future work will expand the SINP basis library to include physically motivated functional forms from continuum damage mechanics (e.g. specific strain-damage cross-coupling terms) alongside automated model selection criteria. Ultimately, the combination of TIP-INN discovery followed by SINP distillation creates a powerful two-stage pipeline: first, the neural network explores the full nonlinear constitutive state space without rigid model-form assumptions; second, SINP identifies the sparsest analytical expression consistent with the learned physics. This pipeline provides a resolution to the long-standing tension in geomechanics between phenomenological simplicity and physical completeness.

### Nonsmooth constitutive models

A central question for any continuous-time constitutive integrator is how it represents nonsmooth yielding, and the Prandtl elastoplastic and Perzyna viscoplastic benchmarks (Section S12) let us examine this directly. By replacing classical discrete return-mapping algorithms with a continuous neural integrator, TIP-INN approximates nonsmooth evolution laws, such as the sharp yield surfaces of rate-independent plasticity, with smooth continuous functions, naturally smoothing the sharp transition in the Helmholtz free energy as the material passes from the elastic to the plastic regime. The Perzyna model relaxes rate-independent plasticity through a finite viscosity, so that the stress may lie outside the static yield surface by an amount set by the plastic strain rate, and this rate dependence introduces a mathematical smoothness that the neural ODE integrator captures naturally. As a result, the predicted free energy and internal plastic-strain variables are more accurate for the Perzyna viscoplastic case than for the rate-independent elastoplastic case, where the nondifferentiable corner at yield remains the hardest feature to reproduce. This distinction persists under richer training data: adding eight diverse loading paths sharpens the viscoplastic free energy noticeably, whereas the rate-independent elastoplastic case does not benefit, which indicates that the nonsmooth yield corner is an intrinsic limitation of the continuous integrator rather than a data-limited one (Section S12). Remarkably, the network discovered a novel, nonlinear ISV formulation that accurately represents the macroscopic stress–strain elastoplastic behavior, further illustrating the structural nonuniqueness of macroscopic rheology. In this context, our neural integrator effectively acts as a viscoplastic regularization of rate-independent plasticity. While introducing a Newton-based discrete solver to TIP-INN could provide more precise representations of sharp yield surfaces (albeit at a significantly higher computational cost), this continuous smoothing is actually a physical advantage for geoscience. As noted in the introduction, the brittle-to-ductile transition in real geological materials-especially when averaged over a macroscopic representative elementary volume (REV)-is not a single, sharp mathematical discontinuity. It is a smooth, distributed transition, well represented by the continuous nature of our Neural ODE approach. However, we note that as macroscopic failure approaches, brittle damage often localizes into discrete shear bands or fracture planes, at which point a REV must be sufficiently large relative to these localized structures to define a meaningful homogenized behavior. Capturing this discrete strain localization remains a fundamental challenge for all continuum-based solvers, including TIP-INN.

### Gibbs free energy and other thermodynamic potentials

While the current framework targets predicting *Ψ* and $\Phi^{*}$ to solve strain-controlled boundaries, its architecture extends naturally to other thermodynamic potentials, such as the Gibbs free energy and internal energy. The Gibbs free energy switches the control variable from strain to stress via a Legendre transformation of *Ψ* (Eq. 13). By deploying the same convex neural network architecture to predict the Gibbs free energy, TIP-INN can easily model stress-controlled experiments. As demonstrated in Fig. S12, the network accurately predicts the Gibbs free-energy geometry for the Maxwell model, confirming that any thermodynamic potential that requires strict convexity and positivity can be discovered using this unified computational framework.

### Autonomous discovery

The three meta-questions raised by this work—(i) identifying optimal loading protocols to map potential geometry, (ii) physically justifying the number of ISVs, and (iii) mapping specific acoustic features to individual ISVs—naturally point toward an active-learning paradigm. Recent multiagent scientific discovery frameworks, such as ScienceClaw × Infinite (44), provide a compelling template for resolving these questions autonomously. In a prospective closed-loop extension, TIP-INN would serve as the core inference engine, continuously updating posteriors over the thermodynamic potentials (*Ψ* and $\Phi^{*}$) and ISV capacities. Simultaneously, overarching multiagent algorithms would dynamically rank candidate ancillary observables and select subsequent loading protocols to maximize information gain. As a preliminary step toward automating optimal protocol selection, we explored a stochastic Ornstein–Uhlenbeck (OU) loading process (detailed in Section S5). We found that under severely data-limited conditions, the continuous stochastic excitation of the OU process serves as an optimal experimental design, yielding significantly more robust predictions of the true free energy than standard cyclic or monotonically oscillatory loads. This advantage is specific to the data-limited regime and depends in part on the material model itself: once many loading paths of the same type are available, standard cyclic loading ultimately gives the most accurate potentials, and the relative ranking of protocols shifts with the constitutive form being learned, so the OU protocol is best read as an information-optimal choice for the first and most expensive experiments rather than a universally superior load (Section S5). More work is needed to characterize this dependence across model types, but the analysis and the learned potentials together already provide a path toward designing optimal loading protocols for training a first model and then refining it iteratively as data accumulate. In principle, an autonomous, active-learning loop could make these kinds of protocol-selection decisions automatically, and our OU result provides a concrete, physically grounded basis for such a framework.

However, the immediate practical bottleneck to realizing a fully autonomous, closed-loop approach in rock mechanics for Earth materials is the limitations of experimental apparatus. Current research-grade rock mechanics experiments (e.g. standard servo-controlled triaxial rigs) are designed around campaign-style operation: a fixed protocol is programmed manually, executed, and analyzed offline. They generally lack the standardized programmatic control APIs, low-latency continuous seismo-acoustic data streaming, and robust automated workflows required to feed a real-time autonomous loop that can cycle through hundreds of dynamically requested protocols. Consequently, closing this loop necessitates a significant co-development of laboratory instrumentation. Interestingly, because field-scale distributed acoustic sensing (DAS) networks natively support continuous, real-time data streaming, the first fully autonomous, active-learning applications of TIP-INN may practically emerge at the field scale before they are realized in the laboratory, an inversion of the standard technological development pathway.

### Broader implications for geoscience applications

Because it operates entirely within the general GSM framework, TIP-INN applies to a broad class of dissipative constitutive problems expressible in terms of convex thermodynamic potentials, including viscoelasticity, distributed damage, and viscoplasticity, as well as coupled thermo-mechanical deformation. As shown for the elastoplastic and viscoplastic benchmarks (Section S12), smooth and rate-dependent responses are captured accurately, whereas sharp rate-independent yield surfaces are represented only approximately by the continuous integrator. The explicit mathematical link between thermodynamic driving forces ($X^{d}$) and seismo-acoustic observables established here provides a consistent bridge from laboratory continuum mechanics directly to field-scale seismological monitoring. As observational geophysics increasingly deploys dense sensor networks (e.g. fiber optic based distributed acoustic sensing, large-N nodal arrays) that continuously record microseismicity and velocity variations, TIP-INN-derived constitutive laws-constrained by these multimodal field data-offer a highly robust pathway for upscaling physically interpretable, thermodynamically consistent constitutive models from the laboratory to the crustal scale. Because TIP-INN strictly enforces thermodynamic admissibility rather than merely interpolating data, it provides a mathematically rigorous foundation for the most difficult challenge in geodynamics: extrapolating laboratory laws across the nine-order-of-magnitude gap in strain rates required to model geological time scales.

### Limitations and future directions

We note that the current framework has limitations that invite future development. First, TIP-INN currently does not incorporate formal uncertainty quantification (UQ); the predicted potentials are deterministic point estimates. Future Bayesian extensions—such as deep ensembles over convex neural networks or Monte Carlo dropout applied to the neural integrator—would provide posterior distributions over the learned potentials and successfully propagate epistemic uncertainty to the predicted ISV trajectories (45).

Towards full thermodynamic applicability, incorporating temperature (*T*) as an additional state variable is structurally straightforward within the GSM formalism and requires only temperature-controlled experimental datasets for validation. Similarly, poro-mechanical coupling-where pore fluid pressure modifies the effective stress tensor and couples to damage-driven permeability evolution (46)-represents a critical extension for modeling fluid-saturated crustal rocks, fault zone hydrology, and reservoir geomechanics.

In the future, the present 1D uniaxial formulation can be extended to full 3D tensorial constitutive discovery to address field-scale geomechanical problems. This extension is architecturally tractable within the TIP-INN framework, requiring careful enforcement of frame indifference and material symmetry constraints on the convex neural network inputs to account for both inherent structural anisotropy (e.g. foliations) and damage-induced anisotropy. However, the practical utility of this extension hinges critically on the availability of concurrent multiaxial stress–strain data (47, 48). Using multiaxial loading platforms capable of simultaneously resolving independent stress and strain components is therefore a prerequisite for the 3D extension of TIP-INN to realistic geomechanical settings.

## Conclusion

We introduced the TIP-INN, a thermodynamically consistent neural network framework for constitutive discovery in geophysics. The network is trained exclusively on macroscopic stress–strain data pairs, to discover underlying Helmholtz free energy (*Ψ*) and dual dissipation ($\Phi^{*}$) potentials, which are known for our forward model data, but not for experimental data. With limited data, TIP-INN successfully reconstructs the geometry of *Ψ* and $\Phi^{*}$ simply by enforcing the non-negative mechanical dissipation and constitutive ODEs derived from these potentials within the Generalized Standard Materials formalism through architectural construction rather than soft penalties. This ensures physical consistency across elastic, plastic, and viscous regimes, eliminating the possibility of thermodynamic violations through the constitutive laws. To resolve the ill-posed inversion of unobservable internal state variables, TIP-INN directly incorporates laboratory seismo-acoustic data into the objective function, thereby explicitly linking subgrid-scale defect dynamics to macroscopic continuum mechanics. Subsequently, the SINP procedure distills the trained high-dimensional neural potentials into closed-form analytical equations to recover explicit, interpretable constitutive laws. By providing a flexible, data-driven, and physically rigorous framework, TIP-INN presents a new path for bridging disciplinary gaps across geophysics and geodynamics.

## Materials and methods

### The generalized standard materials formalism

In many geophysical problems, the infinitesimal strain approximation is a valid approach and significantly simplifies the computational implementation (10, 49, 50). In the GSM, the states are uniquely defined by the infinitesimal total strain, ε, and internal state variables, α. Additional thermodynamic state variables, such as temperature, *T*, and fluid pressure, $p_{f}$, can be incorporated for more complex systems (51, 52). In general, ε is an observable physical quantity in lab experiments or field data. In contrast, α, which encompasses irreversible phenomena such as damage, fracture density, and visco-plastic strain, cannot be observed directly. In some cases, seismo-acoustic data or proxies can help constrain the evolution of internal structure. To ensure thermodynamic consistency in GSM, the material response (constitutive model) of the system is represented through a set of two scalar thermodynamic potentials with respect to the state variables: Helmholtz free energy, *Ψ*, and dissipation potential, *Φ* (15, 53–58), which are extension of the linear Onsager thermodynamics framework (59–63). The key properties of the two scalar potentials are:

1. Helmholtz Free Energy, $\Psi (\varepsilon_{ij},\alpha_{k},T)$ : Defines the *storage* of energy (reversible) and can be defined as per unit volume (energy density—J/m${\;}^{3}$) or per unit mass (specific energy—J/kg). We use the per unit volume notation for the rest of the text. In general, the Helmholtz free energy, *Ψ*, of a system is determined by the total strains, $\varepsilon_{ij}$, internal state variables, $\alpha_{k}$, and temperature, *T*. For problems under isothermal conditions, *Ψ*, only associated to $\varepsilon =\varepsilon_{ij}$ and $\alpha =\alpha_{k}$, is smooth, non-negative and convex with respect to ε and α and Ψ=0 at the origin, where ε=0 and α=0.
  (1a ) $$\Psi (\varepsilon ,\alpha )\geq 0,\;\frac{\partial^{2}\Psi (\varepsilon ,\alpha )}{\partial \varepsilon^{2}}\geq 0,\;\frac{\partial^{2}\Psi (\varepsilon ,\alpha )}{\partial \alpha^{2}}\geq 0,$$
  (1b ) $$\Psi (0,0)=0,\;\frac{\partial \Psi (0,0)}{\partial \varepsilon }=0,\;\frac{\partial \Psi (0,0)}{\partial \alpha }=0,$$
2. Dissipation pseudo potential, $\Phi ({\dot{\varepsilon }}_{ij};{\dot{\alpha }}_{k},q;\alpha_{k},T)$: Defines the *rate* of entropy production (irreversible) and can be defined as per unit volume (Watts per cubic meter, $\text{W}/{\text{m}}^{3}$) or per unit mass (Watts per kilogram, W/kg). We use the per unit volume notation for the rest of the text. The dissipation potential *Φ* is determined by the rates of total strain, ${\dot{\varepsilon }}_{ij}$, rates of internal state variables, ${\dot{\alpha }}_{k}$, heat flux, q, internal state variables, $\alpha_{k}$, and temperature, *T*. Analogously, for isothermal problems, *Φ*, only associated with $\dot{\varepsilon }$ and $\dot{\alpha }$, is non-negative and convex with respect to $\dot{\varepsilon }$ and $\dot{\alpha }$ and Φ=0 at the origin, where $\dot{\varepsilon }=0$ and $\dot{\alpha }=0$, namely,
  (2a ) $$\Phi (\dot{\varepsilon },\dot{\alpha })\geq 0,\;\frac{\partial^{2}\Phi (\dot{\varepsilon },\dot{\alpha })}{\partial {\dot{\varepsilon }}^{2}}\geq 0,\;\frac{\partial^{2}\Phi (\dot{\varepsilon },\dot{\alpha })}{\partial {\dot{\alpha }}^{2}}\geq 0,$$
  (2b ) $$\Phi (0,0)=0,\;\frac{\partial \Phi (0,0)}{\partial \dot{\varepsilon }}=0,\;\frac{\partial \Phi (0,0)}{\partial \dot{\alpha }}=0.$$
  Compared with the Helmholtz free energy, the dissipation potential could be locally linear and nonsmooth, for example, in rate-independent elastoplasticity (64). These constraints on *Ψ* and *Φ* ensure that the general response of a standard material is always stable and thermodynamically consistent.

Within the GSM framework, the convexity requirements for the dissipation pseudopotential *Φ* and the Helmholtz free energy *Ψ* stem from fundamentally different physical principles. The convexity of *Φ* with respect to the flux rates is mandatory. Halphen and Nguyen (15) established that requiring *Φ* to be a convex, non-negative function that evaluates to zero at the origin strictly guarantees non-negative mechanical dissipation, provided the thermodynamic forces belong to the subdifferential of *Φ*. Furthermore, in the context of dual potentials, the Legendre-Fenchel conjugate $\Phi^{*}$ (Eq. 12) is uniquely defined. This conjugate is always mathematically well-defined and inherently convex, eliminating any concerns regarding illegal Legendre transformations when mapping between flux and force spaces.

In contrast, the strict convexity of the Helmholtz free energy Ψ(ε,α) is a modeling choice rather than a mandatory thermodynamic requirement. A convex *Ψ* is sufficient to ensure material stability and the existence of unique boundary-value solutions, making it highly effective for modeling stable dissipative processes such as viscoelastoplasticity and distributed damage. However, enforcing convexity on *Ψ* inherently excludes phenomena driven by nonconvex energy landscapes, such as structural phase transitions, snap-back instability, or severe strain-softening leading to strong localization. Modeling such physical behaviors typically requires polyconvex, quasiconvex, or relaxed-envelope formulations (28, 65).

Having specified the thermodynamic potentials, the constitutive model must satisfy the 2nd law of thermodynamics. This requirement is expressed through the Clausius–Duhem Inequality (CDI), derived from the entropy and energy conservation equations, which restricts admissible constitutive relations and ensures non-negative mechanical dissipation (16, 66):


(3) $$D\;=\;\sigma \;:\;\dot{\varepsilon }\;-\;\rho (\dot{\Psi }+s\dot{T})\;-\;\frac{q\cdot \nabla T}{T}\;\geq 0,$$

where D is the dissipation rate, σ the stress, *ρ* the density, and *s* is the entropy. The time derivative of Ψ(ε,α,T) is:


(4) $$\dot{\Psi }\;=\;\frac{\partial \Psi }{\partial \varepsilon }\;:\;\dot{\varepsilon }\;+\;\frac{\partial \Psi }{\partial \alpha }\dot{\alpha }\;+\;\frac{\partial \Psi }{\partial T}\dot{T}.$$

Substituting the above equation into Eq. 3 and separating the heat flux term to isolate the mechanical dissipation, we obtain:


(5) $$\left(\sigma \;-\;\rho \frac{\partial \Psi }{\partial \varepsilon }\right)\;:\;\dot{\varepsilon }\;-\;\rho \left(s+\frac{\partial \Psi }{\partial T}\right)\dot{T}\;+\;\left(-\rho \frac{\partial \Psi }{\partial \alpha }\right)\dot{\alpha }\geq 0.$$

By the Coleman–Noll procedure (16), the coefficients of the arbitrary control rates ($\dot{\varepsilon },\dot{T}$) must vanish. Thus, the reversible/energetic stress, $\sigma^{r}$, and the internal/driving force, $A_{\alpha }$ (also denoted as $\tau_{\alpha }$ in some studies), are defined as:


(6) $$\sigma^{r}\;=\;\rho \frac{\partial \Psi }{\partial \varepsilon },\;A_{\alpha }\;=\;\rho \frac{\partial \Psi }{\partial \alpha },$$

respectively. Similarly, entropy is defined as $s=-\frac{1}{\rho }\frac{\partial \Psi }{\partial T}$. Consistent with the per-unit-volume convention for *Ψ* and *Φ* introduced in The generalized standard materials formalism section, we absorb *ρ* into these potentials from this point onward, so that subsequent expressions (e.g. Eq. 10) are written directly in terms of ∂Ψ/∂ε without an explicit density factor. To simplify the presentation, we use isothermal conditions in this study, though the proposed framework can be extended to nonisothermal conditions by introducing temperature as an additional state variable. Substituting Eq. 4 and Eq. 6 into Eq. 3, we obtain:


(7) $$D\;=\;(\sigma -\sigma^{r})\dot{\varepsilon }\;-\;A_{\alpha }\dot{\alpha }\;=\;\sigma^{d}\dot{\varepsilon }\;+\;X^{d}\dot{\alpha }\;\geq 0,$$

where the dissipative stress, $\sigma^{d}$, and dissipative driving force, $X^{d}$ (also denoted as $\tau^{d}$ in some studies (e.g. 35)), are derived from *Φ* as


(8) $$\sigma^{d}\;=\;\frac{\partial \Phi }{\partial \dot{\varepsilon }}\;=\;\sigma -\sigma^{r},\;X^{d}\;=\;\frac{\partial \Phi }{\partial \dot{\alpha }}\;=\;-A_{\alpha }.$$

Equation 8 describes the relations between irreversible phenomena and thermodynamic forces (Biot–Rayleigh equation). Here, $X^{d}$ is the *thermodynamic force* driving microstructural internal variable evolution and $A_{\alpha }$ is its conjugate and called the thermodynamic affinity. Overall, $X^{d}$ provides the direct link between kinetics and thermodynamics.

To contextualize these forces physically, consider a Maxwell model. In this framework, the internal state variables α represent the hidden internal viscous strains of the constituent dashpots. The reversible force, $A_{\alpha }$, represents the energetic restoring stress accumulated in the adjacent elastic springs resulting from that internal deformation. Conversely, the dissipative force, $X^{d}$, corresponds directly to the viscous drag stress generated within the dashpot itself, which resists the rate of internal deformation ($\dot{\alpha }$). The required thermodynamic equality $X^{d}=-A_{\alpha }$ fundamentally enforces internal equilibrium ensuring that the elastic restoring forces are perfectly balanced by the viscous resisting forces at all times.

With the definitions of Eq. 8, Eq. 7 is recast as:


(9) $$D\;=\;\frac{\partial \Phi }{\partial \dot{\varepsilon }}\dot{\varepsilon }\;+\;\frac{\partial \Phi }{\partial \dot{\alpha }}\dot{\alpha }\;\geq 0.$$

For nondissipative material models (D=0), for example, elasticity, we have $\sigma^{d}\;=\;0$ and $X^{d}\;=\;0$ and the entire constitutive behavior can be described solely by the state laws associated to *Ψ* (Eq. 6). Substituting Eq. 6 into Eq. 8, the constitutive relations can be expressed in terms of *Ψ* and *Φ* as:


(10) $$\sigma \;=\;\frac{\partial \Psi }{\partial \varepsilon }+\frac{\partial \Phi }{\partial \dot{\varepsilon }},\;0\;=\;\frac{\partial \Psi }{\partial \alpha }+\frac{\partial \Phi }{\partial \dot{\alpha }}.$$

For solid materials, it is commonly assumed that the dissipation potential does not depend on the strain rate (except for a single Kelvin model (64)). Then the constitutive relations described by Eq. 10 can be further simplified to


(11) $$\sigma =\frac{\partial \Psi }{\partial \varepsilon },\;0=\frac{\partial \Psi }{\partial \alpha }+\frac{\partial \Phi }{\partial \dot{\alpha }}.$$

By applying a Legendre–Fenchel transformation to the dissipation potential *Φ*, we obtain a dual dissipation potential, $\Phi^{*}$ (15, 57) as


(12) $$\Phi^{*}(X^{d})\;=\;\underset{\dot{\alpha }}{\sup}\left(X^{d}\cdot \dot{\alpha }-\Phi (\dot{\alpha })\right),\;\text{with }\dot{\alpha }\;=\;\frac{\partial \Phi^{*}}{\partial X^{d}}.$$

The above equation describes an evolution process for the internal variable and is thus often more useful than the state laws of Eq. 8. Similar to the properties of *Φ*, $\Phi^{*}$ is non-negative and convex due to the Legendre-Fenchel transformation property. Notice that Eq. 9 (2nd law of thermodynamics) is automatically satisfied if *Φ* and $\Phi^{*}$ are convex, non-negative, and zero at the origin.

Although our primary analysis in this study uses the Helmholtz free energy and dual dissipation, it is straightforward to use the GSM theory with other thermodynamic potentials, such as the Gibbs Free Energy, Internal Energy, and Enthalpy. Typically, *Ψ* serves as the central pivot for these transformations since it naturally couples the thermal intensive variable (*T*) with the mechanical extensive variable (ε), which are both lab/field measurable quantities. For instance, we can switch control variables from ε (strain-controlled) to σ (stress-controlled), using the Legendre transformation to obtain the Gibbs free energy (*G*) of the system:


(13) $$G(\sigma ,\alpha ,T)=\underset{\epsilon }{\sup}\left(\Psi -\sigma \;:\;\varepsilon \right).$$

Analogously, the strains and driving forces can be derived from *G* as ε=∂G/∂σ and $X^{d}=\partial G/\partial \alpha$, respectively.

The key advantage of working directly with thermodynamic potentials, instead of stress-strain relationships is that the material response of a generalized solid relies heavily on the local curvature of the thermodynamic potentials. For instance, the Hessian of *Ψ*, $L\;=\;\partial^{2}\Psi (M)/\partial M^{2}$ (where M refers to the relevant state variables), encodes the fundamental reversible stiffness properties of the material (67–69). Specifically, the second-order derivative of *Ψ* with respect to strain, $C\;=\;\partial^{2}\Psi /\partial \varepsilon^{2}$, gives the isothermal elastic moduli. At present, a key challenge with using GSM for constitutive modeling with geophysical applications lies in learning the appropriate forms of the potentials (*Ψ* and $\Phi^{*}$) from lab and field datasets, which can then be used for continuum models. Machine learning algorithms are now widely used for constitutive modeling due to their computational efficiency and adaptability for nonlinear models (22, 70–76). This strongly motivates our approach, outlined in the next section, to develop a neural network framework for learning the GSM potentials directly from the strain–stress time series data.

### Thermodynamics of Irreversible Processes Informed Neural Network

In many relevant experiments for geological materials, the rates of state variables and the full state of materials are not known at each time step. Thus, the time-evolution process for state variables is inherently nonintegrable with classical numerical methods without assuming a constitutive model and/or thermodynamic potentials. In contrast, a neural integrator (using a neural network learned integration function) only requires partial and sparse material states for time evolution since the state variables can be predicted by minimizing the error between the predicted trajectories and the experimental measurements. Therefore, the neural integrator generally solves the evolution equations more efficiently than an implicit numerical method (i.e. Newton method). The idea of embedding the neural integrator for state variables’ evolution has been investigated for constitutive modeling in many different works (25, 32, 35, 77–81). These works focus primarily on the stress–strain relationship, with limited attention to the predicted thermodynamic potentials (e.g. Helmholtz free energy and dual dissipation).

Compared to the numerical ODE solver, the neural integrator allows the parametrization of continuous-time dynamics of state variables:


(14) $$\dot{y}\;=\;f_{\theta }(y(t),p),\;y(0)\;=\;y^{0},\;t\in [0,t_{e}],$$

where p is the set of control variables that the state variables depend on and $f_{\theta }$ is the evolution equation (ODE) parametrized by a feed-forward neural network with *θ* representing the weights and bias terms. Considering an initial condition $y^{0}$ at t=0 and integrating Eq. 14 from 0 to any time *t* yields the state variable y(t) at *t*


(15) $$y_{\theta }(t)\;=\;y^{0}+\int_{0}^{t}f_{\theta }(y(\tau ),p)\text{d}\tau .$$

During the learning process, *θ* is determined by a gradient descent algorithm to minimize the loss function


(16) $$L_{I}=\frac{1}{N}\sum_{n=1}^{N}{\left(y_{\theta }(n)-y(n)\right)}^{2},$$

where we sum the errors between the neural-network predictions, $y_{\theta }(n)$, and the experimental measurements, y(n), at a series of discrete time points $t_{n}$ with n=1,2,…,N and $t_{N}=t_{e}$.

We propose a general parametric neural network (TIP-INN) for constitutive modeling by learning the thermodynamic potentials of Helmholtz free energy, *Ψ* and dual dissipation, $\Phi^{*}$, modified after the neural integrator in (25, 35). Figure 1 illustrates the detailed structures of TIP-INN, which involves a neural integrator to solve the evolution equation and convex neural networks to predict *Ψ* and $\Phi^{*}$. In particular, we use a modular forward solver based on the GSM framework that derives system dynamics directly from fundamental thermodynamic potentials, bypassing the need to manually derive and integrate specific constitutive ODEs for each new rheology. In this architecture, we have the definitions for the scalar *Ψ* and $\Phi^{*}$. At each time step, an automatic differentiation engine evaluates the gradients of these potentials with respect to the state variables (∇Ψ, $\nabla \Phi^{*}$). This operation dynamically computes the thermodynamic driving forces, such as reversible stresses and thermodynamic affinities, as well as the evolution rates of internal state variables (e.g. viscous strains and damage parameters). The resulting auto-derived equations are then integrated using a stiff ODE solver with adaptive time-stepping. The primary advantage of this potential-driven approach is extreme structural flexibility; transitioning between disparate material behaviors, ranging from simple thermoelasticity to complex thermo-elasto-viscous models with nonlinear flow laws requires modifying only the input scalar potentials, leaving the core solver mechanics completely unchanged. Furthermore, the framework naturally accommodates both strain and stress-controlled boundary conditions (employing local Legendre transforms at each time step for the stress-forced case) and rigorously tracks fully coupled temperature evolution by incorporating mechanical dissipation and specific heat capacity derived from the Hessian of the free energy.

To enforce the convex and positive properties of *Ψ* and $\Phi^{*}$ for constitutive modeling, we follow the concept of convex neural network with skip connections proposed by Amos et al. (23). By constraining the network’s weight parameters to be non-negative and choosing convex, nondecreasing activation functions, the network’s output is guaranteed to be convex with respect to the input. With such a strategy, the convexity of *Ψ* with respect to ϵ and α is preserved, as well as $\Phi^{*}$ with respect to $X^{d}$. Figure 1 shows the detailed structures of a convex neural network. Choosing convex activation functions is straightforward. Specifically, we adopt a quadratic activation function to model viscoelastic materials (Maxwell and Burgers). The smoothed absolute activation function is chosen to model the experimental data with fracture propagation as sharp transitions are observed on the stress-strain relationships. The enforcement of non-negative weighting parameters in the network is achieved by setting $w_{i}^{+}=g(w_{i})$ where $g\;:\;R\mapsto R^{+}$ is an auxiliary function that maps real numbers to non-negative real numbers. The convex neural networks for both *Ψ* and $\Phi^{*}$ have very similar structures, except for the different inputs (see Fig. 1).

Given the data regime and the framework depicted in Fig. 1, the learning process of TIP-INN consists of minimizing the following loss function


(17) $$\begin{array}{rl} L_{\mathrm{TIP}-\mathrm{INN}} & =\;L_{\sigma }\;+\;L_{D}\;+\;L_{\Psi_{+}}\;+\;L_{\Phi_{+}^{*}}\\ & \;+\;L_{\sigma (0)}\;+\;L_{\Psi (0)}\;+\;L_{\Phi^{*}(0)}\;+\;L_{L_{2}}. \end{array}$$

$L_{\sigma }$ is the loss on the stress; $L_{D}$, $L_{\Psi_{+}}$ and $L_{\Phi_{+}^{*}}$ fulfill the positivity of the intrinsic dissipation rate, Helmholtz free energy and dual dissipation, respectively. $L_{\sigma (0)}$, $L_{\Psi (0)}$ and $L_{\Phi^{*}(0)}$ enforce the initial conditions for the stress, Helmholtz free energy and dual dissipation, respectively. To avoid data overfitting, an $L_{2}$-regularization on the weighting parameters ($L_{L_{2}}$) with a small penalty parameter $10^{-5}$ is added to the loss function. Note that different weighting parameters could be considered to control the importance of different terms in Eq. 17 although equal weights are used in this work. The specific definitions of each term are as follows:


$$\begin{array}{rl} L_{\sigma } & =\frac{1}{N}\sum_{n=1}^{N}{\left(\sigma_{\theta }(n)-\sigma (n)\right)}^{2},\;L_{D}=\frac{1}{N}\sum_{n=1}^{N}\left\langle -D_{\theta }(n)\right\rangle ,\\ L_{\Psi_{+}} & =\frac{1}{N}\sum_{n=1}^{N}\left\langle -\Psi_{\theta }(n)\right\rangle ,\;L_{\Phi_{+}^{*}}=\frac{1}{N}\sum_{n=1}^{N}\left\langle -\Phi_{\theta }^{*}(n)\right\rangle ,\\ L_{\sigma (0)} & ={\left(\sigma_{\theta }(n=1;t=0)\right)}^{2},\;L_{\Psi (0)}={\left(\Psi_{\theta }(n=1;t=0)\right)}^{2},\\ L_{\Phi^{*}(0)} & ={\left(\Phi_{\theta }^{*}(n=1;t=0)\right)}^{2},\;L_{L_{2}}=\frac{1}{N_{w}}\sum_{n=1}^{N_{w}}{\left(w_{i}\right)}^{2}, \end{array}$$

where ⟨x⟩=(|x|+x)/2 is the Macaulay bracket and $N_{w}$ denotes the number of weighting parameters in the neural network. Note that the sampling frequencies for each term in the loss function (Eq. 17) can be adjusted by the user to reduce the computational cost of training. For example, adding more sample points for the evolution of state variables could improve prediction accuracy. In this work, the full data points are considered in $L_{\sigma }$, $L_{D}$, $L_{\Psi_{+}}$, and $L_{\Phi_{+}^{*}}$. An early stopping criterion during training is used to avoid overfitting. Additional training details and hyperparameter settings are provided in Section S2.

A key difference in our approach compared to existing variational methods (e.g. the GSMN of (25)) is the use of a neural integrator rather than local optimization to evolve internal variables ($\dot{\alpha }$). In the incremental variational formulation, the ODE is not solved directly; instead, a local minimization problem is solved at every time step $t_{n+1}$:


$$\begin{array}{r} \left(\epsilon_{n+1},\alpha_{n+1})=\underset{\epsilon ,\alpha }{\arg\;\min}(\Psi (\epsilon ,\alpha \right)\\ +\Delta t\Phi^{*}\left(\frac{\alpha -\alpha_{n}}{\Delta t}\right)-\sigma \;:\;\epsilon ). \end{array}$$

Replacing the numerical solver (or Newton–Raphson iteration) with a neural integrator, the training process is significantly faster and more robust as long as the time steps are sufficiently small, since the time evolution reduces to a single forward pass.

### Sparse identification of nonlinear potentials

The objective of this framework is to extract a strictly convex, analytically exact free energy functional V(ϵ,α) from discrete state-space data. Here, $\epsilon \in R^{d_{\epsilon }}$ represents observable kinematic states (e.g. strain), and $\alpha \in R_{d_{\alpha }}^{+}$ denotes internal latent state variables mapping irreversible dissipation (e.g. damage, defect density).

Standard sparse identification architectures, such as the sparse identification of nonlinear dynamics (SINDy) (39), predominantly utilize unconstrained sequentially thresholded least squares (STLSQ) or $L_{1}$-regularized regression (Lasso) (82). These algorithms are structurally deficient for thermodynamic potential identification for two primary reasons: $L_{1}$ penalties introduce amplitude shrinkage (82, 83) that violates exact energy equivalence, and unconstrained algorithms suffer catastrophic collinear instability over highly resolved activation grids. To resolve this, we present an overcomplete, physics-informed basis projection coupled with a dual-manifold scaled non-negative least squares (NNLS) active-set solver (84).

#### The convex overcomplete basis and reference state

We map the phase space $x=[\epsilon^{T},\alpha^{T}{]}^{T}$ into a high-dimensional dictionary Θ(x) composed strictly of positive semidefinite basis functions. The functional is expressed as:


(19) $$V(x)=\sum_{k}\beta_{k}\theta_{k}(x),\;\text{subject to }\beta_{k}\geq 0\;\forall k.$$

By strictly enforcing 2p≥2 for all polynomial orders and omitting an intercept parameter ($\beta_{0}=0$), we ensure that $\theta_{k}(0)=0$. This rigorously satisfies the fundamental thermodynamic reference state V(0)=0; an undeformed, virgin material possesses zero stored energy.

The dictionary Θ isolates the system into distinct energetic sub-manifolds:

##### I. Isolated kinematics and internal storage

The pure elastic strain energy and isolated internal defect energy are mapped as:


(20) $$\theta_{kin}\in \left(\langle \epsilon_{i}\rangle_{+}^{2p},\langle -\epsilon_{i}\rangle_{+}^{2p}\right),\;\theta_{iso}\in \left(\alpha_{i}^{2p}\right)$$

Because the physical definitions of the internal variables restrict them to the positive orthant ($\alpha_{i}\geq 0$), the computationally expensive Macaulay bracket $\langle \cdot \rangle_{+}$ is mathematically redundant for $\theta_{iso}$ and is bypassed to accelerate tensor operations.

##### II. Coupled dissipative manifolds

The interaction between kinematics and the internal state is governed by discrete activation thresholds. These define the exact yield surfaces where the kinematic driving force exceeds the resistance of the internal defect network:


(21) $$\theta_{cpl}\in \left(\langle \pm \epsilon_{i}-c_{m}\alpha_{j}\rangle_{+}^{2p}\right),$$

where $c_{m}\in C_{\mathrm{grid}}$ operates on a highly resolved, overcomplete discrete grid (e.g. [0.1,3.0]). The Macaulay bracket ensures $C^{0}$ continuity across the phase boundary, maintaining a dormant state until the precise local driving force threshold is breached.

#### Latent interaction structure: smooth vs. piecewise

Interactions between competing internal mechanisms (e.g. distinct dislocation slip systems) require specific structural assumptions based on the underlying physics. We define two distinct bases to map the latent submanifold:

1. Bracketed (piecewise directional): Assumes strict, asymmetric thresholds where one mechanism permanently pins or dominates another. Mapped via $\langle \alpha_{i}-c_{m}\alpha_{j}\rangle_{+}^{2p}$, energy is exclusively stored if $\alpha_{i}>c_{m}\alpha_{j}$. This generates a $C^{0}$ continuous, piecewise phase space representing definitive microstructural barriers.
2. Unbracketed (smooth symmetric): Assumes continuous, mutual energy exchange without discrete activation barriers. Mapped via symmetric polynomials $(\alpha_{i}\pm \alpha_{j}{)}^{2p}$. Because the exponent 2p is inherently even, the manifold remains globally convex and $C^{\infty }$ continuous. This construction maps smooth, symmetric restorative thermodynamic forces between coupled latent fields. We also include some additional nonlinear terms like $(\alpha_{i}*\alpha_{j}{)}^{2p}$. in the basis function.

#### Exact sparse recovery via active-set NNLS

To identify the sparse set of active thermodynamic mechanisms, standard $L_{1}$ optimization solves $\min\|\Theta \beta -y{\|}_{2}^{2}+\lambda \|\beta {\|}_{1}$ (82). However, this introduces an unphysical shrinkage bias ($\beta_{L1}=|\beta_{\mathrm{true}}|-\lambda$) (83) into the identified stiffness parameters, thereby destroying exact energy conservation. Furthermore, unconstrained regression on the highly collinear grid $C_{\mathrm{grid}}$ results in massive, oscillating positive and negative coefficients as the solver attempts to cancel localized noise.

Thus, we do not use algebraic penalty regularizers in our analysis and instead reframe the identification as a constrained convex optimization problem using NNLS. To stabilize the active-set selection against the extreme ill-conditioning inherent in the overcomplete grid $C_{\mathrm{grid}}$, we perform a bijective mapping of the dictionary onto an $L_{\infty }$ unit hypercube. We define the diagonal scaling matrix $S\in R^{K\times K}$ where $S_{kk}=\|\theta_{k}{\|}_{\infty }$, and a target scale $s_{y}=\|y{\|}_{\infty }$. We then solve the dimensionless optimization:


(22) $$\min_{\hat{\beta }}\frac{1}{2}{\left\|(\Theta S^{-1})\hat{\beta }-s_{y}^{-1}y\right\|}_{2}^{2}\;\text{subject to}\;{\hat{\beta }}_{k}\geq 0\;\forall k,$$

where $\hat{\beta }$ is the scaled parameter vector. This condition ensures that every basis function $\theta_{k}$ contributes with O(1) weight to the initial gradient calculation, preventing the solver from prematurely pinning low-amplitude but physically correct mechanisms to the zero-boundary.

The strict non-negativity constraint forms a geometric boundary that fundamentally neutralizes collinear instability; the solver is mathematically forbidden from utilizing negative amplitudes to cancel redundant grid terms. The success of this approach is dictated by the Karush–Kuhn–Tucker (KKT) complementary condition (85) at the optimal solution ${\hat{\beta }}^{*}$:


(23) $${\hat{\beta }}_{k}^{*}\cdot g_{k}^{*}=0\;\forall k,$$

where $g_{k}^{*}$ is the objective gradient. This condition mandates a strict bifurcation of the overcomplete dictionary. If a feature is inactive (spurious or collinear), it is pinned to the boundary (${\hat{\beta }}_{k}^{*}=0$) with a positive gradient pointing outward. If a feature is active (${\hat{\beta }}_{k}^{*}>0$), its local gradient must be exactly zero ($g_{k}^{*}=0$).

Once the optimal scaled vector ${\hat{\beta }}^{*}$ is identified via the KKT-constrained active-set projection, the exact physical stiffness parameters are recovered via the inverse mapping:


(24) $$\beta^{*}=s_{y}S^{-1}{\hat{\beta }}^{*}.$$

Because this scaling is a purely linear transformation and avoids objective-function penalties (e.g. $\lambda \|\beta {\|}_{1}$), it preserves the exact unbiased local minimum. This “Dual-Manifold” approach combines the numerical stability of bounded optimization with the theoretical exactitude of ordinary least squares strictly on the recovered physical support manifold. A standard numerical threshold filter is applied postrecovery to eliminate machine-precision floating-point artifacts, yielding exact $L_{0}$ sparsity.

Since the gradient is precisely zero for all active terms, the NNLS active-set algorithm operates equivalent to unconstrained Ordinary Least Squares strictly on the true physical support manifold. This isolates the $L_{0}$ unpenalized minimum, recovering the exact theoretical amplitude (e.g. retrieving a stiffness of exactly 250.0) while enforcing global convexity and unconditional thermodynamic stability. The SINP basis library employed here captures the general geometry of the learned potentials but sometimes struggles with complex cross-coupling terms in the most nonlinear cases; future work will expand the library to include physically motivated functional forms from continuum damage mechanics (e.g. $(1-\alpha {)}^{n}\varepsilon^{2}$ coupling terms) (86–88).

## Supplementary Material

pgag349_Supplementary_Data

## Data Availability

The data underlying this article are available in Zenodo Repository and can be accessed with DOI: https://doi.org/10.5281/zenodo.19195925.
