# Supplementary material for: TIP-INN: A thermodynamics-informed machine learning framework for the mechanical behavior of rocks
Source: PNAS Nexus. 2026 Oct 9;5(11):pgag349. doi: 10.1093/pnasnexus/pgag349 (PMC13721771; doi:10.1093/pnasnexus/pgag349)
Supplement: Supplementary file 1 [file pgag349_supplementary_data.pdf]

PNAS Nexus, 2026, pp. 1–19

doi:

Supporting Information

SUPPORTING INFORMATION

Supporting Information for: TIP-INN: A Thermodynamics-informed Machine Learning Framework for the Mechanical Behavior of Rocks

Kangan Li,[a] Tushar Mittal*,[a] Benjamin Holtzman,[b,c] Seth Saltiel,[d] Raphael Affinito[a,e] and Loic Daridon[f]

[a]Department of Geosciences, The Pennsylvania State University, University Park, Pennsylvania, United States of America, 16802, [b]Lamont Doherty Earth Observatory, Columbia University, Palisades, New York, United States of America, 10964, [c]Earth Resources Laboratory, Massachusetts Institute of Technology, Cambridge, MA, United States of America, 02139, [d]Department of Earth and Atmospheric Sciences, Cornell University, Ithaca, New York, United States of America, 14850, [e]GFZ Helmholtz Center for Geosciences, Telegrafenberg, 14473 Potsdam, Germany and [f]Laboratoire de Mécanique et Génie Civil, Université de Montpellier, France

Tushar Mittal: tmittal@psu.edu

Supplementary Material

Rheological representations

Phenomenologically, the overall mechanical behavior of any materials, in 1D, can be modeled by arbitrary serial and parallel arrangements of a few elementary (non)-linear constitutive model elements - elastic springs, viscous dashpots, and frictional sliders, representing the elastic, viscous, and plastic processes, respectively [31]. Figure S1 shows the rheological representations for Maxwell and Burgers viscoelastic models. The formulas of Helmholtz free energy for Maxwell and Burgers models are $\Psi = \frac{1}{2}E_v\boldsymbol{\epsilon}_e^2$ and $\Psi = \frac{1}{2}E_{v_1}\boldsymbol{\epsilon}_{e_1}^2 + \frac{1}{2}E_{v_2}\boldsymbol{\epsilon}_{e_2}^2$, respectively. The formulas of dual dissipation for Maxwell and Burgers models are $\Phi^* = \frac{1}{2\boldsymbol{\eta}_v}\boldsymbol{\sigma}_v^2$ and $\Phi^* = \frac{1}{2\boldsymbol{\eta}_{v_1}}\boldsymbol{\sigma}_{v_1}^2 + \frac{1}{2\boldsymbol{\eta}_{v_2}}\boldsymbol{\sigma}_{v_2}^2$, respectively.

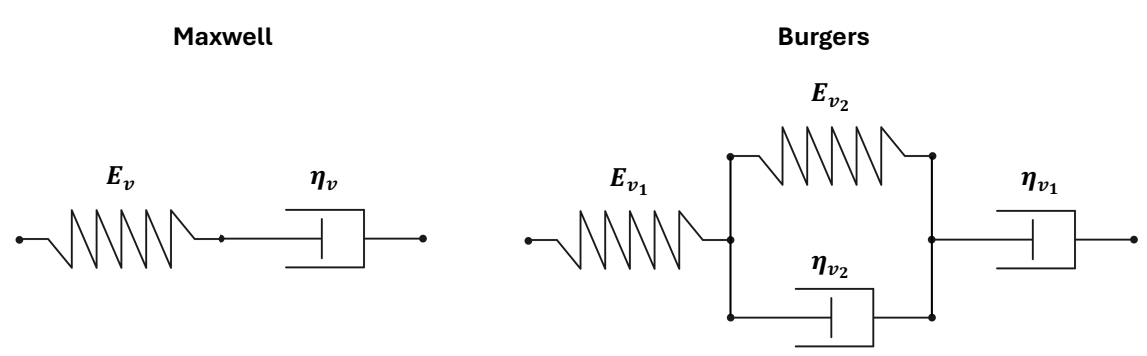

Fig. S1. Rheological representations of the Maxwell viscoelastic model with a single internal variable (associated with $\boldsymbol{\eta}_v$), and Burgers viscoelastic model with two internal variables (associated with $\boldsymbol{\eta}_{v_1}$ and $\boldsymbol{\eta}_{v_2}$).

TIP-INN training details

In all test cases, the learning rate was set to 10^{-3}, and the training was allowed to progress for 20,000 epochs with an early stopping criterion to avoid overfitting. In TIP-INN, the convex neural network architecture consists of an input layer with a linear activation function, followed by a single hidden layer. The hidden layer contains multiple fully connected neurons for the Maxwell and Burgers models, and 20 fully connected neurons for the experimental datasets, and is used to learn the Helmholtz free energy (Ψ) and dual dissipation Φ^*. The output layer with a linear activation function contains a single neuron, consistent with the scalar nature of Ψ and Φ^*.

As described in Section 6, a quadratic activation function is used for modeling Maxwell and Burgers viscoelastic materials, while a smoothed absolute activation function is employed for experimental data. Our numerical tests indicate that a single hidden layer with 10-30 neurons is generally sufficient to capture a wide range of constitutive behaviors from both analytical and experimental datasets. Increasing the hidden layer depth does not improve the accuracy of the predicted thermodynamic potentials. In fact, introducing multiple hidden layers (e.g., > 4) can lead to training instability, as the quadratic activation function amplifies weights multiplicatively across layers, resulting in divergence.

TIP-INN to model Maxwell viscoelastic materials

Figure S2 shows the predicted stress for different types of loads by training TIP-INN with a single load. Notice that accurate stresses were predicted in nearly all cases. Figure S3 presents the predicted stress and Helmholtz free energy obtained by TIP-INN on the training dataset for the Maxwell viscoelastic model. The results are consistent with those observed for the testing data discussed in Section 3.1, leading to similar conclusions regarding the accuracy and robustness of TIP-INN. In addition, Figure S3 shows that the training and testing

© The Authors. All rights reserved.

datasets exhibit distinct frequency characteristics, as revealed by the power spectral analysis. In this study, the Lomb-Scargle periodogram is employed to evaluate the power spectral density for frequencies below 10 Hz.

TIP-INN to model Burgers viscoelastic materials

Figure S4 shows the predicted viscosity and Young's modulus for TIP-INN on the testing data considering a Burgers viscoelastic model. Notice that Burgers model can be effectively approximated by the Maxwell model with a nonlinear Young's modulus (left column). Moreover, the same macroscopic stress-strain response for Burgers model can be achieved through different combinations of internal linear/nonlinear material properties. Thus, the predicted material properties are observed to be significantly different from the true solutions.

The cyclic and monotonically oscillatory loads used to train TIP-INN are plotted in Figure S5, along with the predicted stress, Helmholtz free energy and internal variables. The training results lead to conclusions consistent with those obtained from the testing data (see Section 3.2). In the case with two internal variables, TIP-INN predicts two nearly identical internal variables. Consequently, the corresponding predicted viscosities (Figure S4) are very similar, as are the associated internal stresses (Figure 3).

Loading Protocols and the Ornstein-Uhlenbeck Process

In classical model identification, it is well-established that the accuracy and generalization of a model heavily depend on the richness of the training data. Standard protocols often rely on cyclic loads or monotonically increasing loads with (or without) oscillations to excite a material's internal modes. However, when experimental data is severely limited (e.g., restricted to a single loading path), these standard loads may fail to adequately expose the underlying thermodynamic state variables. To address this, we explore the use of a stochastic Ornstein-Uhlenbeck (OU) process [2] combined with a monotonic ramp as an optimal experimental design strategy for training TIP-INN.

OU Process. The objective of an optimal loading protocol is to simultaneously drive the material into nonlinear, dissipative regimes while continuously exciting all dynamically independent modes. We achieve this by defining the applied strain $\varepsilon(t)$ as the sum of a constant drift and a mean-reverting stochastic noise process:

$$\varepsilon(t) = rt + \eta(t) \tag{S1}$$

where r is a constant ramp rate representing sustained monotonic drift. The stochastic component $\eta(t)$ is governed by the Ornstein-Uhlenbeck stochastic differential equation:

$$\mathrm{d}\eta = -\theta_{\mathrm{OU}}\eta\,\mathrm{d}t + \sigma_{\mathrm{diff}}\,\mathrm{d}W_t \tag{S2}$$

Here, θ_{OU} is the mean-reversion rate, σ_{diff} is the diffusion (noise) amplitude, and W_t represents a standard Wiener process (Brownian motion). The physical intuition behind this protocol is twofold: the monotonic ramp (rt) ensures a positive time-averaged strain rate, steadily accumulating dissipation and pushing the material into regimes where nonlinear mechanisms (such as damage or plasticity) govern the response. Simultaneously, the OU process (η) injects continuous, bounded frequency noise that probes the local parameter sensitivities of the internal state variables (ISVs) at every stage of deformation.

Why the OU process is well suited to potential discovery. Beyond this operational intuition, the Ornstein-Uhlenbeck process has structural properties that make it close to an ideal excitation for learning a thermodynamic potential from a single record. It is the continuous-time analogue of a discrete first-order autoregressive, AR(1), process, and by Doob's theorem it is the only nontrivial process that is simultaneously Gaussian, Markovian, and stationary. Because the drift term $-\theta_{\mathrm{OU}}\eta$ is mean-reverting rather than purely diffusive, the excitation stays bounded around its zero mean, with a stationary variance that saturates at $\sigma^2_{\mathrm{diff}}/(2\theta_{\mathrm{OU}})$ instead of growing without bound as it would for Brownian motion. This boundedness is what makes the OU process physically usable as a controlled loading path: it explores the strain and internal-variable state space broadly, yet it does not wander off to unphysical amplitudes. Its autocorrelation decays exponentially, $\rho(\tau) = e^{-\theta_{\mathrm{OU}}|\tau|}$, so the single parameter θ_{OU} sets the correlation time of the excitation and therefore its spectral content, with a Lorentzian power spectral density $S(\omega) \propto \sigma^2_{\mathrm{diff}}/(\theta^2_{\mathrm{OU}} + \omega^2)$. That broadband and tunable spectrum is the mechanistic reason the OU process acts as a near-optimal experimental design under data-limited conditions: a single OU path samples a wide and controllable band of strain rates and amplitudes, exciting the internal-variable dynamics more informatively than a narrowband cyclic or monotonic ramp and thereby constraining more of the curvature of the free-energy and dissipation potentials from one experiment.

To evaluate the efficacy of the OU process against standard protocols, we trained TIP-INN using three distinct loading types: Cyclic, Monotonically oscillatory (Mono.), and the OU process (OU). We evaluated performance through cross-testing: training the network exclusively on one loading type and testing its extrapolation accuracy on the remaining two.

For the simpler Maxwell model, cross-testing revealed that training TIP-INN with any single loading type (Cyclic, Mono., or OU) resulted in almost the same stress and free energy predictions on the unseen loading types. The single internal variable in the Maxwell model is easily excited, making generalization relatively insensitive to the specific choice of the training path.

The Burgers model presents a more complex energy landscape. If the system is assumed to possess only 1 ISV, large extrapolation errors are observed in both predicted stress and free energy across all testing scenarios, regardless of the training protocol. When the correct capacity of 2 ISVs is assumed, divergent behavior emerges depending on the volume of training data:

- **Single Loading Path (Data-Poor Regime):** When trained on a *single* loading path, the OU protocol vastly outperforms the Cyclic and Mono. protocols. While the predicted stress responses are highly accurate across all three test cases, the model trained on the single OU path yields significantly more accurate free energy predictions on unseen data (shown in Figure S6). This confirms that for limited experimental constraints, the continuous stochastic excitation of the OU process optimally generalizes the network to unseen responses.

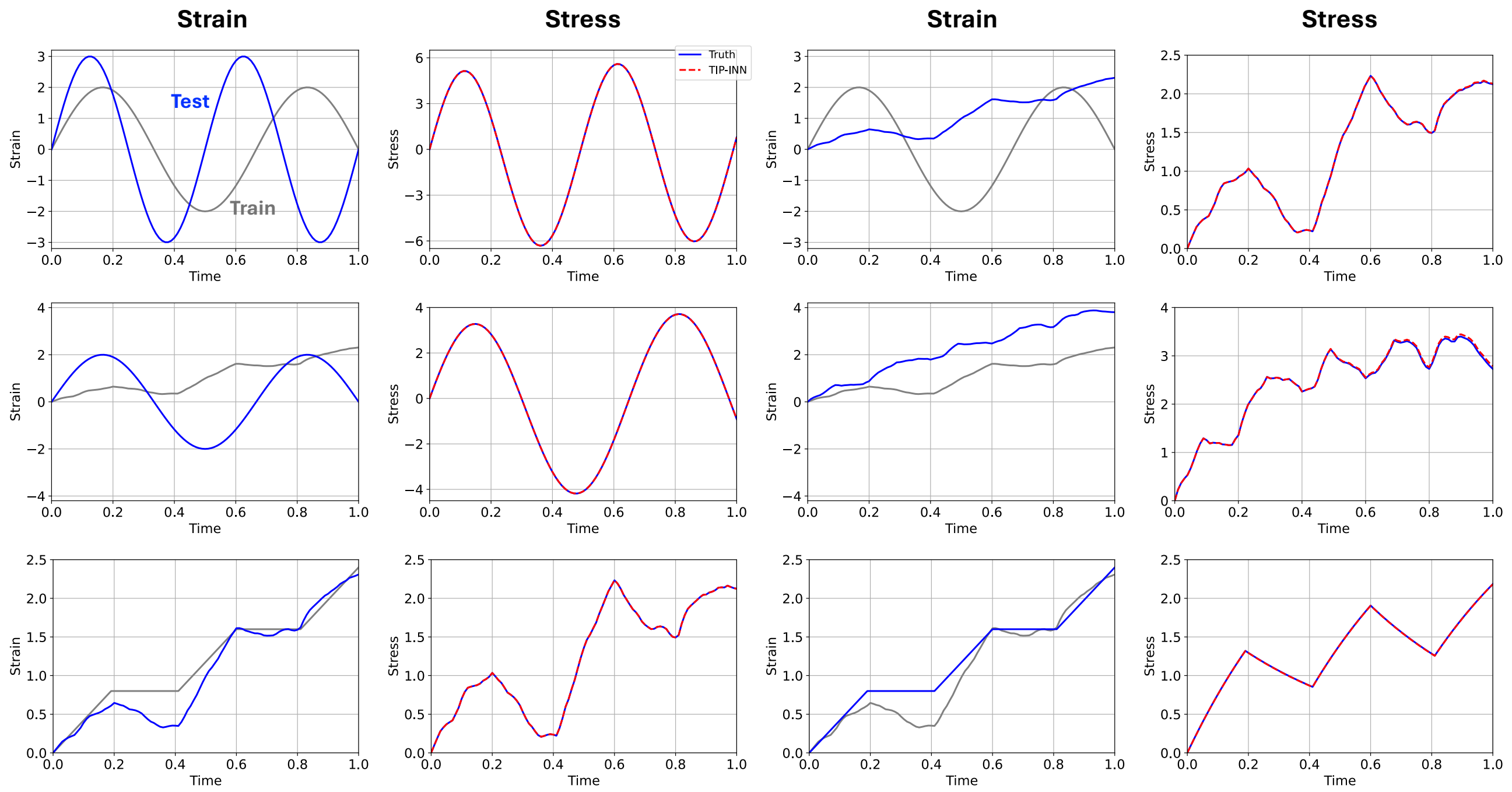

Fig. S2. Maxwell viscoelastic model: Predicted stress for different loading types by training TIP-INN with a single load.

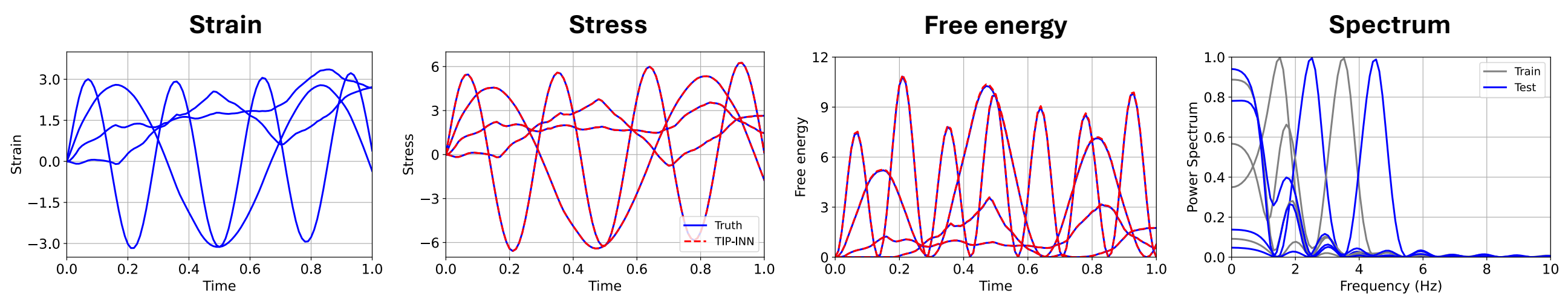

Fig. S3. Maxwell viscoelastic model: Predicted stress and Helmholtz free energy on the training data, along with the power spectral analysis on the training and testing data.

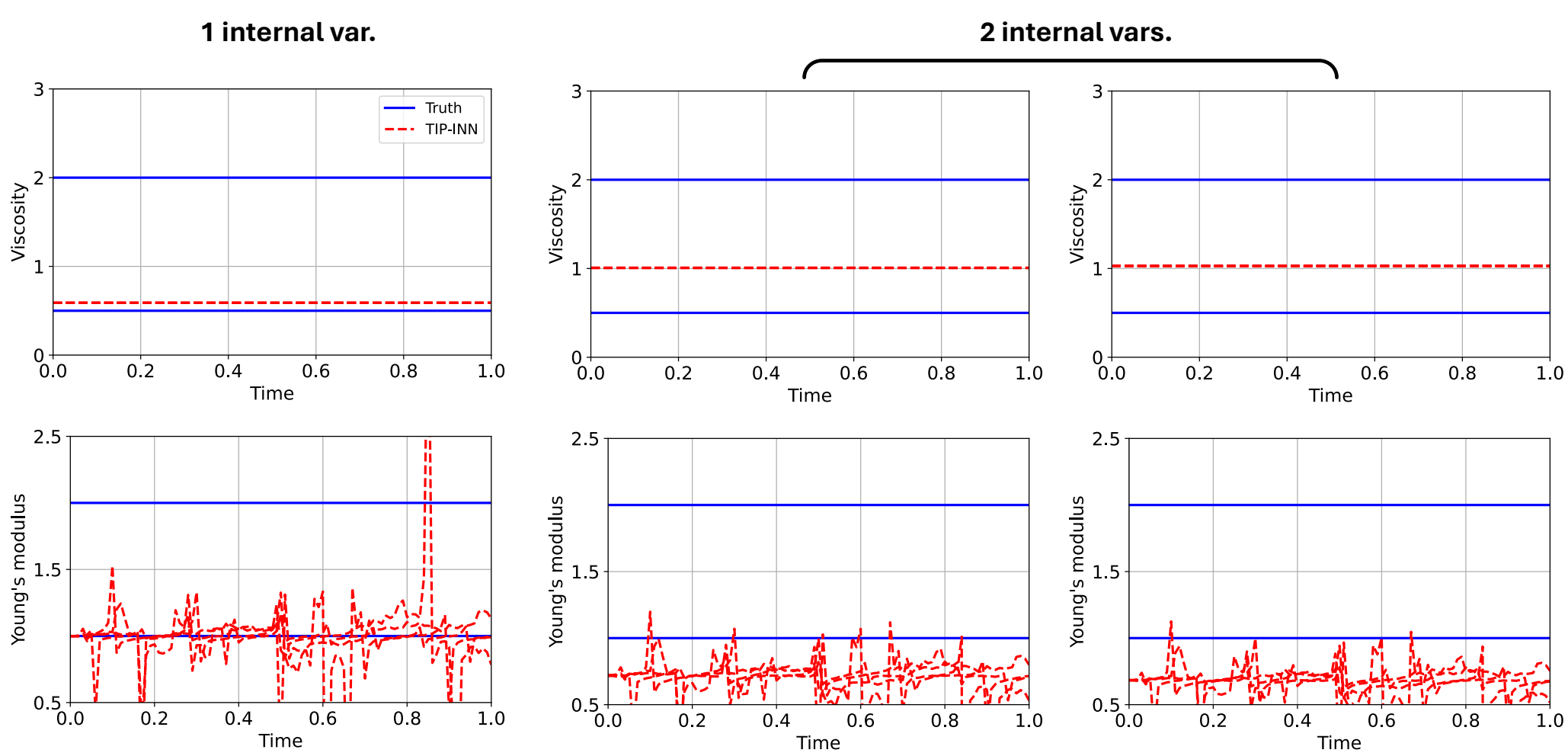

Fig. S4. Burgers viscoelastic model: Predicted viscosity and Young's modulus for Burgers viscoelastic model on the testing data by assuming one or two internal variables in the system.

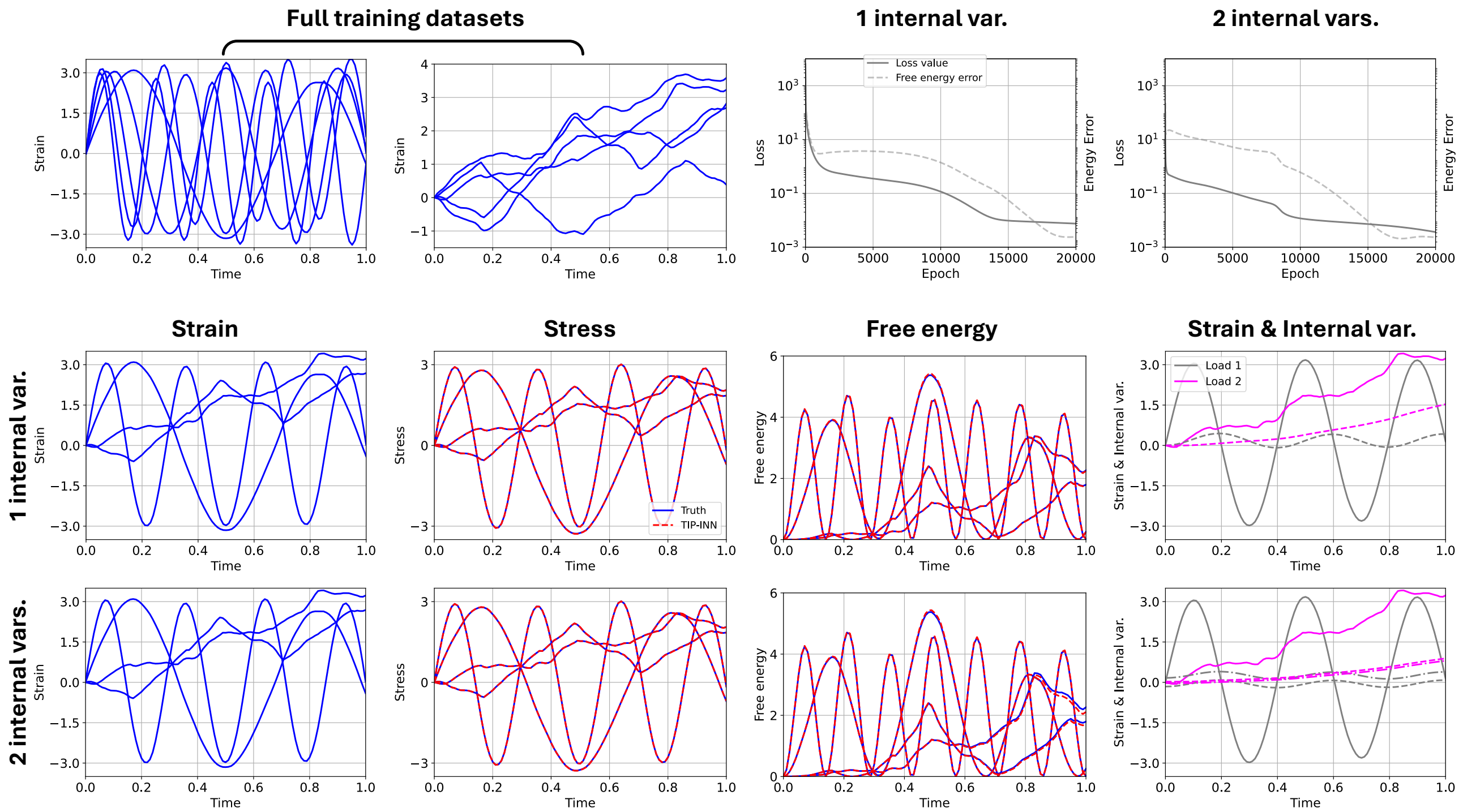

Fig. S5. Burgers viscoelastic model: Cyclic and monotonically oscillatory loads that were used to train TIP-INN. Predicted stress, Helmholtz free energy and internal variables on the training data by assuming one or two internal variables in the system.

- **Multiple Loading Paths (Data-Rich Regime):** Conversely, when TIP-INN is trained using *multiple* varied loading paths of the same type, the standard Cyclic loads yield the most accurate predicted free energy, followed by Mono., and finally the OU process (shown in Figure S7).

These observations offer a highly practical guideline for data-driven constitutive discovery. If physical or financial constraints limit an experiment to a very small number of tests, using an OU process-based loading protocol maximizes the extraction of internal physical variables and ensures robust generalization. However, if a large volume of experimental data can be gathered, the specific structural type of the loading path becomes less critical, and training across a diverse array of standard cyclic loads yields exceptional model fidelity. This exploration of the OU process remains preliminary. The optimal tuning of the stochastic parameters (θ_{OU} and σ_{diff}) is likely model- and material-dependent, representing a promising avenue for future active-learning frameworks.

Extended ISV and Coda wave constraints on the experimental data

To fully explore the optimal network architecture and investigate the potential of incorporating additional acoustic features as physical constraints, we performed an extended analysis on the experimental dataset. Specifically, we tested whether increasing the system capacity to four Internal State Variables (ISVs), or employing the acoustic coda wave as a strict constraint for an additional ISV, would improve the fidelity of the constitutive predictions.

Figure S8 summarizes the predicted macroscopic stress responses under these extended configurations. The plots are organized into three rows, corresponding to models assuming 2, 3, and 4 ISVs, respectively. Within each row, we compare a model constrained solely by the primary P-wave velocity (V_p) against a model simultaneously constrained by both V_p and the acoustic coda wave.

Two primary observations emerge from this analysis: (1) As established in the main text, increasing the number of ISVs from 1 to 3 progressively improves the accuracy of the stress prediction. However, introducing a 4th ISV (Figure S8, bottom row) causes the prediction accuracy to drop below that of the 3-ISV model. This degradation indicates that three ISVs optimally capture the underlying physical deformation mechanisms (e.g., elasticity, bulk damage, and localized fracture) for this specific loading path, while a 4-ISV configuration likely leads to over-parameterization and overfitting. (2) Across all tested architectures (2, 3, and 4 ISVs), explicitly enforcing the acoustic coda wave as a targeted trajectory for an internal variable consistently degrades the accuracy of the predicted macroscopic stress. While the primary velocity (V_p) serves as a reliable, monotonically decreasing proxy for bulk stiffness degradation (a primary thermodynamic ISV), the coda wave represents highly scattered, complex, and path-dependent acoustic energy. Our results suggest that the coda wave does not correlate monotonically or simply with a single, bulk macroscopic thermodynamic state variable. Consequently, forcing the network to mathematically equate the coda wave to an ISV forces the architecture into physically inconsistent states, thereby lowering the overall accuracy of the stress predictions.

These findings highlight a critical consideration for active-learning and multimodal constitutive discovery: while ancillary acoustic features are extremely valuable for regularizing ill-posed ISV inversions, they must represent physically coherent

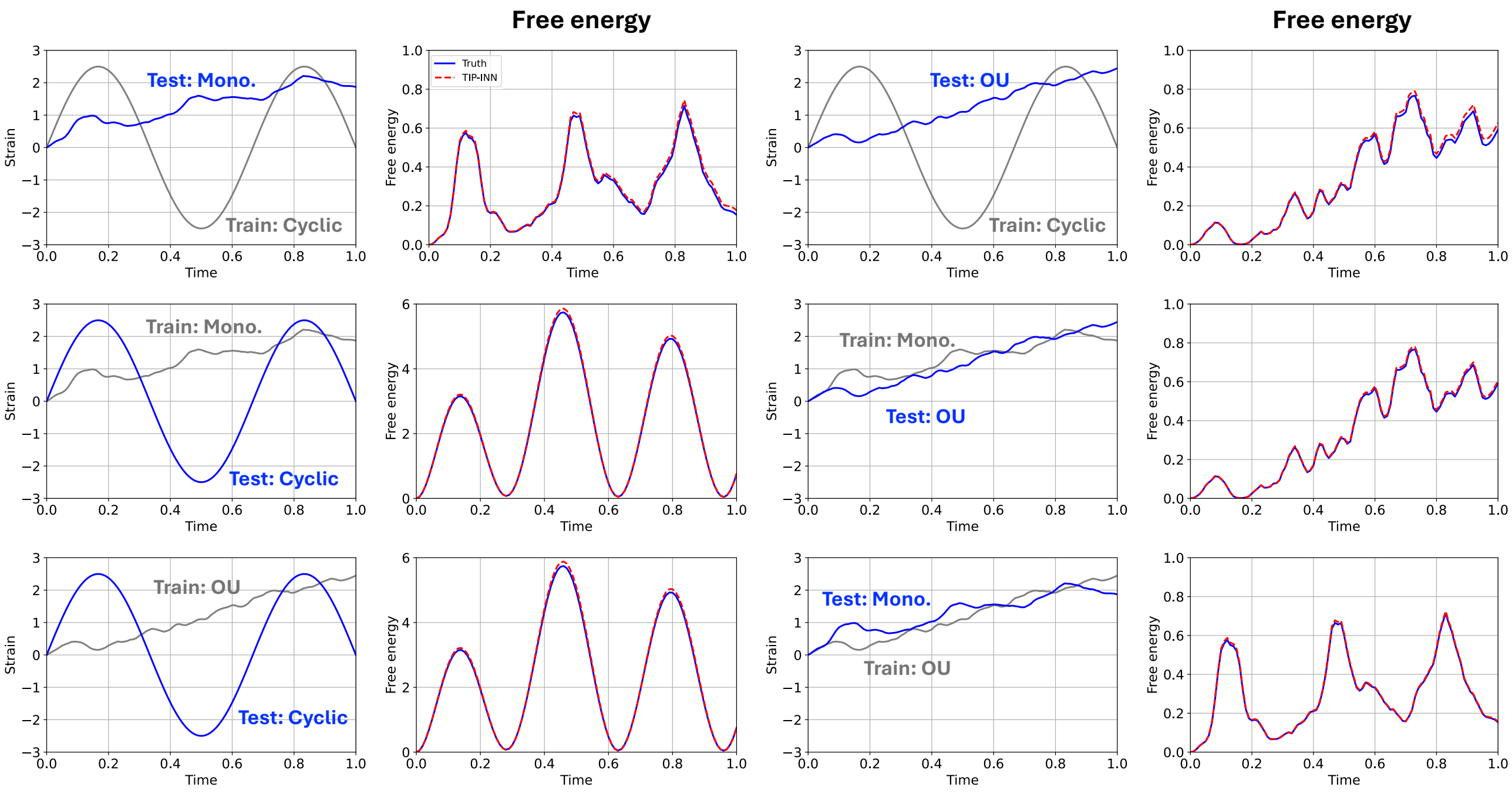

Fig. S6. Burgers viscoelastic model: Predicted Helmholtz free energy (Ψ) for Burgers viscoelastic model on the testing data by training TIP-INN with a single loading path. Note that the predicted stresses ($\boldsymbol{\sigma}$) are accurate in all cases and therefore omitted.

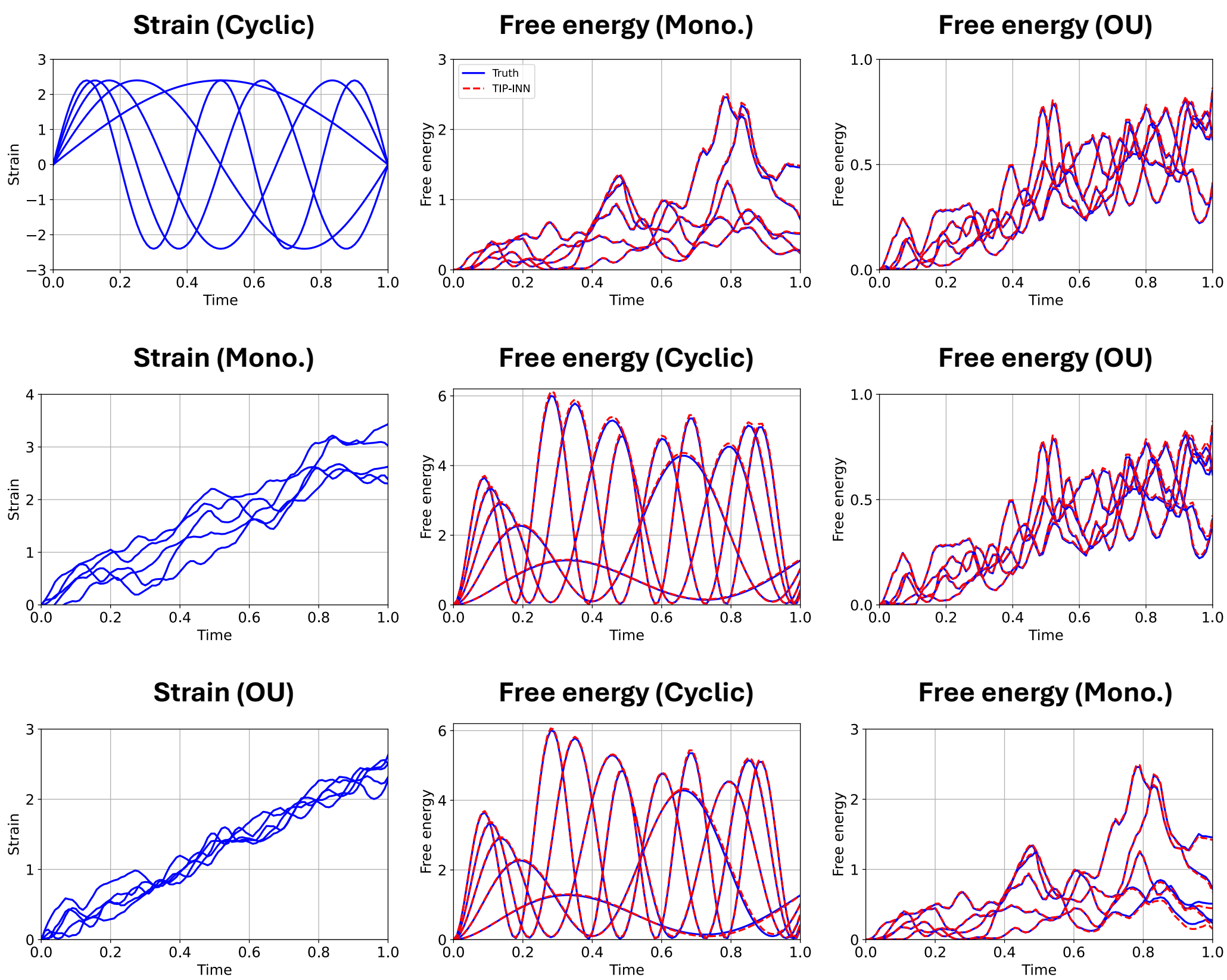

Fig. S7. Burgers viscoelastic model: Predicted Helmholtz free energy (Ψ) for Burgers viscoelastic model on the testing data by training TIP-INN with multiple loading paths of the same type. Note that the predicted stresses ($\boldsymbol{\sigma}$) are accurate in all cases and therefore omitted.

thermodynamic quantities to successfully improve macroscopic constitutive modeling.

Acoustic feature extraction and the coda-wave pipeline

The ultrasonic survey co-recorded with the mechanical loading of sample p5914 provides a set of waveform observables that we use to probe the physical content of the learned internal state variables. Transmitted pulses are digitized at 12 MHz (0.083 μs sampling, 4096 samples per record spanning 341.3 μs), while the mechanical channels (stress, strain, and picked velocity) are logged at about 1 kHz. We extract two classes of feature. The first-arrival features come from the direct, ballistic wave: the picked P-wave velocity $V_p = L/t_t$ with L the through-sample path length (21.8 to 24.5 mm, decreasing with compaction) and t_t the manually validated travel time, together with the pulse amplitude, the dominant frequency, and a frequency-weighted amplitude. The coda features come from the multiply-scattered later arrivals.

Coda waves are the multiply-scattered field that follows the direct arrivals. Above roughly 100 kHz the transmitted field is in the multiple-scattering regime (the coda spectral centroid is about 0.7 MHz), so the coda samples the whole specimen volume along many long, repeated paths and is far more sensitive to a small distributed velocity change than the first arrival [30, 29]. We define the coda window per pulse from the direct travel time: the start is placed two direct traveltimes beyond the first arrival, $t_{\text{start}} = t_{\text{first}} + 2t_t$ (about 28 μs), which excludes the coherent direct packet, and the end follows the smoothed Hilbert envelope down to twice the late-tail noise RMS, capped at 120 μs length, with a 10% cosine taper at both edges [12]. In practice this places the analyzed coda in roughly the 60 to 80 μs of multiply-scattered energy that follows the primary arrival, well separated in time from the coherent direct pulse, so it samples the specimen volume along many long, repeated paths rather than the single fast ray. Because dv/v estimates depend on window placement, we verified that the reported values are stable against reasonable changes of the window start and end.

We estimate the coda velocity change $\delta v/v$ by the stretching method over this window, with the sign convention that $\delta v/v > 0$ corresponds to a velocity increase. Two reference strategies are contrasted. A fixed reference compares every pulse to the first pulse of the window; here it decorrelates completely within about half an hour (coherence falls to 0.22 and the stretch objective becomes multi-peaked), so it is retained only as a decorrelation and damage record, not as a velocity estimate. Our primary estimator is a rolling reference that compares each pulse to its predecessor and integrates the coherence-gated increments, which stays valid when the coda decorrelates relative to a distant reference [28]. The apparent coda $\delta v/v$ is converted to a modulus change by adding the path-length term dL/L from the measured sample shortening, which removes the geometric contribution of compaction. A first-arrival-aligned variant, in which each pulse is aligned to the reference on the direct-arrival window before stretching, removes the bulk direct-ray delay and isolates the part of the velocity change that is not uniform along the ray, which we use as a heterogeneity diagnostic. Beyond $\delta v/v$ we store the standard per-pulse coda scalars over the same window: the decorrelation, the coda RMS amplitude, the inverse coda quality factor Q_c^{-1} from the envelope decay rate, and the coda spectral centroid. The stretching implementation passes a synthetic benchmark suite that pins the sign convention, recovers an imposed $\delta v/v$ to within the search resolution, and confirms the geometry correction and the first-arrival-alignment identity.

Do acoustic features proxy the model internal state variables?

In the generalized standard material formalism that underlies TIP-INN, the internal state variables (ISVs) are latent coordinates defined only up to a smooth, invertible reparametrization [13]. Any diffeomorphism of the internal coordinates that leaves the free energy and the dissipation potential form-invariant produces an identical stress response, so the learned ISVs carry no unique physical meaning a priori. This gauge freedom is the reason a physical label cannot simply be read off a trained network. It does not, however, prevent an empirical test: one can ask whether the learned coordinates align, after removing the trivially shared loading trend, with independently measured acoustic observables that have a known micromechanical interpretation. We carried out this test on the strain-controlled stable window of sample p5914 (3.977 to 4.664 hr), where the model was trained. Over this window the differential stress rises in steps from 2.6 to 63.8 MPa, the axial strain from 8.7 to 10.3 percent, and nearly every measured quantity, including the ISVs, rises or falls along the same near-monotonic ramp. A raw correlation between an ISV and an acoustic feature therefore measures shared trend, not a specific physical link.

We separate the two contributions with a partial-correlation discriminator built on the Frisch-Waugh-Lovell construction [11, 17]. The Frisch-Waugh-Lovell theorem states that in a regression of a response on a set of covariates partitioned into a target block X and a nuisance block Z, the coefficient on X is recovered exactly by residualizing both the response and X on Z and then regressing one residual series on the other, so the partialling-out isolates the association with X after the common variation in Z has been removed. We apply this as a pass/fail discriminator: we residualize each ISV and each candidate acoustic feature on the two variables that drive the common trend, the P-wave velocity V_p and the axial strain, then take the correlation of the residual series, which is the partial correlation between the ISV and the feature controlling for V_p and strain. This partial correlation is invariant to offset and scale, so it is unaffected by the choice of raw, zero-anchored, or standardized values. A feature whose apparent agreement with an ISV was only the shared ramp collapses to near zero under this test, whereas a feature carrying independent information retains a high coefficient. We restrict the candidate first-arrival set to amplitude and frequency features and exclude timing-derived quantities such as V_p itself, because those already determine the velocity that the model encodes in the velocity-associated ISV and a correlation with them would be circular.

The two free ISVs of the three-ISV model are near-mirror images of one another rather than independent degrees of freedom. A linear fit of one on the other gives $R^2 = 0.969$ with slope -0.875 (Pearson $r = -0.984$), so the model effectively uses a single free internal coordinate expressed twice with opposite sign. Before detrending, all amplitude and frequency features correlate strongly with the free ISVs (mean absolute coefficients 0.81 to 0.87). Controlling for velocity and strain, the amplitude features collapse: the mean absolute partial correlation falls to 0.10 for the raw amplitude, 0.19 for the RMS

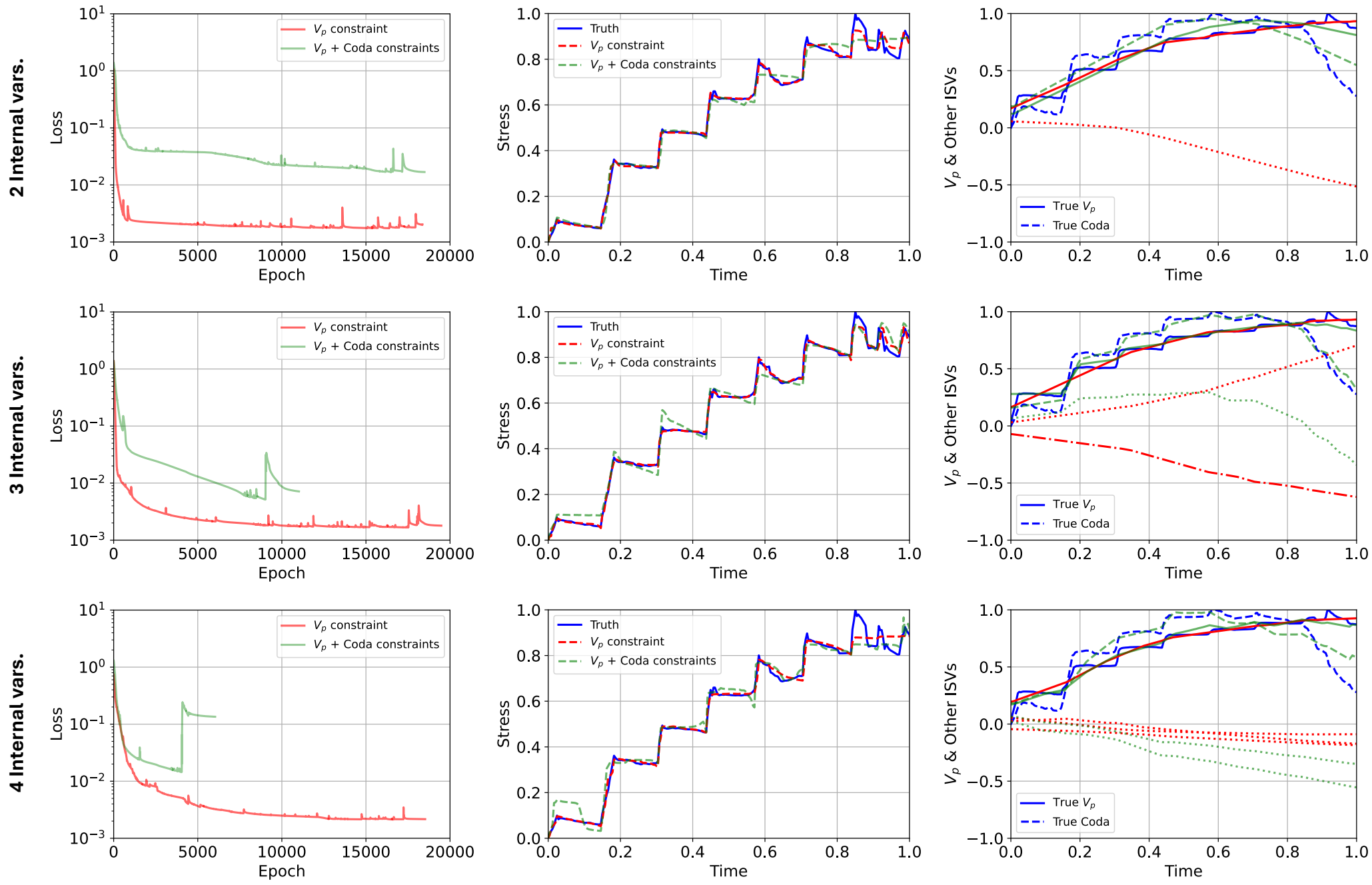

Fig. S8. Comparison of predicted macroscopic stress and internal state variables on the experimental dataset using extended ISV configurations and acoustic constraints. In all cases, incorporating the coda wave as a strict ISV constraint degrades prediction accuracy. Furthermore, increasing the system capacity to 4 ISVs (with only V_p) yields worse stress predictions than the optimal 3-ISV configuration.

amplitude, and 0.21 for the peak amplitude. The dominant frequency is the exception. It retains a partial correlation of +0.767 with the first free ISV and −0.783 with its mirror, down only modestly from its raw value near 0.87, and the frequency-weighted amplitude is intermediate at about 0.43. Among the measured waveform features, the dominant frequency of the direct wave is the only quantitative proxy for the free internal variable (Figure S9).

The coda tells a complementary story. Applying the same discriminator to the coda observables gives a clean negative: no coda feature is a linear proxy for a free ISV. The mean absolute partial correlation, controlling for velocity and strain, is 0.55 for the coda modulus $\delta v/v$, 0.47 for the coda RMS, 0.36 for Q_c^{-1}, 0.34 for the decorrelation, 0.16 for the heterogeneity $\delta v/v$, and 0.07 for the spectral centroid, none reaching the 0.78 the first-arrival dominant frequency attains. The two coda features with the largest partial values are themselves velocity and amplitude derived and behave as suppressors rather than proxies, their partial values exceeding or reversing their raw values. The direct arrival samples the fast path through the specimen, whereas the coda samples the bulk by multiple scattering, so the model variable that tracks the spectral content of the direct wave does not map onto the spatially averaged coda observables. This is consistent with the heterogeneous velocity change of the window: the first-arrival $\delta v/v$ rises monotonically to about +19 percent while the rolling coda modulus $\delta v/v$ peaks near 10 percent around 4.3 to 4.4 hr and then declines to about 3 percent, so the coda change sits far below the one-to-one line a homogeneous stiffening would follow (Figure S10). Aligning the direct arrival before the rolling stretch splits the coda change into a bulk part removed by the alignment (about 6 percent, stepping with the loading) and a residual heterogeneous part of about 3 to 4 percent that is not a uniform delay of the wavefield.

A least-squares analysis confirms and sharpens the correlation result. Ridge regression is used because the predictors are severely collinear (variance inflation factors up to about 10^4 for the amplitude features), which makes ordinary least-squares coefficients unstable. The raw ISVs are predicted almost entirely by strain (in-sample R^2 of 0.90 to 0.92), with V_p raising the velocity-associated ISV to 0.975; because the absolute ISV is a monotonic ramp, this in-sample fit cannot be cross-validated within one loading path. The detrended analysis resolves what the acoustics actually add. For the two free ISVs the acoustic residuals explain about 85 percent of the ISV residual in-sample, and the interleaved (interpolative) cross-validated R^2 is essentially identical at 0.84, so the local co-variation is genuine and reproducible rather than noise fitting. The blocked (extrapolative) cross-validated R^2, however, is negative for every predictor set, the signature of a non-stationary relationship that holds point by point but does not transfer from one contiguous time block to another. Ranking features by single-feature interpolative skill, the dominant frequency of the direct wave is by a wide margin the best predictor of both free ISVs ($R^2 = 0.58$ and 0.60 on its own), ahead of the coda modulus $\delta v/v$ and Q_c^{-1}, while the pure amplitude features carry essentially no independent single-feature signal. The least-squares ranking therefore agrees with the partial-correlation result: the free ISV is carried by the direct-wave dominant frequency, not by coda observables or amplitude.

Physical interpretation of the internal state variables

These results give the learned internal state a physical reading that follows the generalized standard material decomposition of energy, internal variable, and dissipation, and taken together they point to a staged sequence in which three

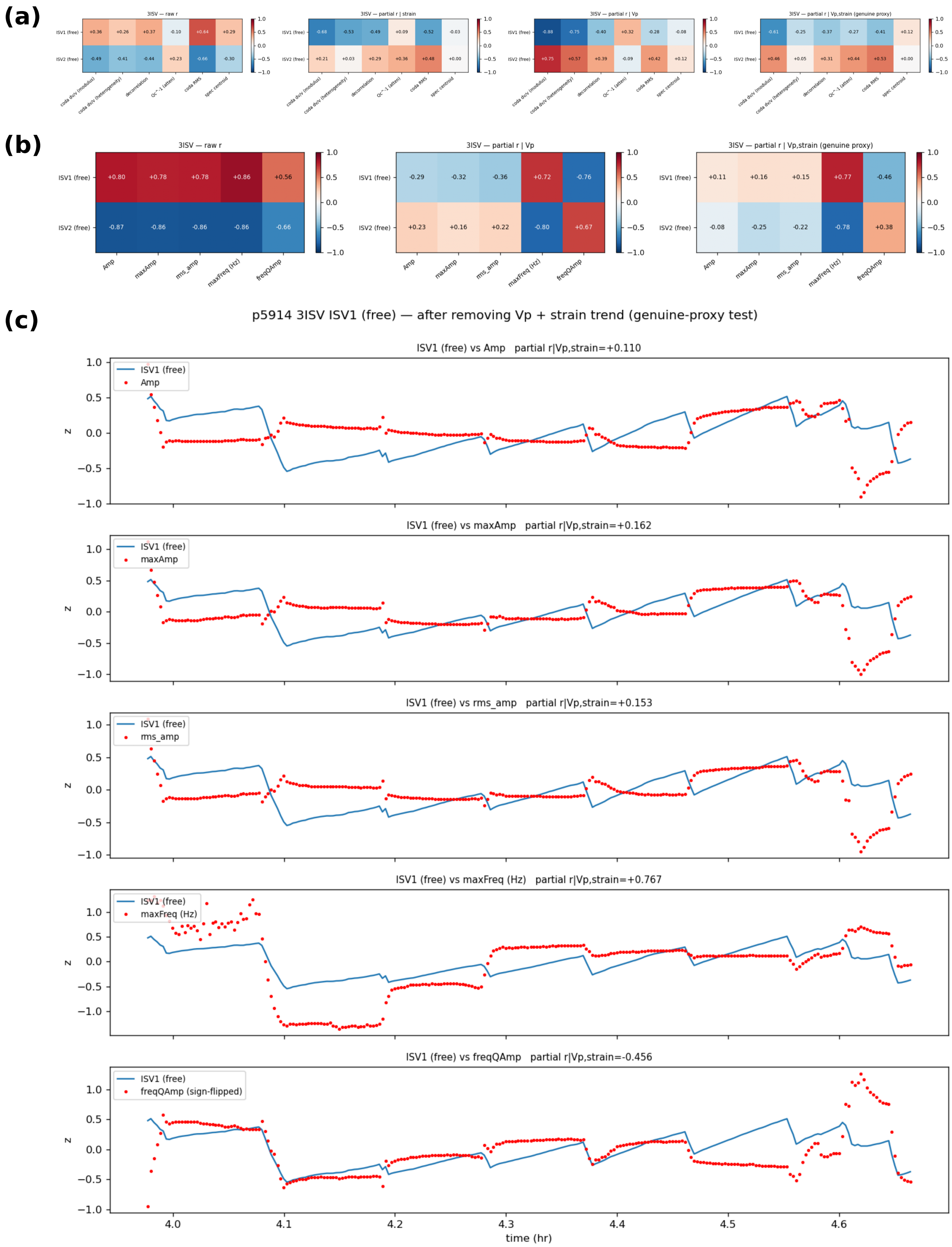

Fig. S9. Assignment of acoustic features to the internal state variables by partial correlation. (a) The discriminator applied to the coda observables, shown as raw r, partial on strain, partial on V_p, and partial on V_p and strain (the genuine-proxy column). Every coda feature collapses toward zero in the genuine-proxy column, so no coda observable is a quantitative proxy for a free ISV. (b) The same discriminator for the first-arrival features (raw, partial on V_p, partial on V_p and strain). The dominant frequency (maxFreq) retains a high coefficient (+0.77 against ISV1, −0.78 against its mirror ISV2) while the amplitude features collapse. (c) Visual confirmation for ISV1: after the V_p and strain trend is removed, the residual free ISV1 is tracked by the maxFreq residual (partial $r = +0.767$) but not by the amplitude residuals, identifying spectral content as the proxy for the free internal coordinate.

acoustic observables become diagnostic of three distinct micromechanical processes in turn. In the first stage the velocity-associated ISV reproduces V_p by construction and represents the recoverable elastic stiffness, the curvature $\partial^2\Psi/\partial\boldsymbol{\epsilon}^2$ of the stored energy, set by progressive crack closure and contact stiffening under rising mean stress [21, 22]. This is the earliest and most robust link, and it is why V_p alone already serves as an effective constraint on the damage ISV. In the second stage the two free ISVs align not with amplitude but with the dominant-frequency shift of the transmitted pulse. The dominant frequency is governed by scattering and intrinsic attenuation rather than by bulk modulus [32], and it is controlled by cracks and scatterers on or near the fast, near-direct path together with the evolving spatial distribution of damage, which progressively reddens the transmitted spectrum. We therefore read the free coordinate as a spectral or scattering memory variable that tracks the developing damage distribution independently of the modulus during strain-controlled deformation. Amplitude is not the carrier of this second mode; frequency is.

The third stage is recorded by the coda, which samples the whole specimen volume by multiple scattering and becomes diagnostic only later, once damage is extensively and heterogeneously distributed through the sample rather than concentrated near the fast path. Figure S11 shows that the coda modulus $\delta v/v$ turns over near 4.39 hr while the first arrival keeps rising, and that this turnover coincides with the extremum of the ISV1-ISV2 off-diagonal residual near 4.37 hr, that is, with the breakdown of the mirror symmetry between the two free coordinates. The two times agree to about two minutes, which is coincident to within the coda sampling interval and the resolution of the measurement given the temporally varying baseline, so the agreement is best read as coincident within the coda sampling cadence rather than as a precise two-minute match. The standard deviation of that residual doubles from the first half of the window to the second, the coda modulus $\delta v/v$ anti-correlates with it at $r = -0.78$, and the heterogeneity $\delta v/v$ ($r = -0.85$) and Q_c^{-1} ($r = +0.79$) track it as strongly, while the coda RMS and spectral centroid do not. We read this as a single late-window regime change: around 4.4 hr the model begins to use its second free degree of freedom at the same time the bulk velocity sensed by the coda stops increasing and reverses, attenuation rises, and the heterogeneity residual peaks. Physically this is a shift from progressive crack closure along the fast path toward distributed damage and scattering as the sample approaches the end of the stable interval. The first arrival, weighted toward the stiffest closed-crack population, keeps stiffening, while the volume-averaging coda records the onset of distributed damage the fast path does not yet feel.

A future path: gauge-symmetric weak supervision of the internal variables

The analysis above establishes a real but non-stationary link between the acoustic features and the free ISV within one experiment. Turning that link into a validated, transferable constraint is a natural next step, and it clarifies why the acoustic channel must enter the model softly rather than as a hard equality. Section S6 showed that forcing an acoustic feature to equal an ISV degrades the stress fit: a hard equality over-determines the state because it fixes both the gauge and the amplitude of a latent coordinate to a single observable that itself carries a dynamic-static modulus offset and a lapse-time dependence, pushing the network into physically inconsistent states.

A more promising architecture, which we leave for future work, is weak supervision through a learnable gauge. One equips each internal coordinate with an affine map $\alpha_k \mapsto c_{0k} + c_{1k}\,\alpha_k$ whose coefficients are learned jointly with the network, and matches the gauged coordinate to one acoustic observable through a soft penalty added to the loss rather than an equality constraint. The affine map absorbs exactly the reparametrization freedom of the generalized standard material and the static-dynamic offset between the modulus the network infers from stress and the modulus a wave samples, so the acoustic channel acts as a regularizer that breaks the identifiability degeneracy without competing with the mechanical fit. The physical hypothesis such an architecture would test is specific and falsifiable: that the internal state separates into an elastic-stiffness coordinate anchored by V_p, a scalar-damage coordinate carried by the coda $\delta v/v$, and a third, acoustically silent creep or back-stress coordinate that governs the inelastic stress path without altering the crack population. If borne out, this would make the generalized standard material decomposition observable one coordinate at a time, and it would show that seismic monitoring of V_p, $\delta v/v$, and Q constrains the stiffness and damage state but is intrinsically blind to part of the flow state, which motivates joint mechanical-acoustic inversion within a single thermodynamic model. The decisive validation is cross-experiment: fit the gauge on one sample and predict on a broader set of independent experiments, since the within-sample evidence shows the acoustic-to-ISV link is reproducible under interpolation but not yet validated as an extrapolative model that transfers across the full experimental set.

Gibbs free energy

In this work, we consider the sign-flipped Gibbs free energy, that is, $G^{+}(\boldsymbol{\sigma},\boldsymbol{\alpha}) = -G(\boldsymbol{\sigma},\boldsymbol{\alpha})$ (see Eq. 13), to model isothermal problems. G^{+} is non-negative and convex with respect to $\boldsymbol{\sigma}$ and $\boldsymbol{\alpha}$. Therefore, the same neural networks used to predict the Helmholtz free energy and dual dissipation in TIP-INN can be applied to predict G^{+} here. Figure S12 shows the detailed architecture for predicting G^{+} with the loss function defined as

$$\mathscr{L}_{\text{Gibbs}} = \mathscr{L}_{\boldsymbol{\epsilon}} + \mathscr{L}_{\text{LF}} + \mathscr{L}_{G^{+}_{+}} + \mathscr{L}_{G^{+}(0)} + \mathscr{L}_{\boldsymbol{\epsilon}(0)} + \mathscr{L}_{L_2}\ ,$$

where

$$\mathscr{L}_{\boldsymbol{\epsilon}} = \frac{1}{N}\sum_{n=1}^{N}\Big(\boldsymbol{\epsilon}_{\theta}(n) - \boldsymbol{\epsilon}(n)\Big)^2\ , \quad \mathscr{L}_{\text{LF}} = \frac{1}{N}\sum_{n=1}^{N}\Big(G^{+}_{\theta}(n) - G^{+}\Big)^2\ ,$$

$$\mathscr{L}_{G^{+}_{+}} = \frac{1}{N}\sum_{n=1}^{N}\Big\langle -G^{+}_{\theta}(n)\Big\rangle\ , \quad \mathscr{L}_{G^{+}(0)} = \Big(G^{+}_{\theta}(n=1; t=0)\Big)^2\ ,$$

$$\mathscr{L}_{\boldsymbol{\epsilon}(0)} = \Big(\boldsymbol{\epsilon}_{\theta}(n=1; t=0)\Big)^2\ , \quad \mathscr{L}_{L_2} = \frac{1}{N_w}\sum_{n=1}^{N_w}\Big(w_i\Big)^2\ .$$

$\mathscr{L}_{\boldsymbol{\epsilon}}$ and $\mathscr{L}_{\text{LF}}$ are the losses for the strain and Legendre-Fenchel transformation, respectively. Other loss functions are defined in the same way as in Eq. 17. Figure S12 shows that accurate G^{+} are predicted by the convex neural network for the Maxwell viscoelastic model. This numerical example confirms that different types of thermodynamic potentials can be learned using the same computational frameworks that were proposed in this work.

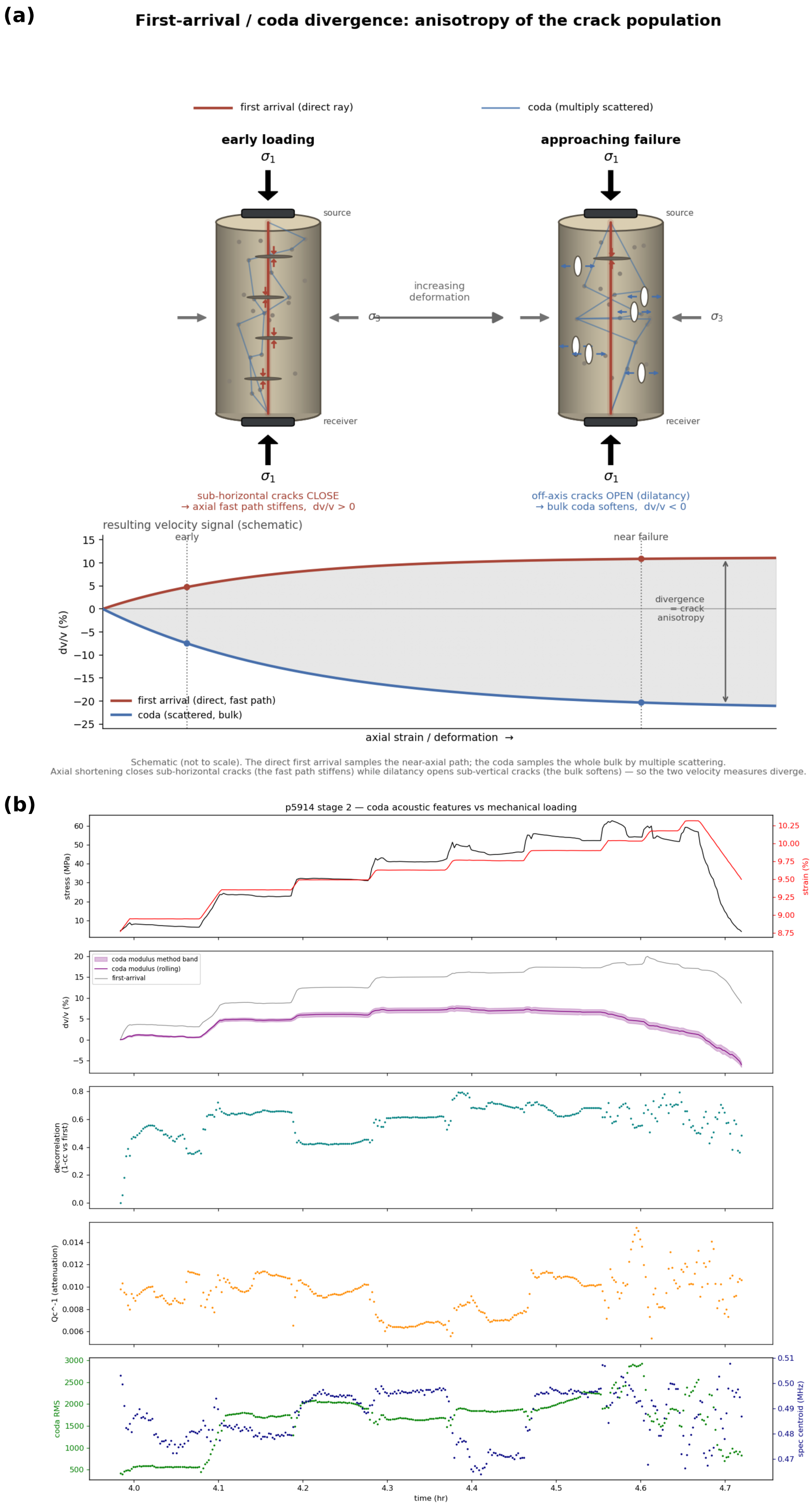

Fig. S10. Physical basis for a divergence between the first arrival and the coda. (a) Conceptual schematic: under rising differential stress, crack closure concentrates along the fast, stiff path sampled by the first arrival, so V_p stiffens strongly, while the multiply-scattered coda samples the whole volume and records a smaller, heterogeneous change. (b) Measured coda-feature time series for p5914 over the stable window, showing the first-arrival $\delta v/v$ rising monotonically while the coda modulus $\delta v/v$ peaks and declines, together with decorrelation, Q_c^{-1}, and coda amplitude and centroid against the stress and strain path.

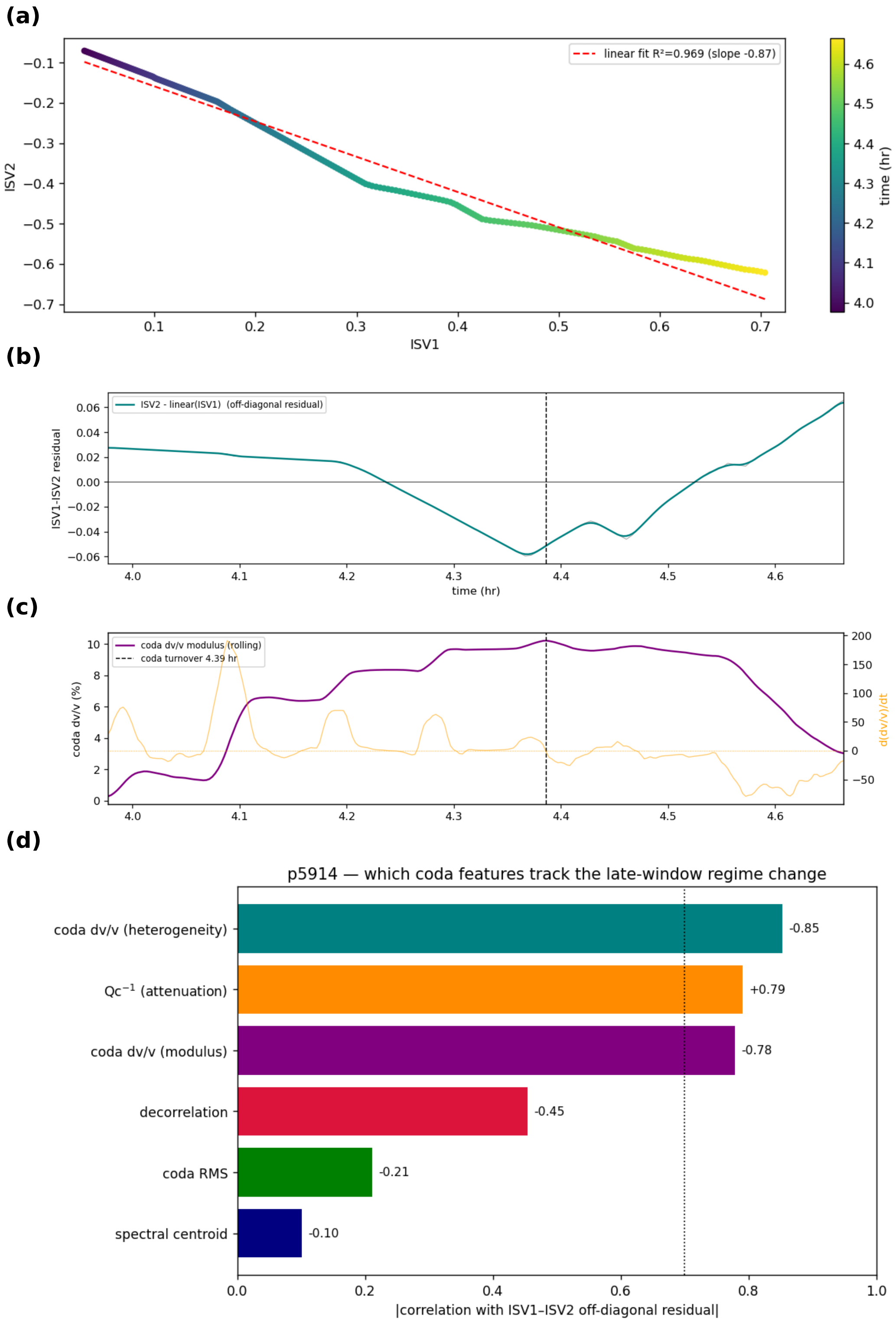

Fig. S11. Coda signature of the late-window regime change. (a) The two free ISVs traced against one another and colored by time: over the window they follow a near-perfect mirror line ($R^2 = 0.969$, slope -0.87), so the model uses effectively one free internal coordinate expressed twice with opposite sign. (b) The off-diagonal residual ISV2 − linear(ISV1), the departure from that mirror line, reaches its extremum near 4.37 hr, marking where the mirror symmetry between the two free coordinates breaks down. (c) The coda modulus $\delta v/v$ (rolling) and its time derivative: the coda-sensed velocity turns over near 4.39 hr, within about two minutes of the residual extremum and hence coincident to within the coda sampling interval, while the first arrival keeps rising. (d) Absolute correlation of each coda feature with the off-diagonal residual: the coda heterogeneity $\delta v/v$ (-0.85), Q_c^{-1} ($+0.79$), and modulus $\delta v/v$ (-0.78) track the regime change most strongly, whereas the coda RMS and spectral centroid do not.

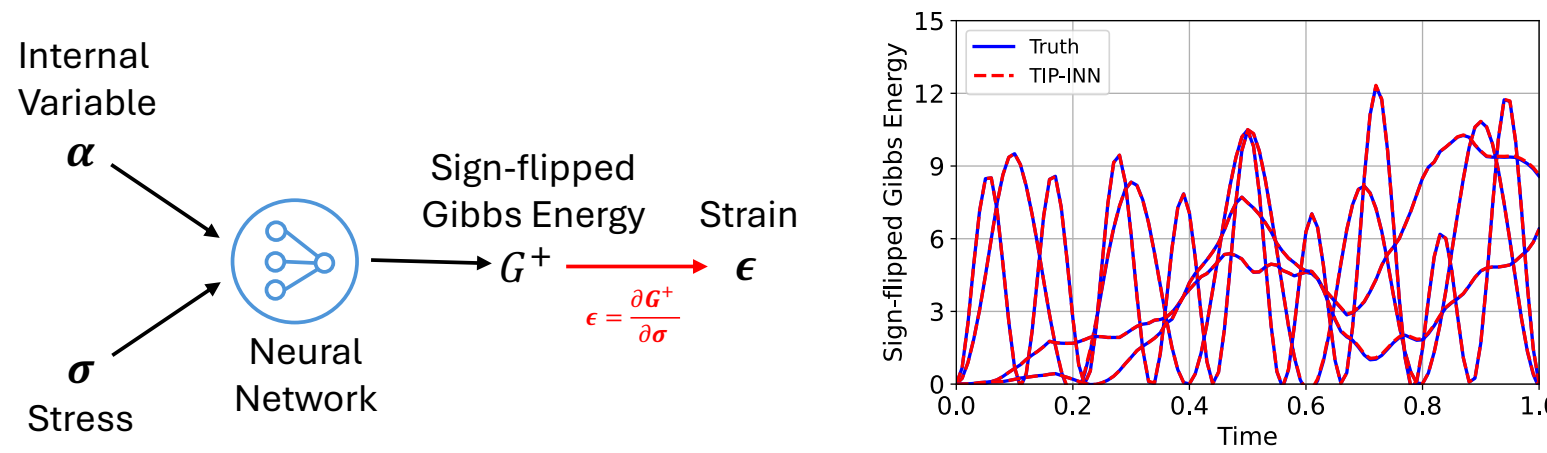

Fig. S12. Schematic illustration of the convex neural network for predicting sign-flipped Gibbs free energy, G^+ (left) and the predicted G^+ (right) for the Maxwell viscoelastic model.

Elasto-plastic and visco-plastic materials

In order to demonstrate the versatility of TIP-INN, we have expanded our benchmarks to include both rate-independent plasticity and rate-dependent viscoplasticity. We consider the Prandtl elastoplastic model, which exhibits sharp transitions in the stress-strain relationship, with Young's modulus $E = 1$ GPa and yield stress $\boldsymbol{\sigma}_p = 0.5$ GPa. We also evaluate the Perzyna viscoplastic model, featuring $E = 2$ GPa, viscosity $\eta = 0.1$ GPa·s, and yield stress $\boldsymbol{\sigma}_p = 0.5$ GPa. A single internal variable is employed in TIP-INN for both cases, and we evaluate the framework's performance using both single-dataset and multi-dataset (8 loading paths) training regimes.

Figure S13 shows the predicted stress, Helmholtz free energy, and internal variables (plastic strains) for both models. For a single training dataset (Figure S13, top and middle rows), the stress is accurately captured in both scenarios; however, notable errors are observed in the predicted Helmholtz free energy. Compared to the elastoplastic model, TIP-INN yields more accurate predictions for the free energy and internal variable in the viscoplastic case, owing to the inherent mathematical smoothness of the rate-dependent viscoplastic transition. Nevertheless, as the material response evolves from elasticity to plasticity, the continuous Neural ODE integrator struggles to perfectly capture the sharp, non-smooth yield surface, leading to discrepancies in the free energy potentials.

When further trained on multiple diverse datasets (8 loading paths), we observe divergent behavior between the two models. For the elastoplastic model, training with additional datasets does not improve the accuracy of the predicted free energy or internal variables. Conversely, for the viscoplastic model (Figure S13, bottom row), incorporating multiple loading paths noticeably increases the accuracy of the predicted free energy. Nonetheless, owing to the persistent nonlinearities introduced by yielding, these predictions remain less accurate than those achieved for purely viscoelastic models (e.g., Maxwell or Burgers models).

Extensive testing confirmed that increasing the network depth, modifying the activation functions, or increasing the number of internal variables does not significantly improve the accuracy of the predicted Helmholtz free energy for both models. This limitation stems fundamentally from the continuous nature of the neural integrator. Replacing the neural integrator with a discrete, Newton-based return-mapping algorithm yields highly accurate predictions of the yield surface [9], but incurs a substantially higher computational cost and sacrifices the robust training dynamics provided by continuous-time rollouts.

Benchmark Comparisons (TIP-INN vs GSMN vs TANN)

To comprehensively evaluate the accuracy and efficiency of the proposed framework, the Maxwell viscoelastic and Perzyna viscoplastic models are tested using GSMN [9], TANN [20], and TIP-INN. We train the networks on a single NVIDIA GTX 1660 Super GPU using a cyclic load and test them on both cyclic and monotonically oscillatory loading protocols.

To quantify the predictive accuracy, we compute the L_2 errors of the predicted solutions using the following formula:

$$E_2^{\chi} = \left(\int_0^T (\chi_t - \chi_p)^2 \mathrm{d}\xi \right)^{1/2}, \tag{S4}$$

where χ is a placeholder for either the stress or the free energy. The subscripts t and p denote the true (analytical) and predicted solutions, respectively.

Network Architectures and Hyperparameters. For a fair comparison, the network capacities are matched to the respective architectural requirements of each framework:

- **GSMN:** We use 2 hidden layers with 20 neurons per layer for both the free energy and the dual dissipation networks. As recommended by the original authors, we employ `softplus_squared` activation functions.
- **TANN:** We use 3 hidden layers with 32 neurons each for the evolution equation (using `ReLU` activations), and 3 hidden layers with 64 neurons each for the free energy (using `GELU` activations).
- **TIP-INN:** For the Maxwell model, the hyperparameters remain identical to those presented in the main text. For the Perzyna model, we increase the capacity to 3 hidden layers with 20 neurons each, keeping all other settings constant.

These settings are applied consistently across both the Maxwell and Perzyna benchmark cases.

Tables S1 and S2 summarize the L_2 errors for the stress and free energy across the training and testing datasets, alongside the Wall-Clock Time (WCT) per 1000 epochs. For the Maxwell model, TIP-INN and GSMN achieve very similar L_2 errors on the training data. However, TIP-INN dramatically outperforms GSMN on the testing data, reducing the L_2 error by approximately two orders of magnitude for both stress and free energy. This discrepancy arises because GSMN relies on a local Newton-Raphson method within its architecture. While this ensures a robust forward pass, it imposes rigid constraints that hinder generalization. By utilizing a neural ODE, TIP-INN provides a more flexible and continuous integration of the internal state variables, facilitating superior extrapolation to unseen data.

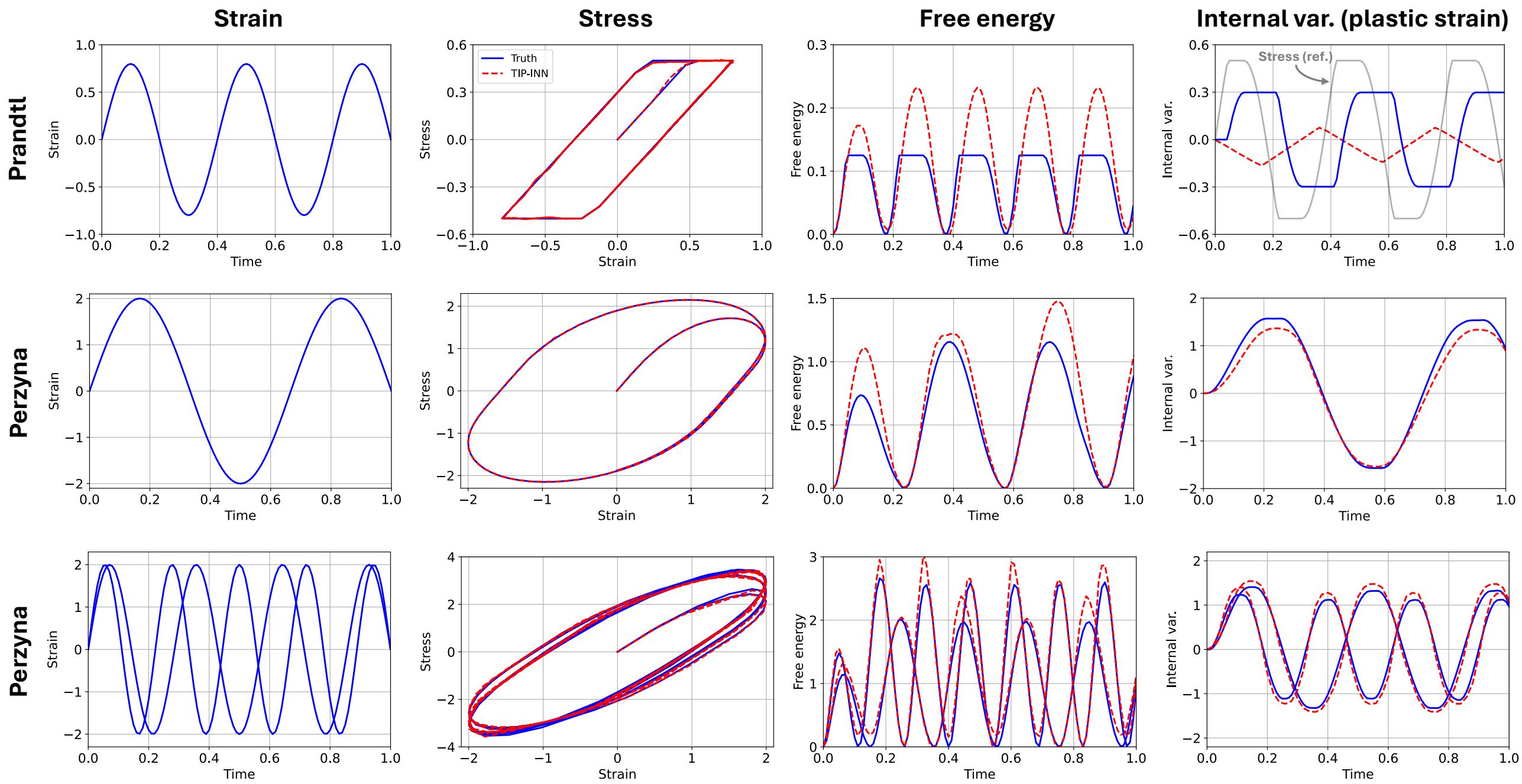

Fig. S13. Prandtl elastoplastic and Perzyna viscoplastic models: Predicted stress, Helmholtz free energy, and internal variables (plastic strain). The top row displays results for the elastoplastic model trained on a single dataset. The middle and bottom rows display results for the viscoplastic model trained on a single dataset and multiple datasets (8 loading paths), respectively.

Table S1. Benchmark results for the Maxwell viscoelastic model. L_2 errors are reported for stress and free energy across training and two testing datasets. WCT represents the Wall-Clock Time per 1000 epochs in seconds.

*Model	Stress L_2 Error			Free Energy L_2 Error			*WCT
	Train	Test 1	Test 2	Train	Test 1	Test 2	
**TIP-INN**	1.38e-3	2.92e-3	2.11e-3	2.12e-2	5.07e-2	9.31e-3	249
**GSMN**	1.19e-3	3.95e-1	2.04e-1	3.08e-1	1.01e0	4.91e-1	6,629
**TANN**	3.33e-1	1.21e0	7.86e-1	8.35e0	1.35e1	2.17e0	477

Conversely, TANN exhibits much larger errors for both training and testing datasets, typically 2 to 3 orders of magnitude larger than those of TIP-INN. Because TANN enforces thermodynamic consistency entirely through soft-penalty loss functions rather than architectural convexity, it is highly prone to overfitting and struggles to accurately capture the underlying physics of the multi-variate system.

For the Perzyna viscoplastic model, TIP-INN and GSMN yield very similar L_2 errors on both the training and testing data. It is worth noting that we did not implement a specialized Newton method with return mapping for the GSMN benchmark; incorporating such a return mapping algorithm could potentially improve GSMN's viscoplastic predictions. Nevertheless, TANN continues to produce substantially larger L_2 errors compared to both GSMN and TIP-INN.

Finally, in terms of computational efficiency, TIP-INN trains approximately 30× faster than GSMN and about 2× faster than TANN for the Maxwell model. For the Perzyna model, TIP-INN remains more than 10× faster than GSMN and is on par with TANN. These quantitative results confirm that TIP-INN provides a uniquely optimal balance of strict physical consistency, extrapolation accuracy, and computational scalability. Figure S14 shows the loss values versus the number of epochs for all three methods and compares the predicted stress and free energy for the training and testing data on the Maxwell and Perzyna models.

Sparse Identification of Nonlinear Potentials (SINP)

To overcome the black-box nature of the trained neural potentials, we apply a Sparse Identification of Nonlinear Potentials (SINP) procedure-adapted from the SINDy framework [3]-to distill the learned Helmholtz free energy, Ψ, into closed-form analytical expressions that can be directly imported into continuum geodynamic codes. Figure S15 shows the SINP-calibrated Ψ against the neural predictions. Notice that an exact quadratic Ψ function (Truth: $\Psi = \varepsilon_e^2 = (\varepsilon - \alpha_1)^2$) was discovered for the Maxwell model. It is also observed that the convex and positive properties of Ψ were also maintained by the discovered formulas for other models.

Fitting the data model with non-linear visco-elastic model

This section summarizes the details of the Bayesian framework for identifying non-linear viscoelastic constitutive laws, specifically focusing on the evolution of material properties as a function of strain. We are preparing a separate manuscript

Table S2. Benchmark results for the Perzyna viscoplastic model. L_2 errors are reported for stress and free energy across training and two testing datasets. WCT represents the Wall-Clock Time per 1000 epochs in seconds.

*Model	Stress L_2 Error			Free Energy L_2 Error			*WCT
	Train	Test 1	Test 2	Train	Test 1	Test 2	
**TIP-INN**	9.44e-3	2.36e-1	2.53e-1	1.77e-1	1.90e-1	7.75e-2	587
**GSMN**	9.44e-3	1.57e-1	2.63e-1	2.05e-1	3.66e-1	5.29e-2	7,162
**TANN**	1.64e-1	2.36e0	1.64e0	1.59e1	3.99e1	9.27e0	502

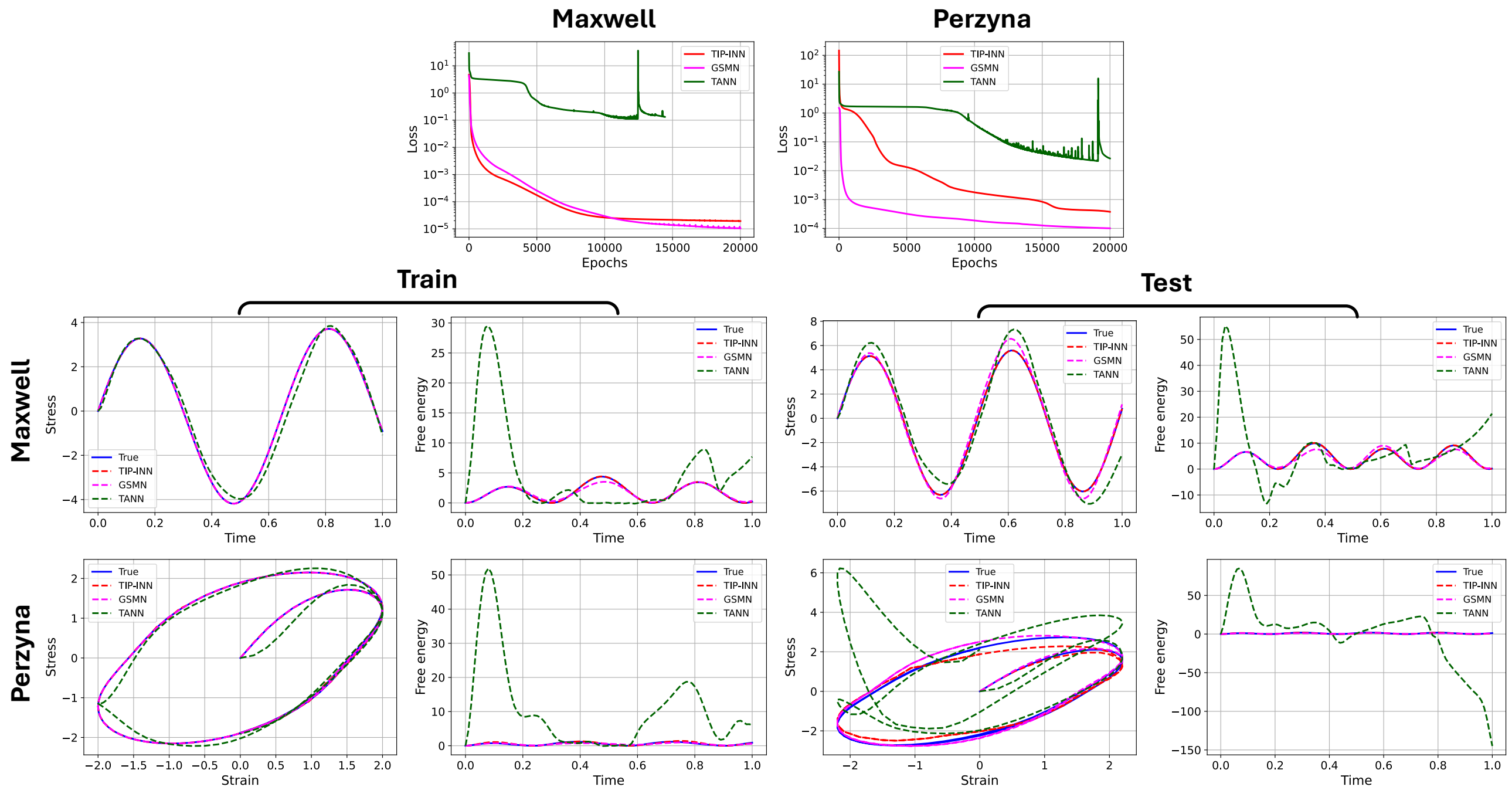

Fig. S14. Comparative performance of TIP-INN, GSMN, and TANN on the Maxwell viscoelastic and Perzyna viscoplastic models. The plots display the training loss convergence versus the number of epochs for each method, alongside a direct comparison of the analytical (ground truth) and predicted trajectories for stress and free energy. Results are evaluated across both the training datasets and the unseen testing datasets to highlight extrapolation capabilities.

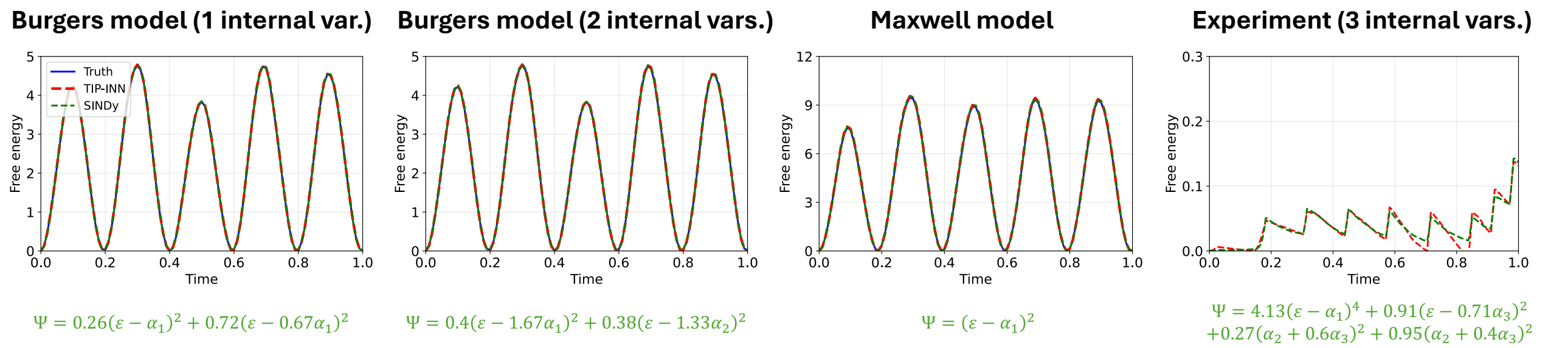

Fig. S15. Calibration of the predicted Helmholtz free energy with the SINP framework for different analytical models and experimental data.

with additional details of the experiments and a larger sample suite of lava flow samples.

Non-Linear Viscoelastic Model Formulation

To capture the time-dependent deformation observed in the Pacaya basaltic lava flows [5], we employ a non-linear viscoelastic framework that extends the generalized Maxwell model [23]. Unlike standard subsurface models that assume static material properties, this approach defines the elastic moduli (E) and viscosities (η) as evolving functions of the internal state, represented here by the cumulative axial strain ($\boldsymbol{\epsilon}$). As seen in the experimental mechanical data, the samples exhibit significant non-linearity and creep that require this dynamic parameterization.

The total stress $\boldsymbol{\sigma}$ at any time t is calculated using a recursive direct-fit method [23], which utilizes a history state variable to bypass the computational cost of storing the full strain history and allow fast MCMC inversion. The governing

equations for the strain-dependent case are:

$$\sigma(\epsilon, t) = \sum_{i=1}^{n} E_i(\epsilon) \frac{d\epsilon_i}{dt} dt + \sum_{i=1}^{n} \left\{ h_i[\epsilon(t), t] e^{-\Delta t/\tau_i} + \frac{E_i(\epsilon)(1 - e^{-\Delta t/\tau_i})}{\Delta t/\tau_i} \frac{d\epsilon_i}{dt} dt \right\} \quad \text{(S5)}$$

Where the history variable h_i represents the viscoelastic state at the previous increment:

$$h_i[\epsilon(t), t] e^{-\Delta t/\tau_i} = \int_0^t \left\{ E_i(\epsilon) e^{-(t-\Delta t+\tau)/\tau_i} \right\} \frac{d\epsilon_i}{dt} dt \quad \text{(S6)}$$

Evolution of Material Potentials

The model accounts for damage-induced degradation, such as pore collapse and microcracking. The long-term elastic modulus is held constant as $E = a$, while each of the viscoelastic moduli E_i (for the elastic element outside the Maxwell element as well as an internal visco-elastic element) and viscosities η_i evolve according to power-law relations based on normalized strain:

$$E_i(\epsilon) = a \left(1 + b \left(\frac{\epsilon}{\epsilon_{max}} \right)^c \right) \quad \text{(S7)}$$

$$\eta_i(\epsilon, t) = d \left(1 + e \left(\frac{\epsilon}{\epsilon_{max}} \right)^f \right) \quad \text{(S8)}$$

The relaxation time τ_i is self-consistently derived from these evolving properties:

$$\tau_i(\eta_i, E_i, t) = \frac{10^{\eta_i(\epsilon)}}{E_i(\epsilon)} \quad \text{(S9)}$$

Bayesian Parameter Identification (MCMC)

To resolve the structural non-uniqueness inherent in rock rheology where multiple parameter combinations can yield similar stress-strain curves and also to ensure robustness against local minima, we utilize a two-stage optimization and inference pipeline. **Stage 1: Global Optimization.** We maximize a log-likelihood function ($\log L$) that quantifies the residuals between experimental stress σ_{data} and model predictions σ_{model}:

$$\log L(\sigma) = -\frac{1}{2} \sum_{i=1}^{n} (\sigma_{data}(t_i) - \sigma_{model}(t_i; \theta))^2 \quad \text{(S10)}$$

The parameter space θ is sampled using the Tree-structured Parzen Estimator (TPE) algorithm via the *Optuna* package [1]. **Stage 2: Posterior Estimation.** The optimal parameters from Stage 1 initialize a Markov Chain Monte Carlo (MCMC) sampler using the *emcee* package [10]. This yields the corner plots (Fig. 3c, d) used to estimate the full posterior probability density function (PDF), ensuring that parameters are well-constrained and physically consistent.

Comparison to Standard Subsurface Models

Typical geodynamic models often rely on linear elastic assumptions that fail to capture the irreversible deformation and creep observed in volcanic basalts [27]. Our results for the Pacaya core and base samples (p5914) demonstrate that elastic moduli can increase by factors as high as 100s as the material compacts or damages [5]. The integration of Bayesian MCMC allows us to quantify the uncertainty in these transitions, identifying the precise moment the material shifts from purely elastic to complex viscoelastic regimes. However, even with the non-linearity, the model has strong challenges with fully representing the stress data.

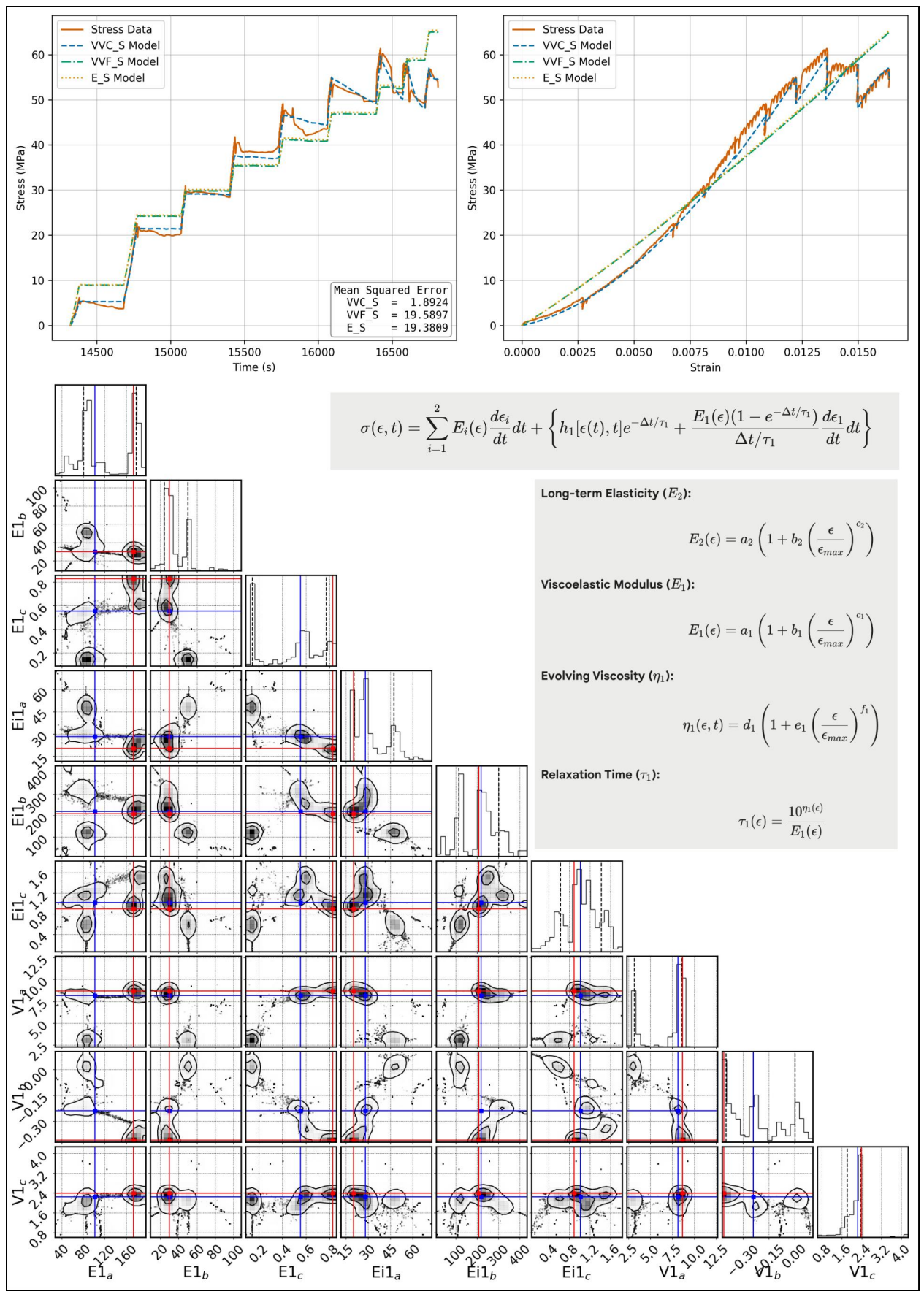

Fig. S16. Experimental stress data was compared to the three different forward models for the strain-controlled sections of experiment p5914 along with the mean square error seen in the insets of the top panel figure. Here, VVC_S is for the viscoelastic with changing viscosity model, VVF_S is for the viscoelastic with fixed viscosity model, and E_S is for the elastic model (with varying elastic modulus with strain). The _S represents that these models had viscosity and the elastic moduli defined as functions of strain. The corner plots for the strain-controlled section of the experiment are presented in bottom panel. The red lines correspond to best fit parameter, while blue line refers to the median of all the MCMC parameters.

Rate-State Friction in GSM Theory

This note provides a rigorous thermodynamic mapping of the empirical Dieterich-Ruina Rate-and-State Friction (RSF) equations [26, 19, 24, 18, 25] into the framework of Generalized Standard Materials (GSM). We demonstrate that the empirical RSF laws violate the integrability conditions required for a single dissipation potential, necessitating a *bipotential* (non-associated) formulation [7, 6] with two independent pseudo-potentials: one for slip velocity and one for state evolution. By decoupling the mechanical and configurational dissipative mechanisms, we derive both the Aging and Slip laws exactly

while guaranteeing strict adherence to the Clausius-Duhem inequality.

Kinematics and State Variables

We consider a representative elementary area of a fault interface. The total displacement jump δ is additively decomposed into an elastic component δ^e and an inelastic (plastic) slip component δ^p:

$$\delta = \delta^e + \delta^p \tag{S11}$$

The thermodynamic state is described by $\mathbf{q} = (\delta^e, \theta)$, where θ [s] is the internal state variable representing the average contact lifetime of the asperity population.

The Helmholtz Free Energy (Ψ)

The reversible energy storage is governed by the Helmholtz free energy density:

$$\Psi(\delta^e, \theta) = \frac{1}{2}k(\delta^e)^2 + U(\theta) \tag{S12}$$

where k is the effective elastic stiffness and $U(\theta)$ is the internal surface energy density. Consistent with the logarithmic RSF framework, we adopt:

$$U(\theta) = -b\sigma_n D_c \ln\left(\frac{\theta}{\theta_0}\right) \tag{S13}$$

yielding the thermodynamic forces:

$$\tau = \frac{\partial \Psi}{\partial \delta^e} = k\delta^e \tag{S14}$$

$$A_\theta = -\frac{\partial \Psi}{\partial \theta} = \frac{b\sigma_n D_c}{\theta} \tag{S15}$$

Here τ is the macroscopic shear stress and A_θ is the state affinity, representing the configurational force driving contact maturation. Note that $A_\theta > 0$ for all admissible $\theta > 0$ and is $\mathcal{O}(1)$, scaling with the state evolution parameter b.

The Bipotential Framework (Non-Associated Dissipation)

The empirical RSF equations cannot be derived from a single dissipation potential $\Phi^*(\tau, A_\theta)$ because they violate the integrability (Maxwell) condition:

$$\frac{\partial V}{\partial A_\theta} \neq \frac{\partial \dot{\theta}}{\partial \tau} \tag{S16}$$

Specifically, the empirical slip law $V(\tau)$ has no dependence on A_θ (the left side vanishes), whereas $\dot{\theta}$ depends on τ through V (the right side is non-zero).

Therefore, RSF constitutes a **non-associated** dissipative system requiring two distinct dual dissipation potentials [4, 15]:

1. **Slip Potential** $\Phi_V^*(\tau;\theta)$: Governs the mechanical slip rate, with θ as a parameter.
2. **Healing Potential** $\Phi_\theta^*(A_\theta;\tau,\theta)$: Governs the state evolution kinetics, with the current stress state (or equivalently V) appearing as a parametric variable.

The kinetic equations are given by the decoupled normality rules:

$$V \equiv \dot{\delta}^p = \frac{\partial \Phi_V^*}{\partial \tau} \tag{S17}$$

$$\dot{\theta} = \frac{\partial \Phi_\theta^*}{\partial A_\theta} \tag{S18}$$

We define the state-dependent reference yield surface (threshold stress):

$$\tau_{th}(\theta) = \sigma_n \left[f_0 + b\ln\left(\frac{\theta}{\theta_0}\right)\right] \tag{S19}$$

where σ_n is the effective normal stress (positive in compression), f_0 is the reference friction coefficient, and a, b are the direct and evolution effect parameters, respectively.

Derivation of the Dieterich Aging Law

To recover the Aging Law exactly, we define the following bipotential pair:

Slip Potential:

$$\Phi_V^*(\tau;\theta) = a\sigma_n V_0 \exp\left(\frac{\tau - \tau_{th}(\theta)}{a\sigma_n}\right) \tag{S20}$$

Healing Potential:

$$\Phi_\theta^*(A_\theta;\tau,\theta) = A_\theta\left[1 - \frac{\theta V_0}{D_c}\exp\left(\frac{\tau - \tau_{th}(\theta)}{a\sigma_n}\right)\right] = A_\theta\left(1 - \frac{V\theta}{D_c}\right) \tag{S21}$$

where V in Eq. S21 is understood as the instantaneous slip velocity determined by the current τ via Eq. S17.

Slip Velocity (V)

Applying Eq. S17 to Eq. S20:

$$V = \frac{\partial \Phi_V^*}{\partial \tau} = V_0 \exp\left(\frac{\tau - \tau_{th}(\theta)}{a\sigma_n}\right) \tag{S22}$$

Inverting this yields the exact RSF stress equation:

$$\tau = \sigma_n\left[f_0 + a\ln\left(\frac{V}{V_0}\right) + b\ln\left(\frac{\theta}{\theta_0}\right)\right] \tag{S23}$$

Note that no asymptotic approximation is required here since the mechanical response is *exactly* decoupled from the state affinity A_θ by construction.

State Evolution

Applying Eq. S18 to Eq. S21:

$$\dot{\theta} = \frac{\partial \Phi_\theta^*}{\partial A_\theta} = 1 - \frac{V\theta}{D_c} \tag{S24}$$

This is the exact Dieterich Aging Law. The non-associated structure is evident in the equations since the state evolution depends on the mechanical state (V), but is derived from a potential distinct from that governing the slip itself.

Derivation of the Ruina Slip Law

The Slip Law requires zero healing when $V = 0$. We retain the same slip potential Φ_V^* (Eq. S20) but modify the healing potential:

Healing Potential (Slip Law):

$$\Phi_\theta^*(A_\theta;\tau,\theta) = -A_\theta\left[\frac{V\theta}{D_c}\ln\left(\frac{V\theta}{D_c}\right)\right] \tag{S25}$$

where V is again the instantaneous velocity from Eq. S22.

State Evolution
Differentiating Eq. S25:

$$\dot{\theta} = \frac{\partial \Phi_\theta^*}{\partial A_\theta} = -\frac{V\theta}{D_c} \ln\left(\frac{V\theta}{D_c}\right) \tag{S26}$$

This is the exact Ruina Slip Law. When $V \to 0$, the term $V \ln(V) \to 0$, enforcing $\dot{\theta} = 0$ as required.

Thermodynamic Admissibility and High-Velocity Breakdown

The foundational requirement of any physically sound constitutive model is adherence to the Second Law of Thermodynamics. In the context of isothermal Generalized Standard Materials, the intrinsic dissipation $\mathscr{D}$ must satisfy the Clausius-Duhem inequality for all physically admissible states:

$$\mathscr{D} = \tau V + A_\theta \dot{\theta} \geq 0 \tag{S27}$$

Thermodynamic Breakdown of the Slip Law
Evaluating the Ruina Slip Law exposes a critical theoretical limitation. Substituting the exact state evolution (Eq. S26) into the dissipation inequality yields:

$$\begin{aligned}\mathscr{D}_{Slip} &= \tau V - \frac{b\sigma_n D_c}{\theta}\left[\frac{V\theta}{D_c}\ln\left(\frac{V\theta}{D_c}\right)\right] \\ &= V\left[\tau - b\sigma_n \ln\left(\frac{V\theta}{D_c}\right)\right]\end{aligned} \tag{S28}$$

We explicitly expand the logarithmic term and substitute the exact macroscopic stress τ (Eq. S23):

$$\begin{aligned}\mathscr{D}_{Slip} = V\sigma_n \Bigg[&\left(f_0 + a\ln\left(\frac{V}{V_0}\right) + b\ln\left(\frac{\theta}{\theta_0}\right)\right) \\ &- b\ln(V) - b\ln(\theta) + b\ln(D_c)\Bigg]\end{aligned} \tag{S29}$$

The state variable θ cancels identically from the dissipation equation $(+b\ln(\theta) - b\ln(\theta) = 0)$. Expanding the reference velocity and grouping the remaining terms results in an exact, purely velocity-dependent expression for the intrinsic dissipation:

$$\begin{aligned}\mathscr{D}_{Slip} = V\sigma_n \Big[&f_0 + (a-b)\ln(V) - a\ln(V_0) \\ &- b\ln(\theta_0) + b\ln(D_c)\Big]\end{aligned} \tag{S30}$$

This exact thermodynamic reduction reveals a fundamental physical violation at extreme slip rates. In the seismogenic regime, faults are characteristically velocity-weakening, necessitating that $b > a$. Consequently, the coefficient $(a - b)$ is strictly negative.

As the slip velocity approaches high velocity speeds ($V \to \infty$), the term $(a - b)\ln(V) \to -\infty$. Since all other terms within the bracket are bounded constants, the total intrinsic dissipation inevitably becomes negative at sufficiently high velocities (though this may not be physically relevant for real faults) :

$$\lim_{V\to\infty} \mathscr{D}_{Slip} \to -\infty \quad \text{for } b > a \tag{S31}$$

This proves that the unregularized empirical Ruina Slip Law is not globally thermodynamically admissible. While the bipotential GSM framework flawlessly maps the algebraic structure of the empirical equations, it formally dictates that the logarithmic RSF formulation cannot govern dynamic rupture. To prevent violations of the Second Law of Thermodynamics, computational models must regularize the slip potential at high velocities (e.g., via sinh-based reaction rate theory) to ensure bounded, positive dissipation.

Admissibility of the Aging Law
For the Dieterich Aging Law, substituting the state affinity (Eq. S15) and the exact state evolution (Eq. S24) yields:

$$\begin{aligned}\mathscr{D}_{Aging} &= \tau V + \frac{b\sigma_n D_c}{\theta}\left(1 - \frac{V\theta}{D_c}\right) \\ &= V(\tau - b\sigma_n) + \frac{b\sigma_n D_c}{\theta}\end{aligned} \tag{S32}$$

Substituting the macroscopic stress τ (Eq. S23) and grouping terms gives:

$$\mathscr{D}_{Aging} = V\sigma_n\left[f_0 - b + a\ln\left(\frac{V}{V_0}\right) + b\ln\left(\frac{\theta}{\theta_0}\right)\right] + \frac{b\sigma_n D_c}{\theta} \tag{S33}$$

The term $b\sigma_n D_c/\theta$ represents the rate of free energy dissipation associated with autonomous contact aging and is strictly positive for $\theta > 0$. During slip-weakening ($\dot{\theta} < 0$), the stored surface energy of the contacts is released, contributing negatively to the local dissipation budget, but the positive mechanical work (τV) globally dominates. The Aging Law thus remains thermodynamically admissible across standard operational regimes, though similar to the Slip law above, the aging law has the identical high-V breakdown.

Regularization for Global Thermodynamic Admissibility

The previous section demonstrated that the classical logarithmic Slip Law inevitably violates the Clausius-Duhem inequality at dynamic rupture velocities ($V \to \infty$). To rectify this, the bipotential framework must be regularized at both asymptotic limits. We apply absolute reaction-rate theory to resolve the singularity at $V \to 0$, and demonstrate that elastodynamic (radiation) damping is required to enforce thermodynamic consistency at $V \to \infty$.

Reaction-Rate Regularization (The sinh Law)
The empirical logarithmic slip equation implies a unidirectional crossing of asperity energy barriers, resulting in an anti-physical singularity at $V = 0$. Following transition-state theory [8, 14] and work in numerical fault modeling [16, 25], the slip potential must account for both forward and backward microscopic jumps.

We construct a regularized, globally convex slip potential using the hyperbolic cosine:

$$\Phi_V^*(\tau;\theta) = 2a\sigma_n V_0\left[\cosh\left(\frac{\tau - \tau_{th}(\theta)}{a\sigma_n}\right) - 1\right] \tag{S34}$$

The subtraction of unity ensures that $\Phi_V^*(\tau_{th}, \theta) = 0$ and $\Phi_V^* \geq 0$ for all admissible states.

Applying the normality rule (Eq. S17) yields the regularized slip equation:

$$V = \frac{\partial \Phi_V^*}{\partial \tau} = 2V_0 \sinh\left(\frac{\tau - \tau_{th}(\theta)}{a\sigma_n}\right) \tag{S35}$$

Inverting this provides the exact, regularized macroscopic shear stress:

$$\begin{aligned}\tau &= \tau_{th}(\theta) + a\sigma_n \operatorname{arcsinh}\left(\frac{V}{2V_0}\right) \\ &= \sigma_n\left[f_0 + a\operatorname{arcsinh}\left(\frac{V}{2V_0}\right) + b\ln\left(\frac{\theta}{\theta_0}\right)\right]\end{aligned} \tag{S36}$$

This regularization resolves the zero-velocity singularity, as $\operatorname{arcsinh}(0) = 0$, allowing the interface to arrest without generating infinite negative stress.

The Persistence of the High-Velocity Violation
While the sinh potential grounds the near-zero velocity regime physically, we must evaluate its high-velocity thermodynamic behavior. Substituting the regularized stress (Eq. S36) into the dissipation expression for the Slip Law yields:

$$\mathscr{D}_{Reg} = V\sigma_n \left[f_0 + a\,\text{arcsinh}\left(\frac{V}{2V_0}\right) + b\ln\left(\frac{\theta}{\theta_0}\right) - b\ln\left(\frac{V\theta}{D_c}\right)\right] \tag{S37}$$

Once again, the state variable θ cancels identically. Regrouping terms gives:

$$\mathscr{D}_{Reg} = V\sigma_n \Big[f_0 + a\,\text{arcsinh}\left(\frac{V}{2V_0}\right) - b\ln(V) - b\ln(\theta_0) + b\ln(D_c)\Big] \tag{S38}$$

To evaluate the dynamic rupture limit, we employ the asymptotic expansion of the inverse hyperbolic sine as $V \to \infty$:

$$\text{arcsinh}\left(\frac{V}{2V_0}\right) = \ln\left(\frac{V}{2V_0} + \sqrt{\left(\frac{V}{2V_0}\right)^2 + 1}\right) \xrightarrow{V\to\infty} \ln\left(\frac{V}{V_0}\right) \tag{S39}$$

Substituting this limit into the dissipation expression yields:

$$\lim_{V\to\infty} \mathscr{D}_{Reg} \approx V\sigma_n\left[(a-b)\ln(V) - a\ln(V_0) - b\ln(\theta_0) + b\ln(D_c)\right] \tag{S40}$$

This is a critical theoretical result: **the sinh regularization fails to prevent the Second Law violation at high velocities**. Because the physical transition-state barrier is entirely overcome at extreme shear, the sinh function reverts to logarithmic scaling. In the velocity-weakening regime ($b > a$), the $(a-b)\ln(V)$ term still drives the total intrinsic dissipation to negative infinity:

$$\lim_{V\to\infty} \mathscr{D}_{Reg} \to -\infty \quad \text{for } b > a \tag{S41}$$

Thermodynamic Resolution via Viscous Damping
The mathematical failure of both logarithmic and sinh potentials at high slip rates implies that frictional constitutive laws alone cannot describe dynamic rupture. A parallel dissipative mechanism is required to bound the energy budget.

Physically, at seismic slip rates ($\sim$ m/s), the fault interface radiates elastodynamic energy (radiation damping) and experiences phonon drag [24]. In the GSM framework, this is incorporated by adding a linear viscous stress ηV to the macroscopic stress, where $\eta \approx G/2c_s$ (shear modulus G, shear wave speed c_s).

The fully regularized macroscopic stress becomes:

$$\tau_{total} = \underbrace{\sigma_n\left[f_0 + a\,\text{arcsinh}\left(\frac{V}{2V_0}\right) + b\ln\left(\frac{\theta}{\theta_0}\right)\right]}_{\tau_{RSF}} + \eta V \tag{S42}$$

The corresponding total intrinsic dissipation incorporates the viscous penalty:

$$\mathscr{D}_{Total} = \underbrace{\tau_{RSF}V + A_\theta\dot{\theta}}_{\mathscr{D}_{Reg}} + \eta V^2 \tag{S43}$$

At the dynamic rupture limit ($V \to \infty$), the dominant terms are:

$$\mathscr{D}_{Total} \approx \eta V^2 - \sigma_n|a-b|V\ln(V) + \mathscr{O}(V) \tag{S44}$$

Because the polynomial viscous dissipation (ηV^2) grows strictly faster than the logarithmic deficit ($V\ln V$), the quadratic term unconditionally dominates. Therefore:

$$\lim_{V\to\infty} \mathscr{D}_{Total} \to +\infty \geq 0 \tag{S45}$$

This proves that while the sinh potential ensures physical admissibility at $V \to 0$, the inclusion of high-velocity viscous (radiation) damping is strictly required to ensure global thermodynamic admissibility of the Rate-and-State framework across all slip regimes.

References

1. Takuya Akiba, Shotaro Sano, Toshihiko Yanase, Takeru Ohta, and Masanori Koyama. Optuna: A next-generation hyperparameter optimization framework. In *Proceedings of the 25th ACM SIGKDD international conference on knowledge discovery & data mining*, pages 2623–2631, 2019.
2. Anthony Atkinson, Alexander Donev, and Randall Tobias. *Optimum experimental designs, with SAS*, volume 34. OUP Oxford, 2007.
3. Steven L Brunton, Joshua L Proctor, and J Nathan Kutz. Discovering governing equations from data by sparse identification of nonlinear dynamical systems. *Proceedings of the National Academy of Sciences*, 113(15):3932–3937, 2016.
4. I. F. Collins and G. T. Houlsby. Application of thermomechanical principles to the modelling of geotechnical materials. *Proceedings of the Royal Society of London. Series A: Mathematical, Physical and Engineering Sciences*, 453(1964):1975–2001, 1997.
5. F Michael Conway, James W Vallance, William I Rose, Glen W Johns, and Sergio Paniagua. Cerro quemado, guatemala: the volcanic history and hazards of an exogenous volcanic dome complex. *Journal of volcanology and geothermal research*, 52(4):303–323, 1992.
6. Géry de Saxcé and Lahbib Bousshine. Implicit standard materials. In *Inelastic Behaviour of Structures under Variable Repeated Loads: Direct Analysis Methods*, pages 59–76. Springer, 2002.
7. Géry de Saxcé and Zhi-Qiang Feng. New inequality and functional for contact with friction: the implicit standard material approach. *Journal of Structural Mechanics*, 19(3):301–325, 1991.
8. Henry Eyring. Viscosity, plasticity, and diffusion as examples of absolute reaction rates. *The Journal of Chemical Physics*, 4(4):283–291, 1936.
9. Moritz Flaschel, Paul Steinmann, Laura De Lorenzis, and Ellen Kuhl. Convex neural networks learn generalized standard material models. *Journal of the Mechanics and Physics of Solids*, 200:106103, 2025.
10. Daniel Foreman-Mackey, David W Hogg, Dustin Lang, and Jonathan Goodman. emcee: the mcmc hammer. *Publications of the Astronomical Society of the Pacific*, 125(925):306–312, 2013.
11. Ragnar Frisch and Frederick V. Waugh. Partial time regressions as compared with individual trends. *Econometrica*, 1(4):387–401, 1933.
12. Alexandre Grêt, Roel Snieder, and John Scales. Time-lapse monitoring of rock properties with coda wave interferometry. *Journal of Geophysical Research: Solid Earth*, 111(B3):B03305, 2006.

13. Bernard Halphen and Quoc Son Nguyen. Sur les matériaux standard généralisés. *Journal de Mécanique*, 14(1):39–63, 1975.
14. F. Heslot, T. Baumberger, B. Perrin, B. Caroli, and C. Caroli. Creep, stick-slip, and dry-friction dynamics: Experiments and a heuristic model. *Physical Review E*, 49(6):4973–4988, 1994.
15. G. T. Houlsby and A. M. Puzrin. *Principles of Hyperplasticity: An Approach to Plasticity Theory Based on Thermodynamic Principles*. Springer Science & Business Media, 2006.
16. Nadia Lapusta, James R. Rice, Yehuda Ben-Zion, and Gutuan Zheng. Elastodynamic analysis for slow tectonic loading with spontaneous rupture episodes on faults with rate- and state-dependent friction. *Journal of Geophysical Research: Solid Earth*, 105(B10):23765–23789, 2000.
17. Michael C. Lovell. Seasonal adjustment of economic time series and multiple regression analysis. *Journal of the American Statistical Association*, 58(304):993–1010, 1963.
18. Chris Marone. Laboratory-derived friction laws and their application to seismic faulting. *Annual Review of Earth and Planetary Sciences*, 26(1):643–696, 1998.
19. Chris J. Marone, C. H. Scholz, and Roger Bilham. On the mechanics of earthquake afterslip. *Journal of Geophysical Research: Solid Earth*, 96(B5):8441–8452, 1991.
20. Filippo Masi, Ioannis Stefanou, Paolo Vannucci, and Victor Maffi-Berthier. Thermodynamics-based artificial neural networks for constitutive modeling. *Journal of the Mechanics and Physics of Solids*, 147:104277, 2021.
21. Amos Nur. Effects of stress on velocity anisotropy in rocks with cracks. *Journal of Geophysical Research*, 76(8):2022–2034, 1971.
22. Richard J O'Connell and Bernard Budiansky. Seismic velocities in dry and saturated cracked solids. *Journal of Geophysical Research*, 79(35):5412–5426, 1974.
23. Nicole L Ramo, Christian M Puttlitz, and Kevin L Troyer. The development and validation of a numerical integration method for non-linear viscoelastic modeling. *PLoS One*, 13(1):e0190137, 2018.
24. James R. Rice. Spatio-temporal complexity of slip on a fault. *Journal of Geophysical Research: Solid Earth*, 98(B6):9885–9907, 1993.
25. James R. Rice, Nadia Lapusta, and K. Ranjith. Rate and state dependent friction and the stability of sliding between elastically deformable solids. *Journal of the Mechanics and Physics of Solids*, 49(9):1865–1898, 2001.
26. Andy Ruina. Slip instability and state variable friction laws. *Journal of Geophysical Research: Solid Earth*, 88(B12):10359–10370, 1983.
27. Lauren N Schaefer, Thomas Oommen, Claudia Corazzato, Alessandro Tibaldi, Rudiger Escobar-Wolf, and William I Rose. An integrated field-numerical approach to assess slope stability hazards at volcanoes: the example of pacaya, guatemala. *Bulletin of volcanology*, 75(6):720, 2013.
28. Christoph Sens-Schönfelder and Ulrich Wegler. Passive image interferometry and seasonal variations of seismic velocities at merapi volcano, indonesia. *Geophysical Research Letters*, 33(21):L21302, 2006.
29. Roel Snieder. The theory of coda wave interferometry. *Pure and Applied Geophysics*, 163(2–3):455–473, 2006.
30. Roel Snieder, Alexandre Grêt, Huub Douma, and John Scales. Coda wave interferometry for estimating nonlinear behavior in seismic velocity. *Science*, 295(5563):2253–2255, 2002.
31. Paul Steinmann and Kenneth Runesson. *The Catalogue of Computational Material Models*. Springer, 2021.
32. M Nafi Toksöz, David H Johnston, and A Timur. Attenuation of seismic waves in dry and saturated rocks: I. laboratory measurements. *Geophysics*, 44(4):681–690, 1979.
